# Hemodynamic Changes During Physiological and Pharmacological Stress Testing in Healthy Subjects, Aortic Stenosis and Aortic Coarctation Patients–A Systematic Review and Meta-Analysis

**DOI:** 10.3389/fcvm.2019.00043

**Published:** 2019-04-10

**Authors:** Kilian Runte, Kay Brosien, Maximilian Salcher-Konrad, Charlotte Schubert, Leonid Goubergrits, Sebastian Kelle, Stephan Schubert, Felix Berger, Titus Kuehne, Marcus Kelm

**Affiliations:** ^1^Institute for Imaging Science and Computational Modelling in Cardiovascular Medicine, Charité–Universitätsmedizin Berlin, Berlin, Germany; ^2^Department of Congenital Heart Disease, German Heart Center Berlin, Berlin, Germany; ^3^Personal Social Services Research Unit, London School of Economics and Political Science, London, United Kingdom; ^4^LSE Health, London School of Economics and Political Science, London, United Kingdom; ^5^Department of Internal Medicine/Cardiology, German Heart Center Berlin, Berlin, Germany; ^6^Department of Internal Medicine/Cardiology, Charité–Universitätsmedizin Berlin, Berlin, Germany; ^7^German Center for Cardiovascular Research, Partner Site Berlin, Berlin, Germany

**Keywords:** hemodynamics, exercise testing, dobutamine stress, healthy subjects, aortic stenosis, aortic coarctation, meta-analysis, systematic review

## Abstract

**Introduction:** Exercise testing has become a diagnostic standard in the evaluation and management of heart disease. While different methods of exercise and pharmacological stress testing exist, only little is known about their comparability. We aimed to assess hemodynamic changes during dynamic exercise, isometric exercise, and dobutamine stress testing at different stress intensities in healthy subjects and patients with aortic stenosis (AS) and aortic coarctation (CoA).

**Methods:** A systematic literature search (PROSPERO 2017:CRD42017078608) in MEDLINE of interventional trials was conducted to identify eligible studies providing evidence of changes in hemodynamic parameters under different stress conditions acquired by MRI or echocardiography. A random effects model was used to estimate pooled mean changes in hemodynamics.

**Results:** One hundred and twenty-eight study arms with a total of 3,139 stress-examinations were included. In healthy subjects/(where available) in AS, pooled mean changes (95% CIs) during light dynamic stress were 31.78 (27.82–35.74) bpm in heart rate (HR) and 6.59 (2.58–10.61) ml in stroke volume (SV). Changes during light pharmacological stress were 13.71 (7.87–19.56)/14.0 (9.82–18.18) bpm in HR, and 5.47 (0.3–10.63)/8.0 (3.82–12.18) ml in SV. Changes during light isometric stress were 18.44 (10.74–26.14)/5.0 (−1.17–11.17) bpm in HR and −4.17 (−14.37–6.03)/−4.0 (−16.43–8.43) ml in SV. Changes during moderate dynamic stress were 49.57 (40.03–59.1)/46.45 (42.63–50.27) bpm in HR and 11.64 (5.87–17.42) ml in SV. During moderate pharmacological stress, changes in HR were 42.83 (36.94–48.72)/18.66 (2.38–34.93) bpm and in SV 6.29 (−2.0–14.58)/13.11 (7.99–18.23) ml. During high intensity dynamic stress changes in HR were 89.31 (81.46–97.17)/55.32 (47.31–63.33) bpm and in SV 21.31 (13.42–29.21)/−0.96 (−5.27–3.35) ml. During high pharmacological stress, changes in HR were 53.58 (36.53–70.64)/42.52 (32.77–52.28) bpm, and in SV 0.98 (−9.32–11.27)/14.06 (−1.62–29.74) ml. HR increase and age were inversely correlated at high stress intensities. In CoA, evidence was limited to single studies.

**Conclusion:** This systematic review and meta-analysis presents pooled hemodynamic changes under light, moderate and high intensity exercise and pharmacological stress, while considering the potential influence of age. Despite limited availability of comparative studies, the reference values presented in this review allow estimation of the expected individual range of a circulatory response in healthy individuals and patients with AS and may contribute to future study planning and patient-specific models even when stress testing is contraindicated.

## Introduction

Cardiac stress testing is a major diagnostic tool in the evaluation of heart disease (1). Stress testing in combination with imaging techniques is not only recommended by current guidelines to unmask myocardial ischemia (2), it is also used in valvular heart disease (3) and decision making processes in congenital heart disease (4, 5). Several methods for measuring physiological responses to stress and exercise exist, including dynamic and isometric exercise as well as pharmacological stress testing (6, 7), and a lack of comparability between different stress laboratories and populations has been noted (8).

While dynamic stress testing is generally considered the most physiological type of stress (7), it is time consuming and of limited use in some situations, e.g., in patients who are unable to exercise adequately (9). Furthermore, this method requires standardized equipment, with a lack of comparability between different diagnostic set-ups having been described as problematic (8). The combined use of dynamic stress testing with non-invasive imaging procedures such as magnetic resonance imaging (MRI) or echocardiography has been limited due to motion artifacts (9–11).

Pharmacological stress testing can be indicated when dynamic testing cannot be performed due to the state of disease or the setting of the examination. Dobutamine is widely used as a pharmacological stressor to assess the hemodynamic response in valvular heart disease, including aortic stenosis (AS), and in congenital heart disease (3, 12, 13). Through its main effects on myocardial beta-1 adrenergic receptors and its additional effects on alpha-1 and beta-2 receptors, dobutamine leads to a positive inotropic and chronotropic reaction and may also involve minor vasodilating effects (14, 15). Through its effects on heart rate (HR), stroke volume (SV), and vascular resistance it can be used to simulate responses similar to those measured during dynamic exercise (16). However, the effect of dobutamine is possibly subject to changes at higher doses, resulting in a further rise in HR with reduced diastolic filling time and lower end-diastolic volumes. At the point where compensation by a corresponding decrease in end-systolic volume is no longer possible, the increased SV begins to fall whereas further increases in cardiac output (CO) are mainly caused by a rise in HR, as previously reported for healthy controls receiving incremental dobutamine infusion (17).

Cardiovascular effects of isometric exercise testing have been described since the early 1960s (18). Squeezing a handgrip dynamometer can be performed easily without causing artifacts affecting image quality, and MRI-safe handgrip devices have accordingly become more popular during the past decades (19, 20). Nevertheless, isometric exercise mainly imposes high afterload on the ventricle, distinguishing it from the hemodynamic reactions during dynamic exercise (6, 21). While its potential role in the evaluation of coronary artery disease has been described (22), current guidelines do not recommend the routine use of isometric exercise in cardiac diagnostics (2, 23).

In AS and aortic coarctation (CoA), a congenital narrowing of the aorta, dynamic, and pharmacological stress testing was included in clinical guidelines for the assessment of pressure gradients and cardiovascular responses (24, 25). In AS, the information obtained from stress testing is used in the decision-making process for the timing of surgical or interventional treatment procedures (26, 27). Patients with AS–even when asymptomatic–are known to show abnormal hemodynamic responses with a lower SV and CO than controls (28). In CoA, pharmacological dobutamine stress can be useful in order to maintain adequate blood pressure and HR in patients who are anesthetized or sedated during the heart catheterization procedure, allowing reliable measurement of pressure gradients before and after treatment (29, 30).

Physiological simulations and computational models carry promising potential to provide further insights into hemodynamic interactions between cardiac, vascular, and neurohumoral responses (31, 32). In order to bring such tools to diagnostic relevance and to simulate disease specific responses they need to be capable of correctly simulating common stress activity levels. Reliable values of stress-induced changes in HR, SV, and CO as well as systolic ejection time (SET) and time to peak aortic flow (TTP) are of particular interest for advanced diagnostics. The quality of existing evidence reporting on these parameters remains largely unknown. We aimed to summarize hemodynamic changes caused by dynamic exercise, isometric exercise, and pharmacological stress testing at different stress intensities in healthy subjects, patients with AS, and patients with CoA by synthesizing available evidence.

## Materials and Methods

### Search Strategy

A review protocol was developed and publicized on the PROSPERO register of systematic reviews (PROSPERO 2017:CRD42017078608). The protocol was developed with the aim of identifying all studies in which evidence of the hemodynamic parameters HR, SV, CO, SET, or TTP under resting and stress conditions for dynamic, isometric, and pharmacological stress testing could be found in healthy individuals, patients with AS and patients with CoA. We focused on studies reporting hemodynamic parameters acquired by MRI or echocardiography. Outlines of the questions addressed by this review are shown in the standardized scheme addressing patient population, interventions, comparators, outcomes, and study design (PICOS) in Table 1. No previous meta-analyses addressing these specific questions were identified. The search was conducted by a member of the research team (KR) in MEDLINE (via PubMed) using previously specified search terms (Table S1 in the data supplement). No relevant deviations were found compared to an Embase query. Publications known from preliminary searches were added. Date of the final search was 05 November 2017.

**Table 1:** PICOS scheme.

**PICOS**	
Patient population	Healthy controls undergoing MRI or echocardiography Aortic coarctation patients undergoing MRI or echocardiography Aortic stenosis patients undergoing MRI or echocardiography
Interventions	Dynamic exercise Dobutamine infusion Isometric exercise
Comparators	Resting state
Outcomes	Primary Heart rate [bpm] Blood flow/stroke volume [ml] Cardiac output [l/min] Additional Systolic ejection time [ms] Time to peak aortic flow rate [ms]
Study design	Interventional studies with or without control group

### Study Selection and Quality Assessment

Studies that met the inclusion criteria (as set out in the PICOS table) were included. Eligible records were reports of interventional studies, either with or without a control group. They were included in the quantitative analysis if at least HR and another outcome parameter (SV, CO, SET, TTP) under resting and stress conditions could be extracted. We limited our search to studies published after 1985 to avoid errors in measurements resulting from lower diagnostic accuracy of outdated imaging devices. Exclusion criteria were met when studies were not available in English, German nor as full texts within the institutional subscriptions or the National Library license or were not conducted in humans. Furthermore, we excluded studies that (a) had measurement locations other than the ascending aorta or the left ventricle, (b) used other measurement techniques than MRI or echocardiography, or (c) if stress types were not dynamic exercise, upper-limb isometric exercise, or dobutamine infusion as pharmacological stress. Study arms with < 10 subjects were not included. Reviews, letters, comments, conference posters, and single case reports were excluded. Following the criteria described, articles were scanned on title and abstract level before full texts were retrieved. In studies providing evidence of different cohorts or intensity levels, every cohort and intensity level was included as a separate study arm. Each study was assessed using a modified version of the Downs and Black checklist (33). The tool was chosen as recommended by the Cochrane handbook for assessing the methodological quality of non-randomized studies (34) and was suitable for different study types. We adapted the checklist by excluding irrelevant items according to study type and characteristics. Each study was assessed for reporting (1–4, 6–8, 10), external validity (11–13), internal validity including study and selection bias (16, 19–22), and power (27). Studies involving a control group were additionally assessed on distribution and adjustment for confounding variables (5, 25). Whenever follow-up was part of a study, losses to follow-up were evaluated (9). Each paper was reviewed by one reviewer (CS) and verified by a second reviewer (KR). Cases of disagreement were discussed with a third reviewer (MK). Cut-offs were determined by dividing the maximum possible points into thirds. Therefore, a study in which 17 or 18 items were assessed was considered to be of low methodological quality if it achieved 0 to 5 points, of moderate quality if it achieved 6 to 10 points and of high quality at more than 11 points. In cases of 19 or 20 applicable items a study was judged to have low quality when the score was 0 to 6, moderate quality when it was 7 to 13 points and high quality with a score above 14 points.

### Data Extraction

Data were extracted by a member of the research team (KR). Means and standard deviations of HR, SV, CO, SET, and TTP were used if available under resting and stress conditions. If no information on the variance was available, studies were not included in the analysis. If studies provided indexed stroke volume or the cardiac index and body surface area (BSA), the absolute SV and CO were calculated. The data extracted also included the description of study participants (sex, age, BSA, BMI, state of disease, and left-ventricular function), characteristics of the interventions as to type and intensity of the stress test and the image modality used.

### Intensity Classification

Studies were categorized as light, moderate and high intensity according to intensity classifications of dynamic exercise. The following indications provided the basis for classification of stress intensity:

1. Metabolic equivalents (METs), (35)
2. Intensity in watts (W) during ergometric exercise (assuming a body weight of 60–80 kg), (35)
3. Stage of standard Bruce protocol during treadmill exercise (7)
4. Percentage of age predicted maximal heart rate (HR_max_ = 220–age in years) (36),
5. Statement of study authors about the intensity level.

Following Jetté et al. we considered an intensity of ≤ 4 METs as light, 5 to 8 METs as moderate and >8 METs as high intensity. The equivalence of watts to METs can be assumed as follows for a person with a body weight of 60–80 kg: 50 W ≜ 3.2–4.3 METs; 75 W ≜ 4.3–5.7 METs; 100 W ≜ 5.4–7.1 METs; 125 W ≜ 6.4–8.6 METs; 150 W ≜ 7.5–10 METs (35). For the standard Bruce protocol similar assumptions were made: Stage 1 ≜ 4.5 METs; Stage 2 ≜ 7 METs; Stage 3 ≜ 10.5 METs (7). In accordance with common classifications, intensity of pharmacologic stress was considered as light for low dose infusion of dobutamine of 0–10 μg/kg/min, as moderate for 11–20 μg/kg/min and as high for a dose exceeding 20 μg/kg/min (12, 37–39). Isometric exercise tests were always classified as light intensity because static contraction causes only little increase in HR or CO while it mainly affects mean arterial pressure and is not expected to reach the changes of higher levels of dynamic exercise (21). Applying these criteria we created the stress intensities represented in Table 2.

**Table 2:** Intensity levels of stress testing.

**Intensity**	**Dynamic exercise**		**Dobutamine stress**	**Isometric exercise**
Light	METs:	≤4 METs	0–10 μg/kg/min	All
	Ergometer:	50 W		
	Treadmill:	Stage 1 BP		
	HR_max_:	≤54%		
	Statement:	Light		
Moderate	METs:	5–8 METs	11–20 μg/kg/min	–
	Ergometer:	75–125 W		
	Treadmill:	Stage 2 BP		
	HR_max_:	55–84%		
	Statement:	Submaximal/ moderate		
High	METs:	> 8 METs	>20 μg/kg/min	–
	Ergometer:	> 125 W (< 70 kg: > 100 W)	HR_max_: ≥ 85% Symptoms	
	Treadmill:	≥ stage 3 BP		
	HR_max_:	≥ 85%		
	Statement:	Exhaustion		

*METs indicates metabolic equivalents (1MET = 3.5 ml O_2_kg^−1^min^−1^); W, watt; BP, standard Bruce protocol; HR_max_, age-predicted maximal heart rate (= 220–age in years)*.

### Statistical Analysis

Primary outcomes were pooled mean differences of HR, SV, CO, and SET between resting and stress conditions. TTP had to be excluded from further analysis as this parameter was only reported in a total number of 5 studies. Pairwise meta-analysis was performed in studies directly comparing different types of stress. Where only single arm data were available, study arms were grouped according to stress type and intensity level and meta-analyzed to obtain pooled estimates of hemodynamic changes. An additional analysis of subgroups was performed in patients with AS and impaired LV function. Mean differences of hemodynamic parameters between rest and stress and respective standard errors of the difference between means were calculated (40). Differences between baseline characteristics of study arms grouped according to stress type were checked by Kruskal-Wallis test, followed by Dunn's test with Bonferroni correction where appropriate. We quantitatively synthesized effects reported in individual studies using a DerSimonian-Laird random effects model (41), as implemented in the “metan” suite of commands in Stata (42). However, this model does not take into account uncertainty in the between-study variance and as a result may underestimate uncertainty of the pooled effect size for samples of studies with high heterogeneity (43). Since we observed high heterogeneity in the results obtained by the DerSimonian-Laird random effects models, we re-ran the analyses with random effects models that use alternative estimators of between-study variance, specifically a restricted maximum likelihood model (44), profile likelihood model (43, 45), and permutations random effects model (46). These models were run as implemented in the “metan” suite of commands in Stata (47). The results obtained from the various models did not differ in any meaningful way from our primary analysis using the DerSimonian-Laird model for either pooled effect size or uncertainty and we therefore only present the results from the pre-planned, primary analysis. We used the Q-statistic to test for statistical heterogeneity. However, since we anticipated a low number of studies for at least some of the intervention-outcome pairs for which meta-analyses were conducted, and generally small sample sizes in individual studies, we did not rely exclusively on the Q-statistic as a measure of heterogeneity. Between-study heterogeneity was also visually assessed through forest plots for all interventions and outcomes, and we quantified the proportion of between-study variation due to true heterogeneity as compared to chance alone through the *I*^2^ statistic (48). Having fitted random-effects models, as per our prior considerations regarding underlying variations in included studies, we then used the *I*^2^ to assess the appropriateness of this selection (49), taking *I*^2^ values of 30% or over to indicate potential significant true heterogeneity (50). Results are shown as absolute mean changes and 95% confidence intervals (CIs) between resting and stress conditions for the different types and intensities of stress. Results of individual studies and pooled estimates for each stress type and intensity are provided in forest plots in the Supplementary Material. We considered the differences between pooled changes for the different stress types as significant if no overlap of their confidence intervals was seen according to Cochrane Collaboration standards (50). A multivariate meta-regression model was used to determine variables potentially influencing outcome parameters. As studies with a wide age range were included, starting from school-aged children, meta-regression analysis was performed to assess the impact of age across the included age spectrum. Potential correlations were investigated through univariate meta-regression. Meta-analyses were executed in STATA, version 15.1 (StataCorp, College Station, Texas, USA), by using the *metan* package.

## Results

### Systematic Review

**Figure 1:**
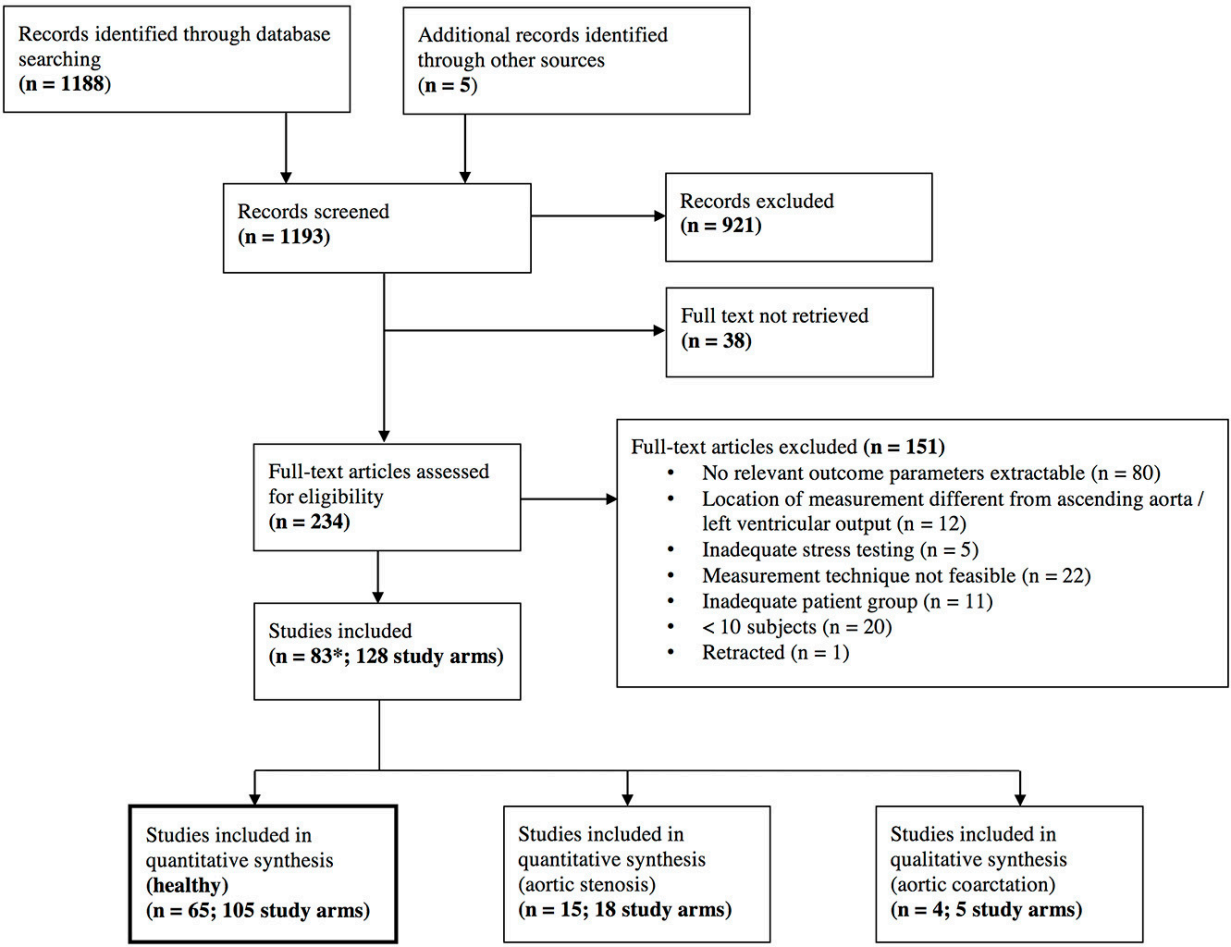
Preferred Reporting Items for Systematic Reviews and Meta-Analyses (PRISMA) flow chart. *One study that was included had different study arms as well for healthy individuals and for aortic stenosis (Donal, January 2011), which explains the different number of total studies included.

The database search resulted in a total of 1,188 references. After screening on title and abstract level, 234 full texts were retrieved. After full-text screening, 83 studies with a total of 128 study arms and 2,812 subjects, and 3,139 stress examinations were included. Details of the study selection process can be found in the PRISMA flow chart (Figure 1) and in the list of included studies in the supplemental material (Tables S2–S4).

For healthy individuals, we found 3 studies (6 study arms) directly comparing dynamic exercise with dobutamine infusion and 2 studies (6 study arms) comparing dynamic with isometric exercise. Additional single study arms were included, resulting in a total of 64 study arms (1,448 stress examinations) for dynamic exercise, 33 study arms (445 stress examinations) for pharmacological stress, and 8 study arms (229 stress examinations) for isometric exercise for pooled analysis (details in Figure S1 in Data Supplement).

For AS, 11 study arms (755 stress examinations) were included for dynamic, 6 study arms (161 stress examinations) for pharmacological, and 1 study arm (22 stress examinations) for isometric stress test. For CoA, evidence was limited to a total number of 5 study arms (79 stress examinations) and no meaningful quantitative analysis of our pre-specified outcomes was possible.

### Study Characteristics

Baseline characteristics for healthy individuals undergoing dynamic, dobutamine, and isometric stress tests are shown in Table 3. Subjects in the dynamic exercise group were significantly younger and had a lower body mass index than subjects in the pharmacological or isometric stress group. Four studies (59 patients) in the dynamic exercise group investigated hemodynamic changes in children younger than 18 years. Baseline characteristics for AS can be found in Table S5 in the data supplement. AS was classified as (a) asymptomatic or symptomatic with preserved left ventricular ejection fraction (LV-EF) (877 patients), and (b) low-flow, low-gradient AS with reduced LV-EF (61 patients). While reporting on severity was inconsistent between studies, group (a) contained studies where the need for aortic valve replacement and the presence of severe AS was mentioned (50 patients) and studies where severity ranged from mild to severe (72 patients). All other studies in (a) were in patients with moderate to severe asymptomatic AS.

**Table 3:** Baseline characteristics for healthy individuals.

	**Dynamic exercise**		**Pharmacological stress**		**Isometric exercise**		**Krus-kal-Wallis-Test**
		**Study arms reporting variable (*N* of tests)**		**Study arms reporting variable (*N* of tests)**		**Study arms reporting variable (*N* of tests)**	***P*-Value**
Total *N* of stress tests		64 (1,448)		33 (445)		8 (229)	
Age, years	30^†^ (24.3–51.1)	61 (1,397)	51^†^ (28.5–58)	28 (395)	46.5 (32.7–53.5)	8 (229)	0.02^*^
Male, %	71.7 (46.2–100)	62 (1,420)	50.0 (40.6–75.0)	27 (384)	83.3 (58.0–100)	7 (217)	0.09
BSA, m^2^	1.87 (1.8–1.9)	22 (572)	1.85 (1.82–1.9)	5 (90)	1.9 (1.9–1.9)	1 (18)	0.69
BMI, kg/m^2^	23.6^†^,^‡^ (22.2–24.8)	39 (854)	25.0^†^ (23.9–26.2)	8 (122)	25.1^†^,^‡^ (24.6–25.5)	6 (169)	0.01^*^
Resting HR, bpm	69 (64–74)	55 (1,256)	66 (66–71)	33 (445)	68.5 (64–73)	8 (229)	0.87
Resting SV, ml	75.5 (64–93)	32 (766)	93 (70–101)	18 (231)	81.8 (71–106)	4 (101)	0.18
Resting CO, l/min	5.5 (4.8–6.2)	37 (837)	5.7 (5.5–6.6)	22 (277)	5.4 (3.9–8.3)	3 (83)	0.53
Resting SET, ms	294 (281–308)	10 (207)	270 (270–270)	1 (20)	283 (269–303)	4 (96)	0.35
Light intensity, %	15.7	12 (228)	23.6	10 (105)	100	8 (229)	
Moderate intensity, %	26.9	20 (389)	46.1	15 (205)			
High intensity, %	57.4	32 (831)	30.3	8 (135)			
Children, %	5.5	5 (79)					
Athletes, %	12.7	9 (184)					

*Values are reported as median (interquartile range). BMI indicates body mass index; BSA, body surface area; HR, heart rate; SV, stroke volume; CO, cardiac output; SET, systolic ejection time*.

* *p < 0.05 overall*.

† *p < 0.05 in pairwise comparison of dynamic exercise and pharmacological stress (Dunn's test)*.

‡ *p < 0.05 in pairwise comparison of dynamic exercise and isometric exercise (Dunn's test)*.

### Quality Assessment

Using the modified Downs and Black checklist 32 studies were judged to be of high methodological quality and 51 of moderate quality. None of the studies included was of low quality. The final quality score of each study is reported in Tables S2–S4 in the data supplements. A detailed overview of all items and their scores is provided in Table S8. The highest scores were achieved in the items pertaining to reporting details and internal validity. All studies reported their aims, hypothesis and main outcomes clearly. Compliance was consistently reliable. Low scores were achieved in external validity and selection bias, as information on selection method, place, and time period of participants' recruitment were rarely given. Moreover, small sample size was a clear limitation of most studies. A calculation of power was done in only 3 studies. Despite these drawbacks, every study was considered appropriate for the aims of this investigation.

### Pooled Rest-Stress Changes From Single Arm Studies for Healthy Subjects

Absolute mean changes and 95% CIs of HR, SV, CO, and SET of healthy subjects are visualized in Figure 2 (details in supplemental forest plots: Figures S2–S13).

**Figure 2:**
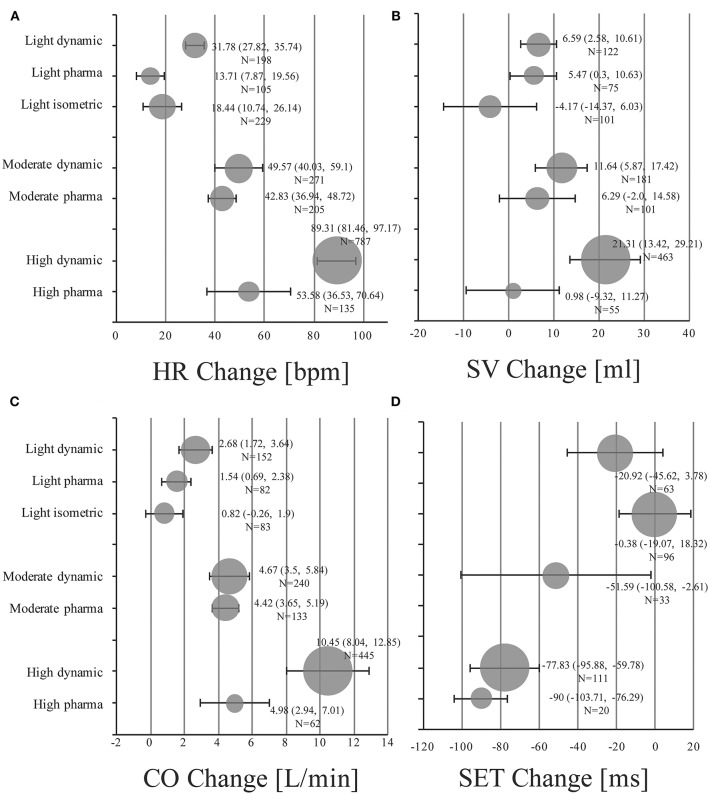
Pooled mean changes and 95% CIs in healthy individuals under stress conditions for **(A)** HR Change: absolute changes in heart rate in beats per minute (bpm). **(B)** SV Change: absolute changes in stroke volume in ml. **(C)** CO Change: absolute changes in cardiac output in liters per minute (l/min). **(D)** SET Change: absolute changes in systolic ejection time in milliseconds (ms). Bubble size is proportional to the number of stress examinations included (N).

Light dynamic exercise increased HR by 31.78 bpm (95% CI, 27.82 to 35.74; *I*^2^ = 75.2%), SV by 6.59 ml (95% CI, 2.58 to 10.61; *I*^2^ = 40.1%) and CO by 2.68 l/min (95% CI, 1.72 to 3.64; *I*^2^ = 90%) compared to resting baseline values. SET of the left ventricle was shortened by −20.92 ms (95% CI, −45.62 to 3.78; *I*^2^ = 80.5%). Low dose infusion of dobutamine (5–10 μg/kg/min) resulted in changes of 13.71 bpm (95% CI, 7.87 to 19.56; *I*^2^ = 88.6%), 5.47 ml (95% CI, 0.3 to 10.63; *I*^2^ = 0.0%), 1.54 l/min (95% CI, 0.69 to 2.38; *I*^2^ = 79.4%) for HR, SV, and CO, respectively (no studies available for changes in SET). Pooled changes of isometric exercise were 18.44 bpm (95% CI, 10.74 to 26.14; *I*^2^ = 95.6%), −4.17 ml (95% CI, −14.37 to 6.03; *I*^2^ = 81.7%), 0.82 l/min (95% CI, −0.26 to 1.9; *I*^2^ = 89.2%), and −0.38 ms (95% CI, −19.07 to 18.32; *I*^2^ = 89.4) for HR, SV, CO, and SET, respectively.

In the moderate intensity group pooled estimates of changes in HR were 49.57 bpm (95% CI, 40.03 to 59.1; *I*^2^ = 97.0%), in SV were 11.64 ml (95% CI, 5.87 to 17.42; *I*^2^ = 75.9%), and changes in CO were 4.67 l/min (95% CI, 3.5 to 5.84; *I*^2^ = 95.3%) for dynamic exercise. For moderate dosage of dobutamine (11–20 μg/kg/min) changes in HR were 42.83 bpm (95% CI, 36.94 to 48.72; *I*^2^ = 94.5%), in SV were 6.29 ml (95% CI, −2.0 to 14.58; *I*^2^ = 60.0%), and in CO were 4.42 l/min (95% CI, 3.65 to 5.19; *I*^2^ = 80.9%). SET decreased by −51.59 ms (95% CI, −100.58 to −2.61; *I*^2^ = 88.9%) in the moderate dynamic exercise group.

High dynamic exercise resulted in a mean increase of 89.31 bpm (95% CI, 81.46 to 97.17; *I*^2^ = 97.6%) in HR, 21.31 ml (95% CI, 13.42 to 29.21; *I*^2^ = 91.1%) in SV, 10.45 l/min (95% CI, 8.04 to 12.85; *I*^2^ = 98.9%) in CO and a decrease of −77.83 ms (95% CI, −95.88 to −59.78; *I*^2^ = 85.6%) in SET. For high dosage of dobutamine (21–40 μg/kg/min) HR increased by 53.58 bpm (95% CI, 36.53 to 70.64; *I*^2^ = 98.4%), SV by 0.98 ml (95% CI, −9.32 to 11.27; *I*^2^ = 52.5%), CO by 4.98 l/min (95% CI, 2.94 to 7.01; *I*^2^ = 90.4%), and SET decreased by −90.0 ms (95% CI, −103.71 to −76.29; only reported in one study).

### Heterogeneity of Results and Relationship With Intensity and Age

**Figure 3:**
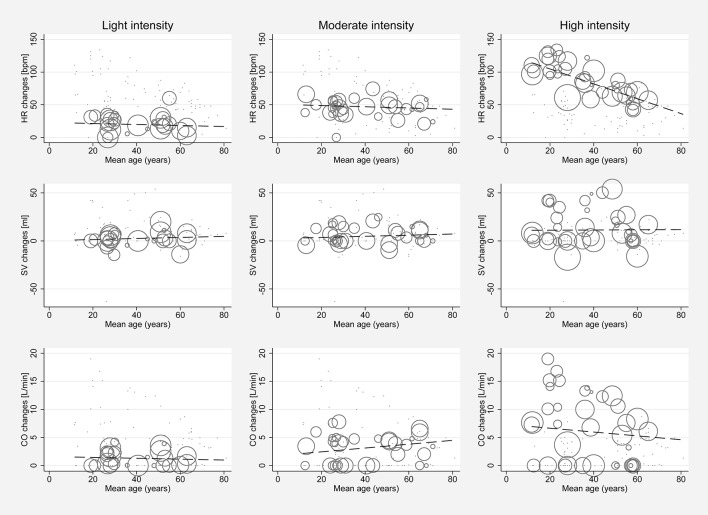
Univariate meta-regression analyses investigating age dependency of absolute mean changes of HR, SV, and CO in healthy subjects for all stress intensities. First row: age dependency of HR changes for light intensity (coefficient −0.078, *p* = 0.685, adjusted *R*^2^ = 0%), moderate intensity (coefficient −0.095, *p* = 0.534, adjusted *R*^2^ = 0%) and high intensity (coefficient −1.139, *p* < 0.001, adjusted *R*^2^ = 72.14%). Second row: Age dependency of SV changes for light intensity (coefficient 0.055, *p* = 0.767, adjusted *R*^2^ = 0%), moderate intensity (coefficient 0.059, *p* = 0.68, adjusted *R*^2^ = 0%) and high intensity (coefficient 0.01, *p* = 0.964, adjusted *R*^2^ = −7.81%). Third row: Age dependency of CO changes for light intensity (coefficient −0.007, *p* = 0.967, adjusted *R*^2^ = 0%), moderate intensity (coefficient 0.034, *p* = 0.813, adjusted *R*^2^ = 0%), and high intensity (coefficient −0.034, *p* = 0.849, adjusted *R*^2^ = 0%). Bubble size indicates study sample size.

**Figure 4:**
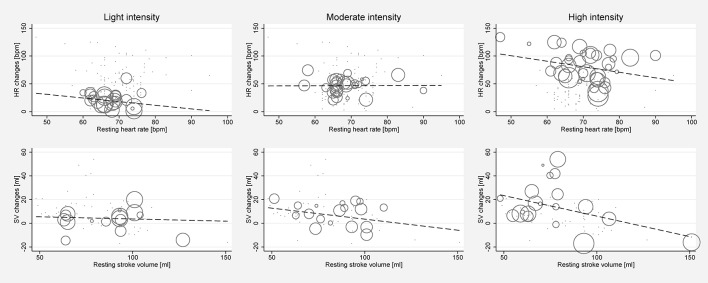
Univariate meta-regression analyses investigating the dependency of absolute mean changes of HR and SV of mean resting HR and mean resting SV in healthy subjects for all stress intensities. First row: Correlation of mean resting HR and HR changes for light intensity (coefficient −0.653, *p* = 0.341, adjusted *R*^2^ = 0%), moderate intensity (coefficient 0.022, *p* = 0.963, adjusted *R*^2^ = 0%) and high intensity (coefficient −0.995, *p* = 0.143, adjusted *R*^2^ = 3.45%). Second row: Correlation of mean resting SV and SV changes for light intensity (coefficient −0.0367, *p* = 0.839, adjusted *R*^2^ = 0%), moderate intensity (coefficient −0.187, *p* = 0.36, adjusted *R*^2^ = 0%), and high intensity (coefficient −0.346, *p* = 0.096, adjusted *R*^2^ = 25.58%). Bubble size indicates study sample size.

Significant between-study heterogeneity was found in all meta-analyses for changes in HR, SV, CO, and SET in healthy individuals (Smallest *I*^2^ = 65.7%; *p* < 0.01), except for SV analyses in the light dynamic, light pharmacological, and high pharmacological group. A visual representation of heterogeneity is available in supplemental forest plots S2–S13. Multivariate meta-regression showed that intensity level was statistically significantly associated with changes in HR (*p* < 0.001). Study arms with older participants had lower changes in HR (*p* < 0.05). None of the other covariates tested (intervention type, athlete, or health status) showed a significant association with changes in HR (*p* = 0.14; *p* = 0.13; *p* = 0.22, respectively). Multivariate estimates of systematic association of covariates with changes in HR are available in supplemental Table S7. Univariate meta-regression at different intensity levels showed a significant relationship between age and HR changes in high intensity stress testing, where older age was associated with lower changes in HR (*p* < 0.001; *R*^2^ = 72.14%). Under high intensity, the heart rate change decrease was 1.14 bpm (95% CI, 0.708 to 1.571) for each year of life. No significant age-dependency of HR changes was found in light and moderate intensity stress groups (*p* = 0.685 and *p* = 0.534, respectively). No significant correlation of age and changes in SV or CO was observed at any intensity level (*p* > 0.05). Corresponding meta-regression plots are presented in Figure 3. Four studies included children below the age of 18 years, starting from school age. Univariate meta-regression showed no significant correlation of mean resting HR and corresponding HR changes throughout all intensity levels (*p* > 0.05). No significant correlation of mean resting SV and SV changes was found (*p* > 0.05) (Figure 4).

### Comparative Studies

Only 3 studies directly compared pharmacological to dynamic stress testing (51–53). Each of these studies showed lower increases in HR, SV, and CO for dobutamine stress. Compared to dynamic stress testing, pharmacological stress showed lower mean change of HR (21.29 bpm, 95% CI −39.53 to −3.04), lower change of SV (15 ml, 95% CI −28.46 to −1.54; reported in one study) and lower change of CO (5.22 l/min, 95% CI −9.63 to −0.81). Two studies with 3 cohorts reported differences between isometric and dynamic exercise (54, 55). Compared to dynamic exercise, isometric exercise showed lower mean change of HR (35.94 bpm, 95% CI −64.31 to −7.57), higher change of SV (2 ml, 95% CI −53.31 to 57.31), lower change of CO (8.20 l/min, 95% CI −12.85 to −3.55) and higher change of SET (30.56 ms, 95% CI −36.07 to 97.16). Corresponding forest plots are available in the supplemental material (Figures S19–S25).

### Aortic Stenosis

Overall pooled mean changes and 95% CIs in HR, SV, CO, and SET of patients with AS can be found in Table S6 in the data supplement and in the corresponding forest plots (Figures S14–S18).

Light pharmacological stress caused changes in HR of 14 bpm (95% CI 9.82 to 18.18; *I*^2^ = 0%), in SV of 8 ml (95% CI 3.82 to 12.18; *I*^2^ = 0%), of CO in 1.33 l/min (95% CI 1.11 to 1.55; *I*^2^ = 0%), and in SET of −41 ms (95% CI −52.29 to −29.71; *I*^2^ = 0%). Isometric stress caused changes in HR of 5 bpm (95% CI −1.17 to 11.17; *I*^2^ = 0%), in SV of −4 ml (95% CI −16.43 to 8.43; *I*^2^ = 0%), and in CO of 0.21 l/min (95% CI −0.64 to 1.06; *I*^2^ = 0%).

During moderate dynamic stress values of pooled HR changes were 46.45 bpm (95% CI 42.63 to 50.27; *I*^2^ = 40.6%). During moderate pharmacological stress changes in HR were 18.66 bpm (95% CI 2.38 to 34.93; *I*^2^ = 89.2%). During moderate pharmacological stress changes in SV were 13.11 ml (95% CI 7.99 to 18.23; *I*^2^ = 0%), in CO 1.7 l/min (95% CI 0.74 to 2.66) and in SET −40.0 ms (95% CI −45.33 to −34.67). After excluding the two studies with low-flow, low-gradient AS with consecutive LV-EF impairment from analysis of the moderate pharmacological stress level, the changes during moderate pharmacological stress in HR were 18.0 bpm (95% CI 0.47 to 35.53; *I*^2^ = 0%) and in SV 9.0 ml (95% CI −2.69 to 20.69; *I*^2^ = 0%). In patients with low-flow, low-gradient AS with LV-EF impairment pooled HR changes during moderate pharmacological stress were 18.92 bpm (95% CI −2.64 to 40.48; *I*^2^ = 94.6%) and pooled SV changes were 14.09 ml (95% CI 8.39 to 19.79; *I*^2^ = 0%).

Changes during high intensity dynamic stress were HR 55.32 bpm (95% CI 47.31 to 63.33; *I*^2^ = 91.9%), SV −0.96 ml (95% CI −5.27 to 3.35; *I*^2^ = 17.1%), CO 5.3 l/min (95% CI 3.46 to 7.14; *I*^2^ = 84.2%), and SET −58.94ms (95% CI −127.52 to 9.63; *I*^2^ = 92.5%). During high pharmacological stress changes were HR 42.52 bpm (95% CI 32.77 to 52.28; *I*^2^ = 59.1%), SV 14.06 ml (95% CI −1.62 to 29.74; *I*^2^ = 85.2%), and CO 3.92 l/min (95% CI 2.45 to 5.39; *I*^2^ = 78.5%).

Insufficient data were available to conduct extensive subgroup analyses for intensity levels in patients with AS. Data of the light intensity group of pharmacological and isometric stress were derived from only a single study, respectively, and no data at all were found for patients with AS undergoing light dynamic exercise. In the moderate dynamic exercise group only changes in HR were available; in the corresponding pharmacological stress group changes in HR and SV were estimated based on three studies, while CO and SET changes are based on a single study. High intensity level changes are based on data of 10 studies. In contrast to findings in healthy subjects, SV decreased in AS under maximal dynamic exercise, but increased for high levels of dobutamine infusion (>20 μg/kg/min).

### Aortic Coarctation

Only four studies in patients with surgically repaired CoA could be found, but none was identified in patients with native CoA. None of the screened studies met all of the pre-specified inclusion criteria as studies (a) did not report the parameters of interest, and (b) used other types of stress tests as well as (c) different imaging modalities. Findings are therefore limited to few single studies of interest: Pedersen et al. found an increase of 1.5 l/min/m^2^ (1 to 1.7) and 2.4 l/min/m^2^ (2 to 2.9) (median and ranges) in cardiac index for CoA patients after ascending-to-descending aortic bypass surgery, performing ergometer exercise with 0.5 and 1 W/kg, respectively, and reported no difference to healthy controls (56). Kimball et al. reported an increase of 70.8 bpm (95% CI 59.89 to 81.71) in HR and 14.3% (95% CI 7.87 to 20.73) in ejection fraction induced by maximal exercise in repaired CoA (57). Carpenter et al. found an increase in HR of 63 bpm (95% CI 53.87 to 72.13) and stated that patients with CoA show hyper-dynamic left ventricular function compared to healthy controls (58). Only one study so far investigated dobutamine stress echocardiography in patients with aortic coarctation, but focused on pressure gradients and did not report pre-defined hemodynamic parameters (30). Weber et al. used intravenous isoproterenol infusion (0.05–0.1 μg/kg/min) as pharmacological stress test instead of dobutamine resulting in a mean increase of 65 bpm (95% CI 49.37 to 80.63) in HR, 3.36 l/min (95% CI 0.48 to 6.24) in CO, and 22 mmHg (95% CI 15.17 to 28.83) in pressure gradients in patients with repaired CoA, but persistent hypertension during exercise testing (59).

## Discussion

In this systematic review and meta-analysis, we present pooled data on hemodynamic changes elicited by three commonly used methods of stress testing in clinical practice. While several studies exist that evaluate hemodynamic parameters under stress conditions in healthy individuals, evidence is still limited in disease groups for which hemodynamic changes are important parameters for treatment decisions (AS and CoA). Furthermore, there is great heterogeneity of stress testing methods and patient cohorts.

In healthy individuals and AS, intensity dependent increases in HR and CO were observed as well as further decreases in SET at each intensity level. In healthy subjects, increasing intensity levels of dynamic exercise were associated with higher increases in SV; light and moderate pharmacological stress caused some increase of SV which did not differ significantly between the two intensities, while high intensity pharmacological stress was not associated with significant changes in SV from baseline. In AS, high intensity dynamic exercise caused no significant change in SV from baseline, while all levels of pharmacologic stress were associated with a further increase in SV. Isometric exercise in healthy individuals and AS was associated with a non-significant trend to decreased SV compared to resting conditions. Only little evidence was found for light and moderate intensity levels in AS, as in these patients stress testing is mainly used to assess maximal exercise capacity and unmask stress-induced symptoms (3). Characteristic trends of changes in HR, SV, CO, and SET in healthy subjects covering different types and intensity levels of stress testing are summarized in Table 4.

**Table 4:** Trends of changes in hemodynamic parameters in healthy individuals.

**Type of stress**	**HR**	**SV**	**CO**	**SET**	**Utility for diagnostics**
Light dynamic	↑↑	↑	↑	↓	Daily life activity
Light pharmacological	↑	↑	↑		Imitate exercise without patient motion
Light isometric	↑	↔	↑	↔	Ventricular adaptation to afterload
Moderate dynamic	↑*↑↑*	↑↑	↑↑	↓↓	Daily life exercise
Moderate pharmacological	↑↑	↑	↑↑		Imitate exercise without patient motion
High dynamic	↑*↑↑↑*	↑*↑↑*	↑*↑↑*	↓*↓↓*	Limits of exercise capacity
High pharmacological	↑*↑↑*	↔	↑↑	(↓*↓↓*)	Ischemia, symptoms without patient motion

*HR indicates heart rate; SV, stroke volume; CO, cardiac output; SET, systolic ejection time. ↑, increase; ↓, decrease; ↔, no change. Blanks indicate that no data were available. Higher number of arrows indicates a more marked change of the respective parameter*.

In the clinical setting a variety of stress testing set-ups are routinely used. More knowledge about comparability would enable physicians to not only overcome barriers between sites and protocols, but to bring methods closer to standardization and interchangeability. Only few studies so far have directly compared pharmacological stress and dynamic exercise. They found lower increases of all outcome parameters in pharmacological stress. The pooled mean changes from single arm studies indicate that moderate intensity dynamic and pharmacological stress result in similar increases in HR, SV and CO with similar effect sizes and overlapping 95% CIs. Compared to dynamic exercise, light intensity dobutamine stress results in a similar increase in SV, but not in HR. While high dose dobutamine stress does not cause increases in SV it does cause significant increases in HR. As described in the literature (14), these pooled values show that the inotropic effects of dobutamine wear off with increasing doses while the chronotropic reactions are preserved. While well-monitored dobutamine stress testing is known to be generally safe, a low incidence of complications including a risk for inducing cardiac arrhythmias has been reported (60). It should therefore be only used after careful consideration of clinical needs, non-pharmacological alternatives, and under appropriate monitoring.

In direct comparison of isometric and dynamic exercise, lower increases in HR, and CO under isometric exercise were found while SV changes did not differ significantly. SET changes were higher under isometric exercise, as SET tends to decrease during dynamic exercise. Results of pooled single arm studies confirmed that HR and CO increases were lower during isometric exercise, while SV even decreased. The SET did not change significantly during isometric exercise in contrast to a decrease of the SET during light dynamic exercise. A possible explanation for this may be that isometric exercise causes a rise in total systemic vascular resistance, provoking higher mean arterial pressure, and thereby imposing an elevated afterload on the ventricle. This results in an increase in stroke work without provoking an absolute increase in SV (21). The elevated afterload is also a potential explanation for why SET does not decrease in isometric exercise (in contrast to dynamic exercise), as no acceleration of ventricular contraction occurs when a higher resistance has to be overcome.

In addition to the main differences between the three intensity levels, smaller differences between the various types of stress were also found. Meaningful differences in HR, SV, and CO changes were observed between light intensity and high intensity levels. The findings may indicate that stress tests lack comparability at the respective intensity levels, and that equivalent use in diagnostic settings could be misleading. Therefore, these results can be of clinical value in order to plan an adequate type of stress testing in an individual patient. On the other hand, changes of hemodynamic parameters within one single stress level may provide clusters showing to which degree changes in HR, SV, CO, or SET correlate with light, moderate, and high intensity stress.

Loimaala et al. previously compared the effects of ergometric, pharmacological, and isometric stress echocardiography in patients with myocardial ischemia and showed that bicycle and dobutamine tests are both accurate in the diagnostic process of coronary artery disease, although resulting in different absolute hemodynamic changes, but isometric stress was not feasible (61). Our data are in line with this study showing that hemodynamic changes of dobutamine stress and dynamic exercise show similarities, especially in the moderate intensity levels. Pooled hemodynamic effects of isometric exercise were different from those of dynamic exercise affirming that this imposes a different type of burden on the cardiovascular system (54).

With increasing age, the absolute changes in HR were lower in the high intensity level. These findings are in line with the previously described and commonly applied formula to calculate the maximal heart rate of subjects considering their age (*HR*_*max*_ = *220–age in years*) (36).

The majority of patients in the AS group had asymptomatic AS under resting conditions or, in a minority of cases, low-flow, low-gradient AS, as these conditions represent guideline indications for stress testing in AS (13). Only a few of the stress tests included were obtained in symptomatic patients with AS, all of which were conducted during invasive treatment procedures. Neither the presence of symptoms nor the low-flow, low-gradient AS differed from the overall cohort. Nevertheless, we report data for low-flow, low gradient AS with impaired ejection fraction separately, as this patient group represents a relevant clinical subgroup with altered ventricular mechanics. Patients with AS generally showed lower increases of HR and CO during stress testing than healthy persons and lower changes of SV during high intensity dynamic exercise. In addition to the stenotic valve, patients with AS were older and, due to their disease, could not reach the high stress intensities or maximal HR of healthy individuals. Additionally, AS impacts the myocardium and leads to maladaptive cardiac remodeling with left ventricular hypertrophy that can evolve into heart failure (62). Consequently, cardiac inotropy in AS is reduced which may contribute to impaired CO from the left ventricle. Surprisingly, our pooled results showed that high dose pharmacological stress induced relatively strong increases in SV in contrast to high intensity dynamic exercise in AS. As only two studies were analyzed in the high intensity pharmacological group, these particular findings remain questionable due to a paucity of evidence.

While congenital heart disease only affects ~1–2% of the overall population (63), the numbers of grown-ups with congenital heart disease are increasing and the availability of reliable exercise-testing data for this group would be highly beneficial as several approaches for non-invasive diagnostic and treatment planning already exist (64, 65). Evidence in patients with CoA is still limited to very few case series in which hemodynamic changes cannot be properly compared to pooled estimates from healthy subjects due to differences in measurement variables and units as well as considerably younger age of the patients investigated. As reflected in clinical routine most patients with CoA had been previously treated at the time of stress testing. Moreover, in CoA evidence regarding HR changes was mainly limited to three case series and only insufficient evidence was available regarding SV, CO, and SET changes. Increases in HR were slightly lower for patients with CoA undergoing high intensity dynamic exercise. One study reported higher changes of HR and lower changes of CO for high intensity pharmacological testing compared to the pooled estimates in healthy individuals but using isoproterenol instead of dobutamine as pharmacological agent. Qualitatively in one study the authors concluded that there were no differences in exercise reactions in patients with CoA compared to healthy controls, whereas in the other three studies significant differences were found. CoA is known to provoke altered baroreceptor sensitivity (66), so that different hemodynamic adaptions to stress are very likely, but accurate quantification of these differences remains impossible due to the limited availability of data.

### Limitations

Available evidence is mainly restricted to observational trials as only a few studies were available directly comparing different stress types. The results of the comparative studies seem to be in line with findings of single arm studies. However, due to the higher cumulative number of subjects in single arm studies, more detailed differentiations can be made considering intensity levels. As these studies were not primarily intended for such comparison, heterogeneity within the same group of stress type and intensity was high, which represents the main limitation of this study. Various factors could have contributed to high between-study heterogeneity. Differences in the baseline characteristics of each individual cohort, including gender differences and individual fitness levels, were difficult to assess, as these findings were not consistently reported. However, we investigated the influence of the subjects' age and ratio of athletes through various meta-regression analyses and found a significant association only for mean age in the high intensity group, but not for the ratio of athletes in the study. Nevertheless, these results should be interpreted with caution, as meta-regression analyses only reflect mean values on a study-level instead of individual patient-specific variations.

Although consensus guidelines on stress echocardiography (9, 13) and exercise testing (7) exist, consistent recommendations for recent approaches, such as MRI compatible exercise testing, are missing and large diversity of stress protocols exists within the analyzed studies. Relevant differences exist in the type of exercise applied (treadmill vs. bicycle; handgrip vs. 2-hand-bar dynamometer), the increment of workload and the end-points determined by the investigators. It can be assumed that this reflects current clinical practice across different centers, limiting comparability. Furthermore, the posture of the subjects during stress testing (supine during pharmacological stress and ergometric exercise, semi-supine during ergometric exercise or upright during isometric, ergometric, and treadmill exercise) is also known as an influencing factor of hemodynamics especially for submaximal levels of exercise (67, 68) and potentially adds further heterogeneity to the data.

To overcome these hurdles, the importance of integrative databases for cardiorespiratory stress testing like the US fitness registry FRIEND has been highlighted. Kaminsky et al. recently outlined the importance of new reference values, which can help to improve the understanding of individual cardiorespiratory fitness levels (69).

There were only a few studies reporting the pre-specified parameters of interest for AS and even fewer for CoA. The AS group included symptomatic and asymptomatic patients with varying levels of AS, which may have contributed to between-study heterogeneity. In particular studies with low-flow, low-gradient situations, and LV-EF impairment (70, 71) were analyzed separately as LV mechanics can impact circulatory parameters. Nevertheless, the hemodynamic changes of HR and SV between AS patients with and without low-flow, low-gradient situations did not differ significantly. One study with paradoxical low-gradient AS was not analyzed separately as LV function was not impaired in this study (72). In CoA only very limited evidence (4 studies) has been identified with (a) varying types of exercise testing (b) with one study using isoproterenol instead of dobutamine, and (c) without any data on isometric exercise. Future research is still needed in both disease groups in order to characterize the hemodynamic stress response. When assessing hemodynamic changes in patients with AS or CoA, regular intake of medication can be of importance. Unfortunately, an analysis of the influence of this factor was not possible due to inconsistent reporting of treatment (e.g., beta-blocker use) across studies.

Our attempt to classify stress intensity according to pre-defined absolute parameters was challenging as study end points varied greatly. While some focused on quantitative end points such as watts or METs, other studies used levels of exhaustion. METs have been subject to controversy discussions in the past due to the finding of possibly overestimating resting oxygen consumption (73), but they are still considered a useful tool to quantify individual stress levels in clinical routine (74). Another common criterion used by various investigators to classify stress intensity was the percentage of age-predicted maximal heart rate reached (HR_max_), although it is known to be subject to high individual variability (75) and results from big population studies indicate that different approaches should be used in future investigations (76).

## Conclusion and Outlook

This meta-analysis describes the changes of HR, SV, CO and SET induced by different stress types and intensity levels. Despite limited direct comparability between studies, age-dependent values are presented and can already provide reference data of normal reactions in healthy individuals and can be of use when comparing outcomes of commonly performed stress tests. Furthermore, gaps in the available evidence in both disease groups, in particular in CoA, are clarified.

Disease-specific changes may also contribute to more detailed patient-specific models and a better understanding in valvular and congenital heart disease. Data may allow estimation of the expected individual range of a circulatory response and may thus contribute to future study planning and future individualized diagnostic models, even when stress testing is contraindicated.

## Author Contributions

KR, MS-K, and MK conceptualized and designed the study. KR and MK conducted the study selection. CS, KR, and MK performed the quality assessment. KR extracted the data. KR, KB, MS-K, LG, and MK conducted statistical analysis. KR and MK drafted the manuscript. KB, MS-K, CS, LG, SS, SK, FB, and TK revised the manuscript. All authors interpreted the data and approved the final version of the manuscript.

### Conflict of Interest Statement

The authors declare that the research was conducted in the absence of any commercial or financial relationships that could be construed as a potential conflict of interest.
