# Supplementary material for: Hemodynamic Changes During Physiological and Pharmacological Stress Testing in Healthy Subjects, Aortic Stenosis and Aortic Coarctation Patients–A Systematic Review and Meta-Analysis
Source: Front Cardiovasc Med. 2019 Apr 10;6:43. doi: 10.3389/fcvm.2019.00043 (PMC6467940; doi:10.3389/fcvm.2019.00043)
Supplement: Supplementary file 1 [file Data_Sheet_1.pdf]

Supplementary Material

Hemodynamic Changes During Physiological and Pharmacological Stress Testing in Healthy Subjects, Aortic Stenosis and Aortic Coarctation Patients – A Systematic Review and Meta-Analysis

Kilian Runte[1,2], Kay Brosien[1], Maximilian Salcher-Konrad[3,4], Charlotte Schubert[1,2], Leonid Goubergrits[1], Sebastian Kelle[5,6,7], Stephan Schubert[2,7], Felix Berger[2,7], Titus Kuehne[1,2,7], Marcus Kelm[1,2*]

[1] Institute for Imaging Science and Computational Modelling in Cardiovascular Medicine, Charité – Universitätsmedizin Berlin, Berlin, Germany

[2] Department of Congenital Heart Disease, German Heart Center Berlin, Berlin, Germany

[3] Personal Social Services Research Unit, London School of Economics and Political Science, London, UK

[4] LSE Health, London School of Economics and Political Science, London, UK

[5] Department of Internal Medicine / Cardiology, German Heart Center Berlin, Berlin, Germany

[6] Department of Internal Medicine / Cardiology, Charité – Universitätsmedizin Berlin, Berlin, Germany

[7] DZHK (German Center for Cardiovascular Research), Partner Site Berlin, Berlin, Germany

*** Correspondence:**

Dr. Marcus Kelm

mkelm@dhzb.de

Supplementary Tables

Table S1. Search terms used for database search.

PubMed, 05 November 2017			N
1	a)	MRI/Magnetic resonance / "Magnetic resonance imaging"[Mesh]	709772
	b)	Echocardiography / "Echocardiography"[Mesh]	157496
2	a)	Exercise / "Exercise"[Mesh] / "Exercise Test"[Mesh]	357727
	b)	Dobutamine*/ "Dobutamine"[Mesh]	9193
	c)	Handgrip / Isometric exercise / "Isometric Contraction"[Mesh]	369960
3	a)	Healthy subjects / Normal subjects / Control subjects / Volunteers / Controls / Normals / "Healthy Volunteers"[Mesh]	1271554
	b)	Coarctation / "Aortic Coarctation"[Mesh]	11367
	c)	Aortic stenosis / "Aortic Valve Stenosis"[Mesh]	49594
4	a)	Flow / Blood flow / Aortic flow / Peak flow / "Blood Flow Velocity"[Mesh] / "Regional Blood Flow"[Mesh]	722612
	b)	“Stroke volume” / ”Stroke index”/ "Stroke volume"[Mesh]	45516
	c)	“Cardiac output” / “Cardiac index” / ”Cardiac output”[Mesh]	102882
	d)	“Time to peak” / ”Systolic time”/ ”Ejection time”	10491
5	(1a OR 1b) AND (2a OR 2b OR 2c) AND (3a OR 3b OR 3c) AND (4a OR 4b OR 4c OR 4d)		1616
6	Limit to: Humans; English, German; Publication after 1985/01/01		1372
7	Limit to: Full text availability		**1188**

Table S2. Characteristics of the included studies for healthy subjects.

Author	Publication	Location	Subjects	Mean age (yrs)	Special charac-teristics	Type of Stress Test	Intensity of Stress	Imaging Modality	Relevant Outcomes reported	Comments	Quality
Barber (2016)	Magnetic Resonance-Augmented Cardiopulmonary Exercise Testing: Comprehensively Assessing Exercise Intolerance in Children With Cardiovascular Disease. *Circ Cardiovasc Imaging, 9*(12).	UK	10	13	children	supine ergometer	sub-maximal; 4,8 METS	MRI	HR, SVI, CI, EF	Intended high intensity not reached	16/19 high
Roberts (2015)	Real-time aortic pulse wave velocity measurement during exercise stress testing. *J Cardiovasc Magn Reson, 17*, 86.	New Zealand	50	52.6	-	supine ergometer	38 W	MRI	HR, SV, CO, EF		10/17 moderate
Forouzan (2015)	Non-invasive measurement using cardiovascular magnetic resonance of changes in pulmonary artery stiffness with exercise. J Cardiovasc Magn Reson, 17, 109.	USA	15	26.5	-	supine leg-stepping	- 45 W<br>- 35 W	MRI	HR, SV, CO	2 different stress intensities	9/17 moderate

Claessen (2015)	Pulmonary vascular and right ventricular reserve in patients with normalized resting hemodynamics after pulmonary endarterectomy. J Am Heart Assoc, 4(3), e001602.	Belgium	14	36	-	supine ergometer	142 W	MRI	HR, SV, CO, EF		14/19 high
Barber (2015)	MR augmented cardiopulmonary exercise testing-a novel approach to assessing cardiovascular function. Physiol Meas, 36(5), N85-94.	UK	15	38.6	-	supine ergometer	exhaustion	MRI	HR, SV, CO		11/17 high
Steding-Ehrenborg (2013)	Moderate intensity supine exercise causes decreased cardiac volumes and increased outer volume variations: a cardiovascular magnetic resonance study. *J Cardiovasc Magn Reson, 15*, 96.	Sweden	26	30	-	supine leg-stepping	40 bpm increase in HR	MRI	HR, SV, CO, EF		9/17 moderate
Weber (2011)	High-resolution phase-contrast MRI of aortic and pulmonary blood flow during rest and physical exercise using a MRI compatible bicycle ergometer. *Eur J Radiol, 80*(1), 103-108.	Germany	20	26.8	-	supine ergometer	- No load operation<br>- 40 % increase in HR<br>- 80% increase in HR	MRI	CO, TTP	3 different stress intensities (no load operation only in 10 subjects)	10/17 moderate

Punta-wangkoon (2009)	Reduced peripheral arterial blood flow with preserved cardiac output during submaximal bicycle exercise in elderly heart failure." J Cardiovasc Magn Reson 11:48.	USA	13	67	-	supine ergometer	sub-maximal; 37 W	MRI	HR, CO	CO given as mean aortic flow in l/min	15/19 high
Roest (March 2001)	Biventricular response to supine physical exercise in young adults assessed with ultrafast magnetic resonance imaging." Am J Cardiol 87(5): 601-605.	Nether-lands	16	17.5	-	supine ergometer	sub-maximal; 130 W	MRI	HR, SV, CO, EF		9/17 moderate
Roest (November 2001)	Prolonged cardiac recovery from exercise in asymptomatic adults late after atrial correction of transposition of the great arteries: evaluation with magnetic resonance flow mapping. *Am J Cardiol*, *88*(9), 1011-1017.	Nether-lands	12	28	-	supine ergometer	sub-maximal; 60 % of $VO2_{max}$	MRI	HR, SV, CO		12/19 moderate
Niezen (1998)	Measurement of aortic and pulmonary flow with MRI at rest and during physical exercise. *J Comput Assist Tomogr*, *22*(2), 194-201.	Nether-lands	16	30	-	supine ergometer	- 50 W<br>- 100 W	MRI	HR, SV, CO, TTP, SET	2 different stress intensities	7/17 moderate

Stephensen (2016)	Changes in blood volume shunting in patients with atrial septal defects: Assessment of heart function with cardiovascular magnetic resonance during dobutamine stress. Eur Heart J Cardiovasc Imaging. 2017;18:1145-1152.	Sweden	16	35	-	dobuta-mine ± atropine	20 µg/kg/min (70% of HR_{max})	MRI	HR, SVI, CI, EF		12/19 moderate
Pingitore (2013)	Influence of preload and afterload on stroke volume response to low-dose dobutamine stress in patients with non-ischemic heart failure: a cardiac MR study. *Int J Cardiol*, *166*(2), 475-481.	Italy	12	55	-	dobutamine	20 µg/kg/min	MRI	HR, SV, CO, EF		14/19 high
Ahtarovski (2013)	Termination of dobutamine infusion causes transient rebound left heart diastolic dysfunction in healthy elderly women but not in men: a cardiac magnetic resonance study. *Am J Physiol Heart Circ Physiol*, *305*(7), H1098-1103.	Denmark	10<br>10	65<br>65	male<br>female	dobutamine	15 µg/kg/min	MRI	HR, SVI, CI, EF	2 different cohorts: male and female	12/19 moderate

Ahtarovski (2012)	Left atrial and ventricular function during dobutamine and glycopyrrolate stress in healthy young and elderly as evaluated by cardiac magnetic resonance. *Am J Physiol Heart Circ Physiol*, *303*(12), H1469-1473.	Denmark	20<br><br>20	25<br><br>65	young<br><br>elderly	dobutamine	15 µg/kg/min	MRI	HR, SVI, CI, EF	2 different cohorts: young and elderly	13/19 moderate
Schuster (2011)	Cardiovascular magnetic resonance myocardial feature tracking detects quantitative wall motion during dobutamine stress." J Cardiovasc Magn Reson 13:58.	UK	10	40.6	-	dobutamine	- 10 µg/kg/min<br><br>- 20 µg/kg/min	MRI	HR, SVI, CI, EF	2 different stress intensities	9/17 moderate
Mandapaka (2011)	Simultaneous measurement of left and right ventricular volumes and ejection fraction during dobutamine stress cardiovascular magnetic resonance. *J Comput Assist Tomogr*, *35*(5), 614-618.	USA	13	53	-	dobutamine ± atropine	- 7.5 µg/kg/min<br><br>- 80 % of HR_{max}	MRI	HR, SV, EF	2 different stress intensities	10/17 moderate
Oosterhof (2005)	Disparity between dobutamine stress and physical exercise magnetic resonance imaging in patients with an intra-atrial correction for transposition of the great arteries. *J Cardiovasc Magn Reson*, *7*(2), 383-389.	Nether-lands	11<br><br>14	28.9<br><br>26.8	-	dobutamine<br><br>supine ergometer	- 15 µg/kg/min<br><br>- 60% of $VO2_{max}$	MRI	HR, SVI, CI, EF	2 different cohorts: dobutamine stress vs. ergometer stress	11/19 moderate

Dodge-Khatami (2002)	Comparable systemic ventricular function in healthy adults and patients with unoperated congenitally corrected transposition using MRI dobutamine stress testing." Ann Thorac Surg 73(6): 1759-1764.	Nether-lands	11	31	-	dobutamine	15 µg/kg/min	MRI	HR, SVI, CI, EF		10/19 moderate
Pennell (1995)	Assessment of magnetic resonance velocity mapping of global ventricular function during dobutamine infusion in coronary artery disease. *Br Heart J, 74*(2), 163-170.	UK	10	51	-	Dobutamine	- 5 µg/kg/min<br>- 10 µg/kg/min<br>- 15 µg/kg/min<br>- 20 µg/kg/min	MRI	HR, SV, CO	4 different stress intensities	12/19 moderate
van Rugge (1993)	Quantitation of global and regional left ventricular function by cine magnetic resonance imaging during dobutamine stress in normal human subjects. *Eur Heart J, 14*(4), 456-463.	Nether-lands	23	25	-	dobutamine	15 µg/kg/min	MRI	HR, SV, CO, EF		10/17 moderate
Knobels-dorff-Brenken-hoff (2013)	Isometric handgrip exercise during cardiovascular magnetic resonance imaging: set-up and cardiovascular effects. *J Magn Reson Imaging, 37*(6), 1342-1350.	Germany	53	45	-	handgrip	30 % of maximal voluntary contraction (MVC)	MRI	HR, SV, CO		10/17 moderate

D'Alto (2017)	Echocardiographic assessment of right ventricular contractile reserve in healthy subjects. Echocardiography 34(1): 61-68.	Belgium, Italy	90	39	-	semisupine ergometer	177 W	Echo	HR, SV, CO		10/17 moderate
D'Andrea (2016)	Right atrial morphology and function in patients with systemic sclerosis compared to healthy controls: a two-dimensional strain study. Clin Rheumatol, 35(7), 1733-1742.	Italy	55	50.6	-	supine ergometer	144 W	Echo	HR, SV		14/19 high
Wang (2014)	Changes of ventricular and peripheral performance in patients with heart failure and normal ejection fraction: insights from ergometry stress echocardiography. Eur J Heart Fail, 16(8), 888-897.	Hong Kong	50	56	-	semisupine ergometer	85% of HR_{max}; 89 W	Echo	HR, SVI, CI		13/19 moderate
Khouri (2014)	Utility of 3-dimensional echocardiography, global longitudinal strain, and exercise stress echocardiogra-phy to detect cardiac dysfun-ction in breast cancer patients treated with doxorubicin-containing adjuvant therapy. Breast Cancer Res Treat, 143(3),531-539.	Denmark	20	57	female	upright treadmill	exhaustion; 8.7 METS	Echo	HR, SV, CI, EF		15/19 high

Tan (2013)	Exercise-induced torsional dyssynchrony relates to impaired functional capacity in patients with heart failure and normal ejection fraction. Heart, 99(4), 259-266.	UK	38	71	-	semisupine ergometer	HR up to 100 bpm	Echo	HR, SVI, CO		14/19 high
Klasnja (2013)	Cardiac power output and its response to exercise in athletes and non-athletes. Clin Physiol Funct Imaging, 33(3), 201-205.	UK	32<br>20	20<br>19.8	un-trained<br>trained	upright ergometer	177 W<br>205 W	Echo	HR, SV, CO	2 different cohorts: untrained vs. trained	11/19 moderate
Henein (2013)	Impaired left ventricular systolic function reserve limits cardiac output and exercise capacity in HFpEF patients due to systemic hypertension. Int J Cardiol, 168(2), 1088-1093.	Sweden	14	65	-	semisupine ergometer	exhaustion	Echo	HR, SV, CO, EF		15/19 high
Lee (2012)	Exercise with a twist: left ventricular twist and recoil in healthy young and middle-aged men, and middle-aged endurance-trained men. J Am Soc Echocardiogr, 25(9), 986-993.	Canada	11<br>12	24<br>53.8	young un-trained<br>middle aged trained	supine ergometer	HR up to 105 bpm	Echo	HR, EF	2 different cohorts: young untrained vs. middle aged trained	11/19 high
La Gerche (2012)	Maximal oxygen consumption is best predicted by measures of cardiac size rather than function in healthy adults. Eur J Appl Physiol, 112(6), 2139-2147.	Australia	55	37	-	semisupine ergometer	exhaustion	Echo	HR, EF		10/17 moderate

Cheung (2012)	Dynamic dyssynchrony and impaired contractile reserve of the left ventricle in beta-thalassaemia major: an exercise echocardiographic study. PLoS One, 7(9), e45265.	Hong Kong	17	25,3	-	supine ergometer	70 % of HR_{max}	Echo	HR, SVI, CI, EF		14/19 high
Donal (January 2011)	Impact of aortic stenosis on longitudinal myocardial deformation during exercise. Eur J Echocardiogr, 12(3), 235-241.	France	43	68	-	semisupine ergometer	92 W	Echo	HR, EF	Study also included in AS group	15/17 high
Donal (May 2011)	Comparison of the heart function adaptation in trained and sedentary men after 50 and before 35 years of age. Am J Cardiol, 108(7), 1029-1037.	France				semisupine ergometer	sub-maximal	Echo	SV, CO, EF	4 different cohorts: elderly trained vs. untrained vs. young trained vs. untrained	13/19 high
			38	61.5	elderly trained		110 W				
			18	25.2	young trained		133 W				
			15	58.9	elderly un-trained		90 W				
			27	26.2	young un-trained		103 W				

Bombardini (2011)	Abnormal shortened diastolic time length at increasing heart rates in patients with abnormal exercise-induced increase in pulmonary artery pressure. Cardiovasc Ultrasound, 9, 36.	Italy	16	35	-	semisupine ergometer	exhaustion	Echo	HR, CI, EF		14/19 high
Dini (2010)	Peak power output to left ventricular mass: an index to predict ventricular pumping performance and morbidity in advanced heart failure. J Am Soc Echocardiogr, 23(12), 1259-1265.	Italy	15	55	-	semisupine ergometer	150 W	Echo	HR, SV, CO, EF		14/19 high
Argiento (2010)	Exercise stress echocardiography for the study of the pulmonary circulation. Eur Respir J, 35(6), 1273-1278.	Belgium	25	36	-	semisupine ergometer	170 W	Echo	HR, CO		9/17 moderate
Schuster (2009)	Cardiac function during exercise in obese prepubertal boys: effect of degree of obesity. Obesity (Silver Spring), 17(10), 1878-1883.	France	17	11.6	children	semisupine ergometer	exhaustion; 95 W	Echo	HR, SV, CO		14/19 high
Goodman (2009)	Left ventricular contractile function is preserved during prolonged exercise in middle-aged men. J Appl Physiol (1985), 106(2), 494-499.	Canada	12	43.5	-	semisupine ergometer	65 % of $VO2_{max}$	Echo	HR, SV, EF		10/17 moderate

Cotrim (2008)	Do healthy individuals develop intraventricular gradients during exertion? Rev Port Cardiol, 27(11), 1367-1375.	Portugal	34	50	-	upright treadmill	exhaustion	Echo	HR, SV, CI		11/17 high
Bombardini (2008)	Diastolic time - frequency relation in the stress echo lab: filling timing and flow at different heart rates. Cardiovasc Ultrasound 6: 15.	Italy	64	57	controls with normal stress echo	semisupine ergometer	exhaustion	Echo	HR, SI, CI, SET		11/17 high
De Souza (2007)	A stress echocardiography study of cardiac function during progressive exercise in pediatric oncology patients treated with anthracyclines. Pediatr Blood Cancer 49(1): 56-64.	Canada	12	11.9	children	semisupine ergometer	exhaustion	Echo	HR, SVI, CI, SET		12/19 moderate
Rowland (2005)	Effect of pectus excavatum deformity on cardiorespiratory fitness in adolescent boys. Arch Pediatr Adolesc Med 159(11): 1069-1073.	USA	20	12.5	children	upright ergometer	- 50 W<br><br>- 146 W	Echo	HR, SVI, CI	2 different intensities	12/19 moderate
Sagiv (2000)	Left ventricular contractility and function at peak aerobic and anaerobic exercises. Med Sci Sports Exerc 32(7): 1197-1201.	Israel	22	19	trained	upright ergometer	- 255 W; aerobic<br>- 457 W; anaerobic	Echo	CO, EF	2 different intensities	12/17 high

Auerbach (1999)	Attenuated responses of Doppler-derived hemodynamic parameters during supine bicycle exercise in heart transplant recipients. Cardiology 92(3): 204-209.	Israel	18	51.1	-	supine ergometer	85 % of HR_{max}	Echo	HR, SV, CO, SET, TTP		11/19 moderate
Fisman (1990)	Altered left ventricular volume and ejection fraction responses to supine dynamic exercise in athletes. J Am Coll Cardiol 15(3): 582-588.	Israel	22<br>22	24.3<br>23.1	- un-trained<br>- trained	supine ergometer	- 138 W<br>- 190 W	Echo	HR, EF	2 different cohorts: untrained vs. trained	16/19 high
Ginzton (1989)	Effect of long-term high intensity aerobic training on left ventricular volume during maximal upright exercise. J Am Coll Cardiol 14(2): 364-371.	USA	14<br>15	28<br>19	un-trained<br>trained	upright ergometer	- 25% of HR_{max}<br>- exhaustion<br>- 25% of HR_{max}<br>- exhaustion	Echo	HR, SVI, EF	2 different cohorts (untrained vs. trained) with 2 different intensities	12/19 moderate
Thompson (1987)	Comparison of ventricular volumes in normal and post-myocardial infarction subjects. Med Sci Sports Exerc 19(5): 430-435.	USA	13	48.5	-	semisupine ergometer	85 % of HR_{max}	Echo	HR, SV, CO, EF		12/19 moderate
Mehdirad (1987)	Evaluation of left ventricular function during upright exercise: correlation of exercise Doppler with postexercise two-dimensional echocardiographic results. Circulation 75(2): 413-419.	USA	12	40	-	upright treadmill	Bruce protocol stage IV	Echo	HR, SVI, CI, EF		12/19 moderate

Gardin (1986)	Studies of Doppler aortic flow velocity during supine bicycle exercise. Am J Cardiol 57(4): 327-332.	USA	17	20.5	-	supine ergometer	- 50 W<br><br>- sub-maximal<br><br>- exhaustion	Echo	HR, SET	3 different intensities	10/17 moderate
Bryg (1986)	Effect of coronary artery disease on Doppler-derived parameters of aortic flow during upright exercise. Am J Cardiol 58(1): 14-19.	USA	20	27	-	upright treadmill	- Bruce ptocol stage II<br><br>- stage IV	Echo	HR, SVI, CI	2 different intensities	11/19 moderate
Lau (2014)	Dobutamine stress echocardiography for the assessment of pressure-flow relationships of the pulmonary circulation. Chest, 146(4), 959-966.	Australia	22<br><br>22	46<br><br>44	-<br><br>-	dobutamine<br><br>semisupine ergometer	20 µg/kg/min<br><br>exhaustion	Echo	HR, CO	2 different stress types in 2 age matched cohorts: dobutamine vs. ergometer	14/19 high
Maras (2013)	Patterns of cardiac dysfunction coinciding with exertional breathlessness in hypertrophic cardiomyopathy. Int J Cardiol, 170(2), 233-238.	Sweden	17	58	-	dobutamine	85 % of HR_{max}	Echo	HR, systolic time	systolic time not convertible into SET	11/19 moderate
Brili (2007)	Dobutamine stress echocardiography for the evaluation of cardiac reserve late after Fontan operation. Hellenic J Cardiol, 48(5), 252-257.	Greece	10	28	-	dobutamine	- 30 µg/kg/min<br>- 20 µg/kg/min<br>- 10 µg/kg/min<br>- 5 µg/kg/min	Echo	HR, SV, CO, EF	4 different intensities<br><br>age matched controls	13/19 moderate

Arshad (2004)	Systole-diastole mismatch in hypertrophic cardiomyopathy is caused by stress induced left ventricular outflow tract obstruction. Am Heart J, 148(5), 903-909.	UK	23	58	-	dobutamine	40 µg/kg/min	Echo	HR, systolic time	systolic time not convertible into SET	11/19 moderate
Cnota (2003)	Cardiovascular physiology during supine cycle ergometry and dobutamine stress. *Med Sci Sports Exerc, 35*(9), 1503-1510.	USA	32	23.5	-	dobutamine<br><br>supine ergometer	85 % of HR_{max}; max. 50 µg/kg/min<br><br>85 % of HR_{max}	Echo	HR, SV, CO	2 different stress types in the same cohort: dobutamine vs. ergometer	10/17 moderate
Duncan (2001)	Long axis electromechanics during dobutamine stress in patients with coronary artery disease and left ventricular dysfunction. Heart, 86(4), 397-404.	UK	20	58	-	dobutamine	85 % of HR_{max}; max. 40 µg/kg/min	Echo	HR, SET		13/19 moderate
Marmor (1996)	Evaluation of contractile reserve by dobutamine echocardiography: noninvasive estimation of the severity of heart failure. Am Heart J, 132(6), 1195-1201.	Israel	10		atypical chest pain (no cardiac cause)	dobutamine	- 40 µg/kg/min<br>- 30 µg/kg/min<br>- 20 µg/kg/min<br>- 10 µg/kg/min<br>- 5 µg/kg/min	Echo	HR, CO	4 different intensities<br><br>no age provided	13/19 moderate
Blomstrand (1995)	Cardiovascular effects of dobutamine stress testing in healthy women. Clinical cardiology. 1995;18:659-663.	Sweden	11	63	female	dobutamine	- 10 µg/kg/min<br>- 5 µg/kg/min	Echo	HR, SV, CO	2 different intensities	11/17 high

Weiner (2012)	The impact of isometric handgrip testing on left ventricular twist mechanics. J Physiol, 590(20), 5141-5150.	USA	18	29.7	healthy controls	handgrip	40 % of MVC	Echo	HR, SV, CO, EF		11/17 high
Bozkurt (2006)	Echocardiographic findings in patients with Behcet's disease. Am J Cardiol, 97(5), 710-715.	Turkey	50	35.7	healthy controls	handgrip	50 % of MVC	Echo	HR, EF		15/19 high
Lev (1998)	Exercise-induced aortic flow parameters in early postmenopausal women and middle-aged men. J Intern Med, 243(4), 275-280.	Israel	15<br><br>15	55<br><br>52	female<br><br>male	- 2-hand bar dynamo-meter<br>- supine ergometer<br><br>- 2-hand bar dynamo-meter<br>- supine ergometer	- 50 % of MVC<br>- 50 W<br><br>- 50 % of MVC<br>- 50 W	Echo	HR, SET, TTP	2 different stress types in 2 different cohorts: isometric vs. dynamic exercise in men vs. women	12/19 moderate
Fisman (1991)	Pronounced reduction of aortic flow velocity and acceleration during heavy isometric exercise in coronary artery disease. Am J Cardiol, 68(5), 485-491.	Israel	48	48	male	2-hand bar dynamo-meter	50 % of MVC	Echo	HR, SVI, CI, SET, TTP		14/19 high
Bamrah (1991)	Static versus dynamic exercise: effects on Doppler echocardiographic indices of left ventricular performance. Clin Cardiol, 14(6), 481-488.	USA	12	60	-	- handgrip<br><br>- supine ergometer	- 30 % of MVC<br>- 160 W	Echo	HR, SV, CO	2 different stress types in the same cohort: isometric vs. dynamic exercise	10/19 moderate

Krzeminski (1989)	Effect of endurance training on cardiovascular response to static exercise performed with untrained muscles. Int J Sports Med, 10(5), 363-367.	Poland	18	20.9	-	handgrip	30 % of MVC	Echo	HR, SV, SET, EF		9/17 medium

Table S3. Characteristics of the included studies for patients with aortic stenosis.

**Author**	**Publication**	**Location**	**Number of Patients**	**Mean age (years)**	**Type of AS**	**Type of Stress Test**	**Intensity of Stress**	**Imaging Modality**	**Relevant Outcomes reported**	**Comments**	**Quality**
Pérez Del Villar (2017)	The Functional Significance of Paradoxical Low-Gradient Aortic Valve Stenosis: Hemodynamic Findings During Cardiopulmonary Exercise Testing. JACC Cardiovasc Imaging 10(1): 29-39.	Spain	20	77	paradoxical low-gradient, severe	semisupine ergometer	symptom-limited	Echo	HR, SVI, CI, SET	Aortic valve area (AVA) <1cm² and <0.6cm²/m²	9/17 moderate
Lancellotti (2012)	Determinants and prognostic significance of exercise pulmonary hypertension in asymptomatic severe aortic stenosis. Circulation, 126(7), 851-859.	Belgium	105	71	asymp-tomatic severe	semisupine ergometer	symptom-limited	Echo	HR, EF	AVA <0.6cm²/m²	16/20 high
Donal (January 2011)	Impact of aortic stenosis on longitudinal myocardial deformation during exercise. Eur J Echocardiogr, 12(3), 235-241.	France	 136<br>69	 67<br>67	asymp-tomatic, moderate to severe<br>- normal exercise test<br>- ab-normal exercise test	semisupine ergometer	 - 96 W<br>- 86 W	Echo	HR, EF	AVA<1.2 or <1cm² and <0.6cm²/m²<br>2 different cohorts: normal and abnormal exercise test<br>Study also included in healthy group	15/17 moderate

Marechaux (2010)	Usefulness of exercise-stress echocardiography for risk stratification of true asymptomatic patients with aortic valve stenosis. Eur Heart J, 31(11), 1390-1397.	Canada	135	64	asymp-tomatic, mode-rate	semisupine ergometer	symptom-limited; 90 W	Echo	HR, SV, EF	AVA <1.5cm² or <0.9cm²/m²	13/18 high
Lancellotti (2008)	Determinants of an abnormal response to exercise in patients with asymptomatic valvular aortic stenosis. Eur J Echocardiogr, 9(3), 338-343.	Belgium	 <br><br><br>68<br><br><br>60	 <br><br><br>69<br><br><br>69	asymp-tomatic, severe<br><br>- normal exercise test<br>- ab-normal exercise test	semisupine ergometer	symptom-limited	Echo	HR, EF	AVA <1cm²<br><br><br><br>2 different cohorts: normal and abnormal exercise test	12/17 high
Legget (1996)	Gender differences in left ventricular function at rest and with exercise in asymptomatic aortic stenosis. Am Heart J 131(1): 94-100.	USA	 <br><br><br>45<br><br>23	 <br><br><br>62<br><br>64	asymp-tomatic , mode-rate<br><br>- male<br><br>- female	upright treadmill	exhaustion	Echo	HR, SV, CO	AVA ≤1.4cm² and ≤0.7cm²/m²<br><br><br><br>2 different cohorts: male and female	14/19 high
Burwash (1994)	Flow dependence of measures of aortic stenosis severity during exercise. Journal of the American College of Cardiology, 24(5), 1342-1350.	USA	66		asymp-tomatic	upright treadmill	symptom-limited	Echo	HR, SV, CO		10717 moderate

Otto (1992)	Physiologic changes with maximal exercise in asymptomatic valvular aortic stenosis assessed by Doppler echocardiography. J Am Coll Cardiol 20(5): 1160-1167.	USA	28	61	asymp-tomatic	upright treadmill	exhaustion	Echo	HR, SV, CO, SET		11/17 high
Mahfouz (2015)	Left ventricular restrictive filling pattern and the presence of contractile reserve in patients with low-flow/low-gradient severe aortic stenosis. Echocardiography, 32(1), 65-70.	Egypt	14	51	low-flow, low-gradient, severe, contrac-tile reserve	dobutamine	11 µg/kg/min	Echo	HR, SV, EF	AVA<1cm²	13/19 moderate
Bartko (2013)	Two-dimensional strain for the assessment of left ventricular function in low flow-low gradient aortic stenosis, relationship to hemodynamics, and outcome: a substudy of the multicenter TOPAS study. Circ Cardiovasc Imaging, 6(2), 268-276.	Austria	47	73	low-flow, low-gradient, mode-rate to severe	dobutamine	20 µg/kg/min	Echo	HR, SV, EF	AVA ≤1.2cm² and ≤0.6cm²/m²	11/18 high
Takeda (1999)	The relation between transaortic pressure difference and flow during dobutamine stress echocardiography in patients with aortic stenosis. Heart, 82(1), 11-14.	UK	50	65.3	asymp-tomatic, mild to severe	dobutamine	29 µg/kg/min	Echo	HR, SV, CO		12/17 high

Lin (1998)	Dobutamine stress Doppler hemodynamics in patients with aortic stenosis: feasibility, safety, and surgical correlations. Am Heart J, 136(6), 1010-1016.	USA	27	74	severe	dobutamine	27 µg/kg/min	Echo	HR, SV, CO, EF	AVA <1cm²	16/18 high
Tardif (1997)	Simultaneous determination of aortic valve area by the Gorlin formula and by transesophageal echocardiography under different transvalvular flow conditions. Evidence that anatomic aortic valve area does not change with variations in flow in aortic stenosis. J Am Coll Cardiol, 29(6), 1296-1302.	Canada	11	63	need for aortic valve repair	dobutamine	> 15% increase in CO; 5 - 20 µg/kg/min	Transeso-phageal Echo	HR, SV, CO, SET		10/17 moderate
Casale (1992)	Effects of dobutamine on Gorlin and continuity equation valve areas and valve resistance in valvular aortic stenosis. Am J Cardiol 1992;70:1175-9.	USA	12	81	severe	dobutamine	50 % increase in CO	Echo & Cardiac Cathe-terization	HR, SV, CO, SET	AVA <1cm²	10/17 moderate
Little (2007)	Impact of blood pressure on the Doppler echocardiographic assessment of severity of aortic stenosis. Heart, 93(7), 848-855.	Canada	22	70	mild to severe	handgrip	40 % of max. voluntary contraction	Echo	HR, SV, CO		11/17 high

Table S4. Characteristics of the included studies for patients with aortic coarctation.

Author	Publication	Location	Number of Patients	Mean age (years)	State of CoA	Type of Stress Test	Intensity of Stress	Imaging Modality	Relevant Outcomes reported	Comments	Quality
Pedersen (2010)	Blood flow measured by magnetic resonance imaging at rest and exercise after surgical bypass of aortic arch obstruction. Eur J Cardiothorac Surg, 37(3), 658-661.	Denmark	7	18	bypass tube from Aorta asc. to Aorta desc.	supine ergometer	- 0.5 W/kg<br>- 1 W/kg	MRI	CI	2 different stress intensities<br>only median and ranges available	12/19 moderate
Kimball (1986)	Persistent ventricular adaptations in postoperative coarctation of the aorta. J Am Coll Cardiol, 8(1), 172-178.	Canada	25	26.1	surgical repair	supine exercise	exhaustion	Radio-nuclide Angio-graphy	HR, EF, TTP	TTP calculated	12/19 moderate
Carpenter (1985)	Left ventricular hyperkinesia at rest and during exercise in normotensive patients 2 to 27 years after coarctation repair. J Am Coll Cardiol 6(4): 879-886.	USA	32	27	surgical repair	supine ergometer	exhaustion	Echo and Angio-graphy	HR, EF	hyperdynamic left ventricle	12/19 moderate
Weber (1993)	Discrepancies in aortic growth explain aortic arch gradients during exercise. J Am Coll Cardiol, 21(4), 1002-1007.	USA	8	12	surgical repair	isopro-terenol	0.05 - 0.1 µg/kg/min	Angio-graphy	HR, CI		10/17 moderate

Table S5. Baseline characteristics for patients with aortic stenosis.

	**Dynamic exercise**		**Pharmacological stress**		**Isometric exercise**		**Kruskal-Wallis-Test**
		Study arms reporting variable (N of tests)		Study arms reporting variable (N of tests)		Study arms reporting variable (N of tests)	P Value
N of stress tests overall		11 (755)		6 (161)		1 (22)	
Age, years	67 (64-69)	10 (689)	69.2 (63-74)	6 (161)	70 (70-70)	1 (22)	0.726
Male, %	64.4 (37-66.4)	8 (561)	64.1 (41.6-66.6)	6 (161)	73	1 (22)	0.511
BSA, m^2	1.8 (1.8-1.8)	7 (533)	1.9 (1.9-1.9)	1 (47)	1.96 (1.96-1.96)	1 (22)	
BMI, kg/m^2	26.3 (26.1-27.8)	4 (223)	N/A	-	N/A	-	
Resting HR, bpm	71† (70-74)	11 (755)	75.5†, ‡ (72-76)	6 (161)	62‡ (62-62)	1 (22)	0.02*
Resting SV, ml	90.5† (83-103)	6 (317)	55† (51-59)	6 (161)	104 (104-104)	1 (22)	0.014*
Resting CO, l/min	6.3† (5.6-6.5)	5 (182)	4.0† (3.72-4.65)	4 (100)	6.34 (6.34-6.34)	1 (22)	0.062
Resting SET, ms	330.5 (328-333)	2 (48)	327.5 (305-350)	2 (23)	N/A	-	0.999
Light intensity, %	N/A	-	7.5	1 (12)	100	1 (22)	
Moderate intensity, %	27.2	2 (205)	44.7	3 (72)	N/A	-	
High intensity, %	72.8	9 (550)	47.8	2 (77)	N/A	-	

Values are reported as median (interquartile ranges). BMI indicates body mass index; BSA, body surface area; HR, heart rate; SV, stroke volume; CO, cardiac output; SET, systolic ejection time. * $p<0.05$ overall; † $p<0.05$ for pairwise comparison of dynamic exercise and pharmacological stress (Dunn's test); ‡ $p<0.05$ for pairwise comparison of pharmacological stress and isometric exercise (Dunn's test).

Table S6. Overview of pooled mean changes and 95% CIs of heart rate, stroke volume, cardiac output and systolic ejection time in healthy and patients with aortic stenosis.

		**Heart Rate** [bpm]		**Stroke Volume** [ml]		**Cardiac Output** [l/min]		**Systolic Ejection Time** [ms]	
		Mean change (95% CI)	N	Mean change (95% CI)	N	Mean change (95% CI)	N	Mean change (95% CI)	N
**Healthy**									
**Light**	Dynamic	31.78 (27.82, 35.74)	198	6.59 (2.58, 10.61)	122	2.68 (1.72, 3.64)	152	-20.92 (-45.62, 3.78)	63
	Dobu-tamine	13.71 (7.87, 19.56)	105	5.47 (0.3, 10.63)	75	1.54 (0.69, 2.38)	82	N/A	-
	Isometric	18.44 (10.74, 26.14)	229	-4.17 (-14.37, 6.03)	101	0.82 (-0.26, 1.9)	83	-0.38 (-19.07, 18.32)	96
**Mode-rate**	Dynamic	49.57 (40.03, 59.1)	271	11.64 (5.87, 17.42)	181	4.67 (3.5, 5.84)	240	-51.59 (-100.58, -2.61)	33
	Dobu-tamine	42.83 (36.94, 48.72)	205	6.29 (-2.0, 14.58)	101	4.42 (3.65, 5.19)	133	N/A	-
**High**	Dynamic	89.31 (81.46, 97.17)	787	21.31 (13.42, 29.21)	463	10.45 (8.04, 12.85)	445	-77.83 (-95.88, -59.78)	111
	Dobu-tamine	53.58 (36.53, 70.64)	135	0.98 (-9.32, 11.27)	55	4.98 (2.94, 7.01)	62	-90.0 (-103.71,-76.29)	20
**Aortic stenosis**									
**Light**	Dynamic	N/A	-	N/A	-	N/A	-	N/A	-
	Dobu-tamine	14.0 (9.82, 18.18)	12	8.0 (3.82, 12.18)	12	1.33 (1.11, 1.55)	12	-41 (-52.29, -29.71)	12
	Isometric	5.0 (-1.17, 11.17)	22	-4.0 (-16.43, 8.43)	22	0.21 (-0.64, 1.06)	22	N/A	-
**Mode-rate**	Dynamic	46.45 (42.63, 50.27)	205	N/A	-	N/A	-	N/A	-
	Dobu-tamine	18.66 (2.38, 34.93)	72	13.11 (7.99, 18.23)	72	1.7 (0.74, 2.66)	11	-40 (-45.33, -34.67)	11
**High**	Dynamic	55.32 (47.31, 63.33)	550	-0.96 (-5.27, 3.35)	317	5.3 (3.46, 7.14)	162	-58.94 (-127.52, 9.63)	48
	Dobu-tamine	42.52 (32.77, 52.28)	77	14.06 (-1.62, 29.74)	77	3.92 (2.45, 5.39)	77	N/A	-

N indicates number of stress examinations included into analysis; bpm, beats per minute; ml, milliliter, l/min, liters per minute; ms, milliseconds; N/A, no data available.

Table S7. Multivariate meta-regression analysis for potential variables impacting heterogeneity of HR change.

**HR Change**	**Multivariable Estimate**	**95% CI**	**P value**
Type of Intervention (dynamic, pharmacological or isometric stress)	-5.08	-11.924 to 1.745	0.143
Intensity Level (light, moderate, high)	28.6	23.459 to 33.705	<0.001
Athlete (yes/no)	10.6	-3.265 to 24.467	0.133
Disease Condition (healthy, CoA or AS)	-4.83	-12.611 to 2.952	0.221
Mean Age (years)	-0.297	-0.543 to -0.0503	0.019

Number of observations: 122; Between-study variance: T^2=104.5, Residual variation due to heterogeneity: I^2 residual=20.11%; Proportion of between study variance explained: Adjusted R^2=86.13%; Model Fit for all five covariates: 43.41; p<0.000.

Table S8. Modified version of the Downs and Black checklist.

**Author**	**Publication**	**Reporting**										**External validity**			**Internal validity - bias**							**Internal validity - confounding**						**P.**
		**1**	**2**	**3**	**4**	**5**	**6**	**7**	**8**	**9**	**10**	**11**	**12**	**13**	**14**	**15**	**16**	**17**	**18**	**19**	**20**	**21**	**22**	**23**	**24**	**25**	**26**	**27**
Barber (2016)	Magnetic Resonance-Augmented Cardiopulmonary Exercise Testing: Comprehensively Assessing Exercise Intolerance in Children With Cardiovascular Disease.	1	1	1	1	2	1	1	1		1	0	0	1			1			1	1	0	1			1		0
Roberts (2015)	Real-time aortic pulse wave velocity measurement during exercise stress testing.	1	1	1	1		1	1	0		1	0	0	1			1			1	0	0	0					0
Forouzan (2015)	Non-invasive measurement using cardiovascular magnetic resonance of changes in pulmonary artery stiffness with exercise.	1	1	0	1		1	1	0		0	0	0	1			1			1	1	0	0					0
Claessen (2015)	Pulmonary vascular and right ventricular reserve in patients with normalized resting hemodynamics after pulmonary endarterectomy	1	1	1	1	2	1	1	0		1	0	0	1			1			1	1	1	0			0		0
Barber (2015)	MR augmented cardiopulmonary exercise testing-a novel approach to assessing cardiovascular function.	1	1	1	1		1	1	0		1	0	0	1			1			0	0	1	1					0

Steding-Ehrenborg (2013)	Moderate intensity supine exercise causes decreased cardiac volumes and increased outer volume variations: a cardiovascular magnetic resonance study	1	1	0	1		1	1	0		0	0	0	1			1			1	1	0	0					0
Weber (2011)	High-resolution phase-contrast MRI of aortic and pulmonary blood flow during rest and physical exercise using a MRI compatible bicycle ergometer.	1	1	1	1		1	1	0		1	0	0	1			1			1	0	0	0					1
Punta-wangkoon (2009)	Reduced peripheral arterial blood flow with preserved cardiac output during submaximal bicycle exercise in elderly heart failure	1	1	1	1	2	1	1	1		1	0	0	1			1			1	1	0	0			1		0
Roest (March 2001)	Biventricular response to supine physical exercise in young adults assessed with ultrafast magnetic resonance imaging	1	1	0	1		1	1	0		0	0	0	1			1			1	1	0	0					0
Roest (November 2001)	Prolonged cardiac recovery from exercise in asymptomatic adults late after atrial correction of transposition of the great arteries: evaluation with magnetic resonance flow mapping	1	1	0	1	2	1	1	0		0	0	0	1			1			1	1	0	0			1		0
Niezen (1998)	Measurement of aortic and pulmonary flow with MRI at rest and during physical exercise.	1	1	0	1		1	1	0		0	0	0	1			1			0	0	0	0					0
Stephen-sen (2016)	Changes in blood volume shunting in patients with atrial septal defects: Assessment of heart function with cardio-vascular magnetic resonance during dobutamine stress	1	1	1	1	2	1	1	0		0	0	0	1			1			0	1	0	0			1		0

Pingitore (2013)	Influence of preload and afterload on stroke volume response to low-dose dobutamine stress in patients with non-ischemic heart failure: a cardiac MR study	1	1	1	1	2	1	1	1		1	0	0	1			1			1	1	0	0			0		0
Ahtarovski (2013)	Termination of dobutamine infusion causes transient rebound left heart diastolic dysfunction in healthy elderly women but not in men: a cardiac magnetic resonance study.	1	1	0	1	2	1	1	0		1	0	0	1			1			1	0	0	0			1		0
Ahtarovski (2012)	Left atrial and ventricular function during dobutamine and glycopyrrolate stress in healthy young and elderly as evaluated by cardiac magnetic resonance.	1	1	1	1	2	1	1	0		1	0	0	1			1			1	0	0	0			1		0
Schuster (2011)	Cardiovascular magnetic resonance myocardial feature tracking detects quantitative wall motion during dobutamine stress."	1	1	0	1		1	1	1		0	0	0	1			1			1	0	0	0					0
Manda-paka (2011)	Simultaneous measurement of left and right ventricular volumes and ejection fraction during dobutamine stress cardiovascular magnetic resonance.	1	1	0	1		1	1	0		1	0	0	1			1			1	1	0	0					0
Oosterhof (2005)	Disparity between dobutamine stress and physical exercise magnetic resonance imaging in patients with an intra-atrial correction for transposition of the great arteries.	1	1	1	1	2	1	1	0		1	0	0	1			1			0	0	0	0			0		0

Dodge-Khatami (2002)	Comparable systemic ventricular function in healthy adults and patients with unoperated congenitally corrected transposition using MRI dobutamine stress testing.	1	1	0	1	1	1	1	0		0	0	0	1			1			1	1	0	0			0		0
Pennell (1995)	Assessment of magnetic resonance velocity mapping of global ventricular function during dobutamine infusion in coronary artery disease.	1	1	0	1	1	1	1	0		1	0	0	1			1			1	0	1	0			1		0
van Rugge (1993)	Quantitation of global and regional left ventricular function by cine magnetic resonance imaging during dobutamine stress in normal human subjects.	1	1	1	1		1	1	1		0	0	0	1			1			1	0	0	0					0
Knobels-dorff-Brenken-hoff (2013)	Isometric handgrip exercise during cardiovascular magnetic resonance imaging: set-up and cardiovascular effects.	1	1	1	1		1	1	0		1	0	0	1			1			1	0	0	0					0
D'Alto (2017)	Echocardiographic assessment of right ventricular contractile reserve in healthy subjects.	1	1	1	1		1	1	0		1	0	0	1			1			1	0	0	0					0
D'Andrea (2016)	Right atrial morphology and function in patients with systemic sclerosis compared to healthy controls: a two-dimensional strain study.	1	1	1	1	2	1	1	0		0	0	0	1			1			1	1	1	0			1		0
Wang (2014)	Changes of ventricular and peripheral performance in patients with heart failure and normal ejection fraction: insights from ergometry stress echocardiography.	1	1	1	1	2	1	1	0		1	0	0	1			1			0	0	1	0			1		1

Khouri (2014)	Utility of 3-dimensional echocardiography, global longitudinal strain, and exercise stress echocardiogra-phy to detect cardiac dysfun-ction in breast cancer patients treated with doxorubicin-containing adjuvant therapy.	1	1	1	1	2	1	1	0		1	0	0	1			1			1	0	1	1			1		0
Tan (2013)	Exercise-induced torsional dyssynchrony relates to impaired functional capacity in patients with heart failure and normal ejection fraction	1	1	1	1	2	1	1	0		1	1	0	1			1			0	1	1	0			0		0
Klasnja (2013)	Cardiac power output and its response to exercise in athletes and non-athletes	1	1	0	1	2	1	1	0		0	0	0	1			1			1	0	0	0			1		0
Henein (2013)	Impaired left ventricular systolic function reserve limits cardiac output and exercise capacity in HFpEF patients due to systemic hypertension	1	1	1	1	2	1	1	1		0	1	0	1			1			1	1	0	0			1		0
Lee (2012)	Exercise with a twist: left ventricular twist and recoil in healthy young and middle-aged men, and middle-aged endurance-trained men	1	1	1	1	2	1	1	0		0	0	0	1			1			1	0	0	0			0		0
La Gerche (2012)	Maximal oxygen consumption is best predicted by measures of cardiac size rather than function in healthy adults	1	1	0	1		1	1	0		1	0	0	1			1			1	1	0	0					0
Cheung (2012)	Dynamic dyssynchrony and impaired contractile reserve of the left ventricle in beta-thalassaemia major: an exercise echocardiographic study.	1	1	0	1	2	1	1	0		1	0	0	1			1			1	1	1	0			1		0

Donal (January 2011)	Impact of aortic stenosis on longitudinal myocardial deformation during exercise.	1	1	1	1	2	1	1	1		1	0	0	1			1			1	1	0	1					0
Donal (May 2011)	Comparison of the heart function adaptation in trained and sedentary men after 50 and before 35 years of age	1	1	1	1	2	1	1	0		0	0	0	1			1			1	1	0	0			1		0
Bombardi-ni (2011)	Abnormal shortened diastolic time length at increasing heart rates in patients with abnormal exercise-induced increase in pulmonary artery pressure.	1	1	1	1	2	1	1	1		0	0	0	1			1			1	1	1	0			0		0
Dini (2010)	Peak power output to left ventricular mass: an index to predict ventricular pumping performance and morbidity in advanced heart failure	1	1	1	1	2	1	1	1		1	0	0	1			1			1	0	1	0			0		0
Argiento (2010)	Exercise stress echocardiography for the study of the pulmonary circulation	1	1	1	1		1	1	0		0	0	0	1			1			1	0	0	0					0
Schuster (2009)	Cardiac function during exercise in obese prepubertal boys: effect of degree of obesity	1	1	1	1	2	1	1	0		0	0	0	1			1			1	1	1	0			1		0
Goodman (2009)	Left ventricular contractile function is preserved during prolonged exercise in middle-aged men	1	1	1	1		1	1	0		0	0	0	1			1			1	0	1	0					0
Cotrim (2008)	Do healthy individuals develop intraventricular gradients during exertion?	1	1	1	1		1	1	1		0	0	0	1			1			1	1	0	0					0
Bombardi-ni (2008)	Diastolic time - frequency relation in the stress echo lab: filling timing and flow at different heart rates.	1	1	1	1		1	1	1		0	0	0	1			1			1	1	0	0					0

De Souza (2007)	A stress echocardiography study of cardiac function during progressive exercise in pediatric oncology patients treated with anthracyclines.	1	1	1	1	2	1	1			0	0	0	1			1			0	0	1	1			0		0
Rowland (2005)	Effect of pectus excavatum deformity on cardiorespiratory fitness in adolescent boys.	1	1	0	1	2	1	1	0		0	0	0	1			1			1	1	0	0			1		0
Sagiv (2000)	Left ventricular contractility and function at peak aerobic and anaerobic exercises.	1	1	1	1		1	1	1		0	0	0	1			1			1	1	1	0					0
Auerbach (1999)	Attenuated responses of Doppler-derived hemodynamic parameters during supine bicycle exercise in heart transplant recipients.	1	1	0	1	2	1	1	0		1	0	0	1			1			1	0	0	0			0		0
Fisman (1990)	Altered left ventricular volume and ejection fraction responses to supine dynamic exercise in athletes.	1	1	1	1	2	1	1	1		1	0	0	1			1			1	1	1	0			1		0
Ginzton (1989)	Effect of long-term high intensity aerobic training on left ventricular volume during maximal upright exercise.	1	1	1	1	1	1	1	0		0	0	0	1			1			1	1	1	0			0		0
Thompson (1987)	Comparison of ventricular volumes in normal and post-myocardial infarction subjects.	1	1	1	1	1	1	1	1		0	0	0	1			1			0	1	0	0			1		0
Mehdirad (1987)	Evaluation of left ventricular function during upright exercise: correlation of exercise Doppler with postexercise two-dimensional echocardiographic results.	1	1	1	1	1	1	1	1		0	0	0	1			1			1	1	0	0			0		0
Gardin (1986)	Studies of Doppler aortic flow velocity during supine bicycle exercise.	1	1	1	1		1	1	1		0	0	0	1			1			1	0	0	0					0

Bryg (1986)	Effect of coronary artery disease on Doppler-derived parameters of aortic flow during upright exercise	1	1	0	1	1	1	1	1		0	0	0	1			1			1	1	0	0			0		0
Lau (2014)	Dobutamine stress echocardiography for the assessment of pressure-flow relationships of the pulmonary circulation	1	1	1	1	2	1	1	0		1	0	0	1			1			1	1	0	0			1		0
Maras (2013)	Patterns of cardiac dysfunction coinciding with exertional breathlessness in hypertrophic cardiomyopathy	1	1	1	1	1	1	1	1		0	0	0	1			1			1	0	0	0			0		0
Brili (2007)	Dobutamine stress echocardiography for the evaluation of cardiac reserve late after Fontan operation	1	1	0	1	1	1	1	1		1	0	0	1			1			1	0	1	0			1		0
Arshad (2004)	Systole-diastole mismatch in hypertrophic cardiomyopathy is caused by stress induced left ventricular outflow tract obstruction.	1	1	0	1	2	1	1	1		0	0	0	1			1			1	0	0	0			0		0
Cnota (2003)	Cardiovascular physiology during supine cycle ergometry and dobutamine stress.	1	1	1	1		1	1	1		1	0	0	1			1			0	0	0	0					0
Duncan (2001)	Long axis electromechanics during dobutamine stress in patients with coronary artery disease and left ventricular dysfunction	1	1	1	1	2	1	1	1		0	0	0	1			1			1	1	0	0			0		0
Marmor (1996)	Evaluation of contractile reserve by dobutamine echocardiography: noninvasive estimation of the severity of heart failure.	1	1	1	1	0	1	1	1		1	1	0	1			1			0	1	1	0			0		0

Blom-strand (1995)	Cardiovascular effects of dobutamine stress testing in healthy women.	1	1	1	1		1	1	1		0	1	0	1			1			1	0	0	0					0
Weiner (2012)	The impact of isometric handgrip testing on left ventricular twist mechanics.	1	1	1	1		1	1	1		1	0	0	1			1			0	0	1	0					0
Bozkurt (2006)	Echocardiographic findings in patients with Behcet's disease.	1	1	1	1	2	1	1	0		1	0	0	1			1			1	1	1	0			1		0
Lev (1998)	Exercise-induced aortic flow parameters in early postmenopausal women and middle-aged men.	1	1	1	1	1	1	1	0		0	1	0	1			1			1	0	1	0			0		0
Fisman (1991)	Pronounced reduction of aortic flow velocity and acceleration during heavy isometric exercise in coronary artery disease.	1	1	1	1	1	1	1	1		0	0	0	1			1			1	1	1	0			1		1
Bamrah (1991)	Static versus dynamic exercise: effects on Doppler echocardiographic indices of left ventricular performance.	1	1	1	1	0	1	1	1		0	0	0	1			1			1	0	0	0			0		0
Krzemin-ski (1989)	Effect of endurance training on cardiovascular response to static exercise performed with untrained muscles.	1	1	0	1		1	1	0		1	0	0	1			1			1	0	0	0					0

**Aortic Stenosis**																												
Pérez Del Villar (2017)	The Functional Significance of Paradoxical Low-Gradient Aortic Valve Stenosis: Hemo-dynamic Findings During Cardiopulmonary Exercise Testing.	1	1	1	1		1	1	0		0	0	0	1			1			1	0	0	0					0
Lancellotti (2012)	Determinants and prognostic significance of exercise pulmo-nary hypertension in asymp-tomatic severe aortic stenosis.	1	1	1	1	2	1	1	1	1	1	0	0	1			1			1	0	1	0			1		0
Marechaux (2010)	Usefulness of exercise-stress echocardiography for risk stratification of true asymp-tomatic patients with aortic valve stenosis.	1	1	1	1		1	1	1	1	1	0	0	1			1			1	0	1	0					0
Lancellotti (2008)	Determinants of an abnormal response to exercise in patients with asymptomatic valvular aortic stenosis.	1	1	1	1		1	1	1		1	0	0	1			1			1	1	0	0					0
Legget (1996)	Gender differences in left ventricular function at rest and with exercise in asymptomatic aortic stenosis.	1	1	1	1	2	1	1	0		1	0	0	1			1			0	1	0	1			1		0
Burwash (1994)	Flow dependence of measures of aortic stenosis severity during exercise.	1	1	1	1		1	1	0		1	0	0	1			1			1	0	0	0					0
Otto (1992)	Physiologic changes with maximal exercise in asymp-tomatic valvular aortic stenosis assessed by Doppler echocardiography.	1	1	1	1		1	1	1		1	0	0	1			1			0	1	0	0					0
Mahfouz (2015)	Left ventricular restrictive filling pattern and the presence of contractile reserve in patients with low-flow/low-gradient severe aortic stenosis.	1	1	1	1	2	1	1	0		1	0	0	1			1			1	0	0	0			1		0

Bartko (2013)	Two-dimensional strain for the assessment of left ventricular function in low flow-low gradient aortic stenosis, relationship to hemodynamics, and outcome: a substudy of the multicenter TOPAS study.	1	1	1	1		1	1	0	1	1	0	0	1			1			1	0	0	0					0
Takeda (1999)	The relation between transaortic pressure difference and flow during dobutamine stress echocardiography in patients with aortic stenosis.	1	1	1	1		1	1	1		1	0	0	1			1			1	0	0	1					0
Lin (1998)	Dobutamine stress Doppler hemodynamics in patients with aortic stenosis	1	1	1	1		1	1	1	1	1	1	0	1			1			1	1	1	1					0
Tardif (1997)	Simultaneous determination of aortic valve area by the Gorlin formula and by transesophageal echocardiography under different transvalvular flow conditions. Evidence that anatomic aortic valve area does not change with variations in flow in aortic stenosis.	1	1	1	1		1	1	1		0	0	0	1			1			1	0	0	0					0
Casale (1992)	Effects of dobutamine on Gorlin and continuity equation valve areas and valve resistance in valvular aortic stenosis	1	1	1	1		1	1	1		0	0	0	1			1			1	0	0	0					0
Little (2007)	Impact of blood pressure on the Doppler echocardiographic assessment of severity of aortic stenosis.	1	1	1	1		1	1	1		0	0	0	1			1			1	0	1	0					0

**Aortic Coarctation**																												
Pedersen (2010)	Blood flow measured by magnetic resonance imaging at rest and exercise after surgical bypass of aortic arch obstruction.	1	1	1	1	1	1	1	0		0	0	0	1			1			1	1	0	0			1		0
Kimball (1986)	Persistent ventricular adaptations in postoperative coarctation of the aorta.	1	1	1	1	1	1	1	0		0	0	0	1			1			1	0	1	0			1		0
Carpenter (1985)	Left ventricular hyperkinesia at rest and during exercise in normotensive patients 2 to 27 years after coarctation repair.	1	1	1	1	2	1	1	0		0	1	0	1			1			1	0	0	0			0		0
Weber (1993)	Discrepancies in aortic growth explain aortic arch gradients during exercise.	1	1	1	1		1	1	1		0	0	0	1			1			1	0	0	0					0

Reporting: 1. Hypothesis/aims/objectives clearly stated **2**. Main outcome measures clearly described **3**. Characteristics of patients/subjects clearly described **4**. Interventions of interest clearly described **5**. Distribution of principal confounders in each group clearly described **6**. Main findings clearly described **7**. Estimates of random variability in the data provided **8**. Important adverse events reported **9**. Characteristics of patients lost to follow-up described **10**. Actual probability values reported

External validity: 11. Participants approached representative of entire population **12**. Participants recruited representative of entire population **13**. Staff, places and facilities representative of majority of population

Internal validity-bias: 14. Blinding of study subjects **15**. Blinding of assessors **16**. Data based on data-dredging clearly stated **17**. Adjustment of different length of follow-up or duration between case and control **18**. Appropriate statistical tests used **19**. Compliance to intervention reliable **20**. Main outcome measure reliable and valid

Internal validity-confounding: 21. Intervention groups or case-controls recruited from same population **22**. Intervention groups or case-controls recruited at the same time **23**. Study subjects randomized to the interventions **24**. Was concealed randomization to allocation undertaken **25**. Adequate adjustment made in the analysis of confounders **26**. Patient losses accounted for

Power (P.): 27. Sufficiently powered cohort size

Supplementary Figures

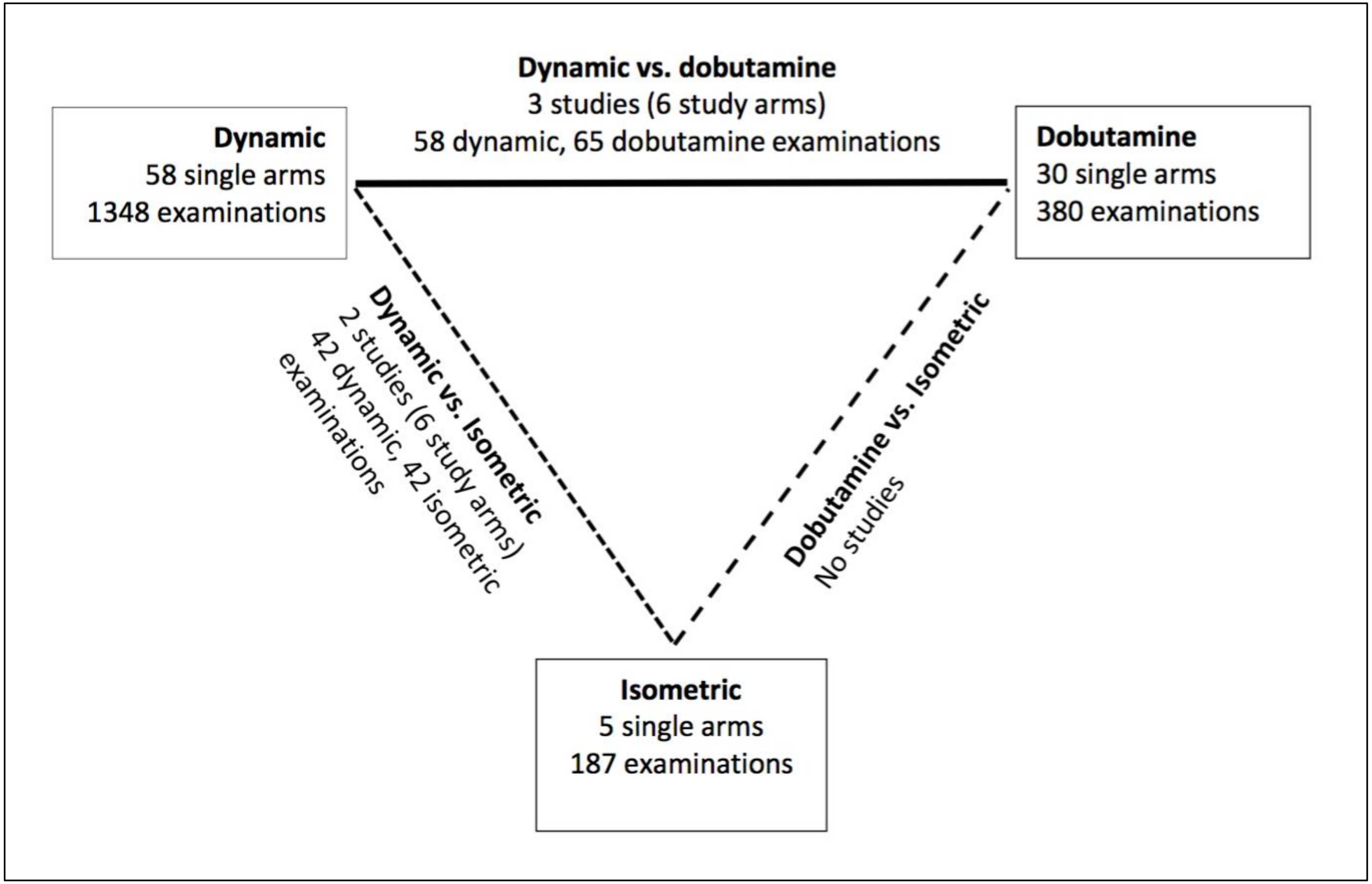

Figure S1. Network of evidence for findings in healthy subjects (adapted from Salcher et al., Circ Cardiovasc Interv. 2016; 9). Different types of stress testing (dynamic, isometric, pharmacological stress) are shown at the edges. Lines connecting the edges represent the limited number of studies directly comparing the different stress types to each other.

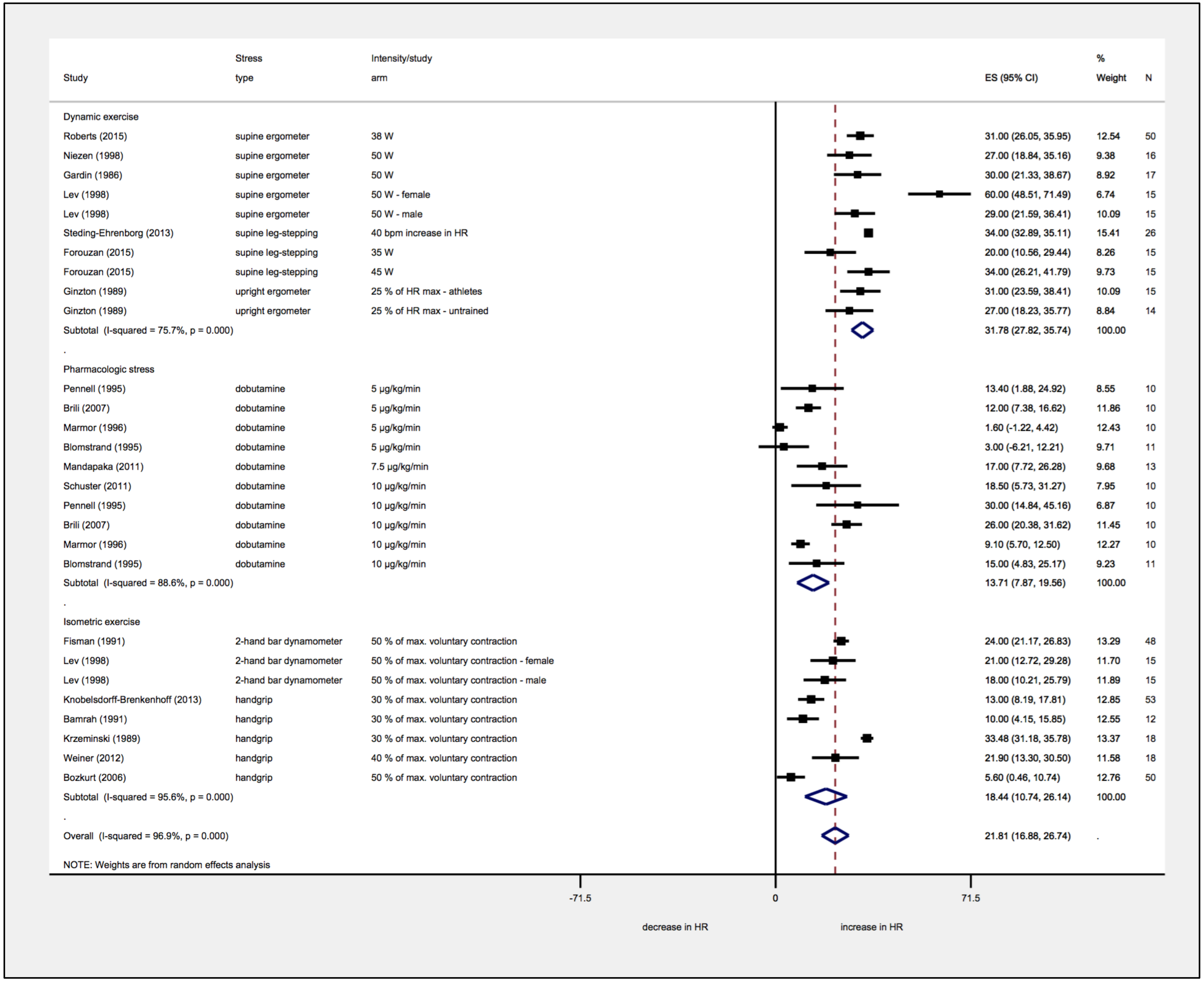

Figure S2. HR Changes [bpm] in healthy subjects for light intensity stress.

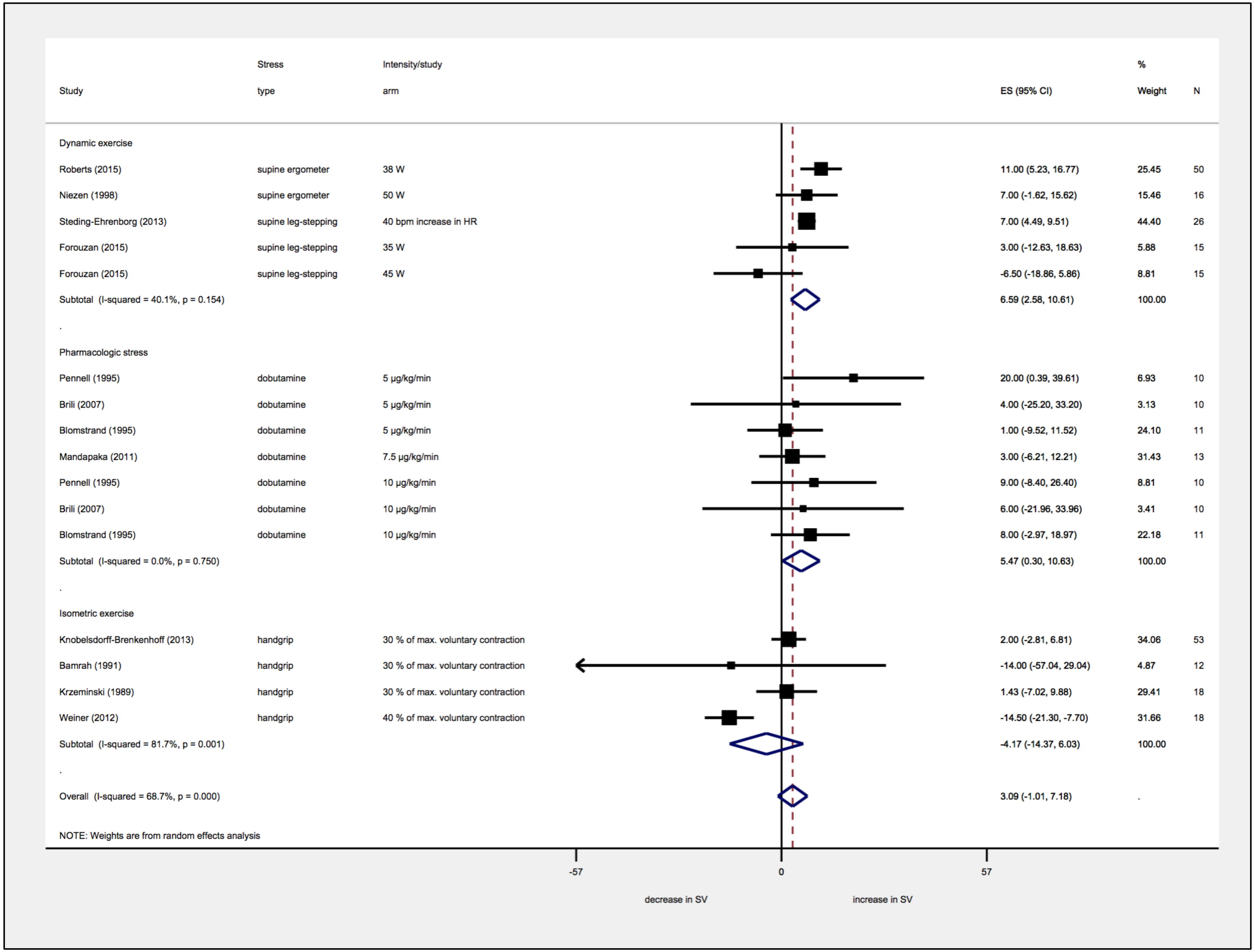

Figure S3. SV Changes [ml] in healthy subjects for light intensity stress.

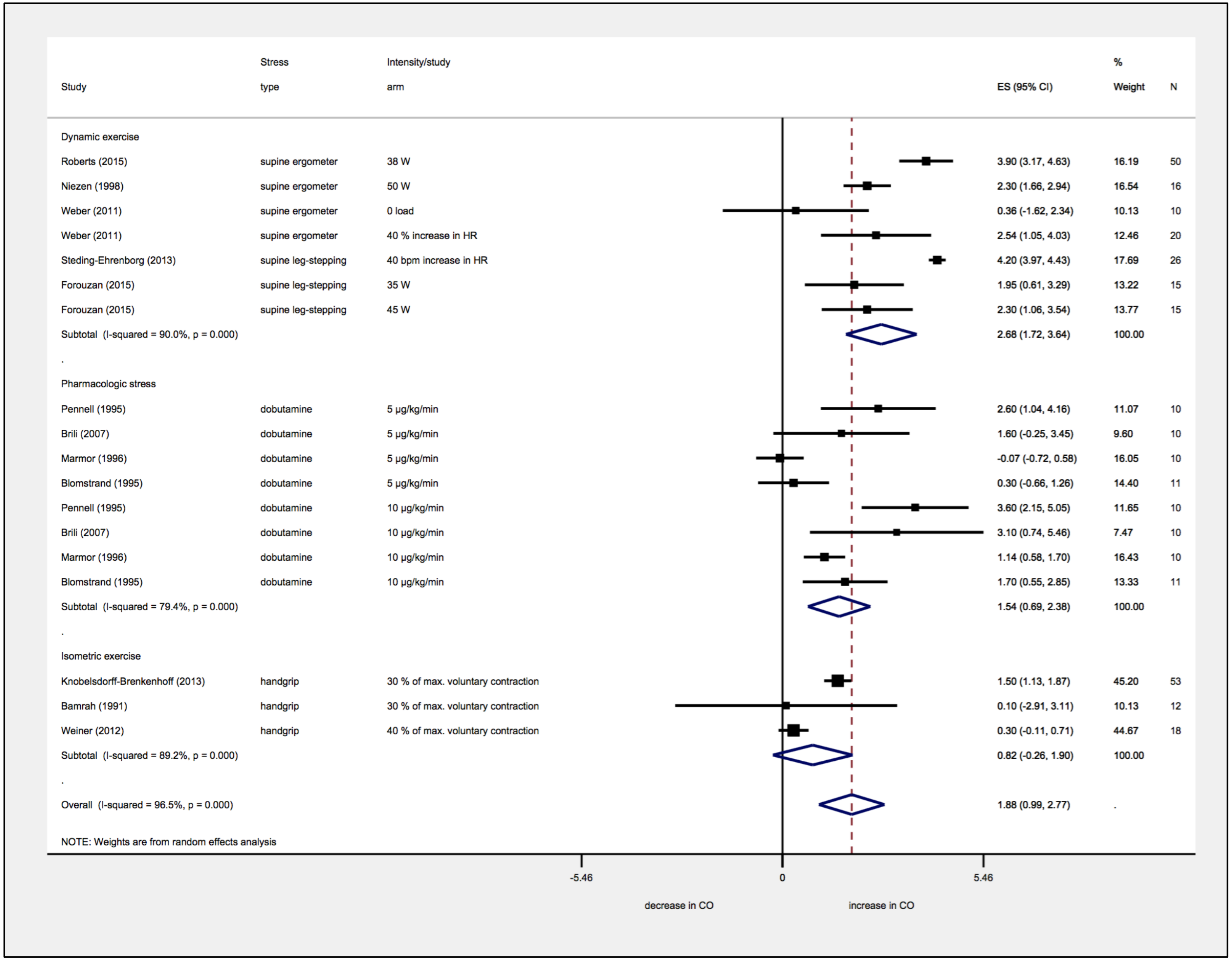

Figure S4. CO Changes [l/min] in healthy subjects for light intensity stress.

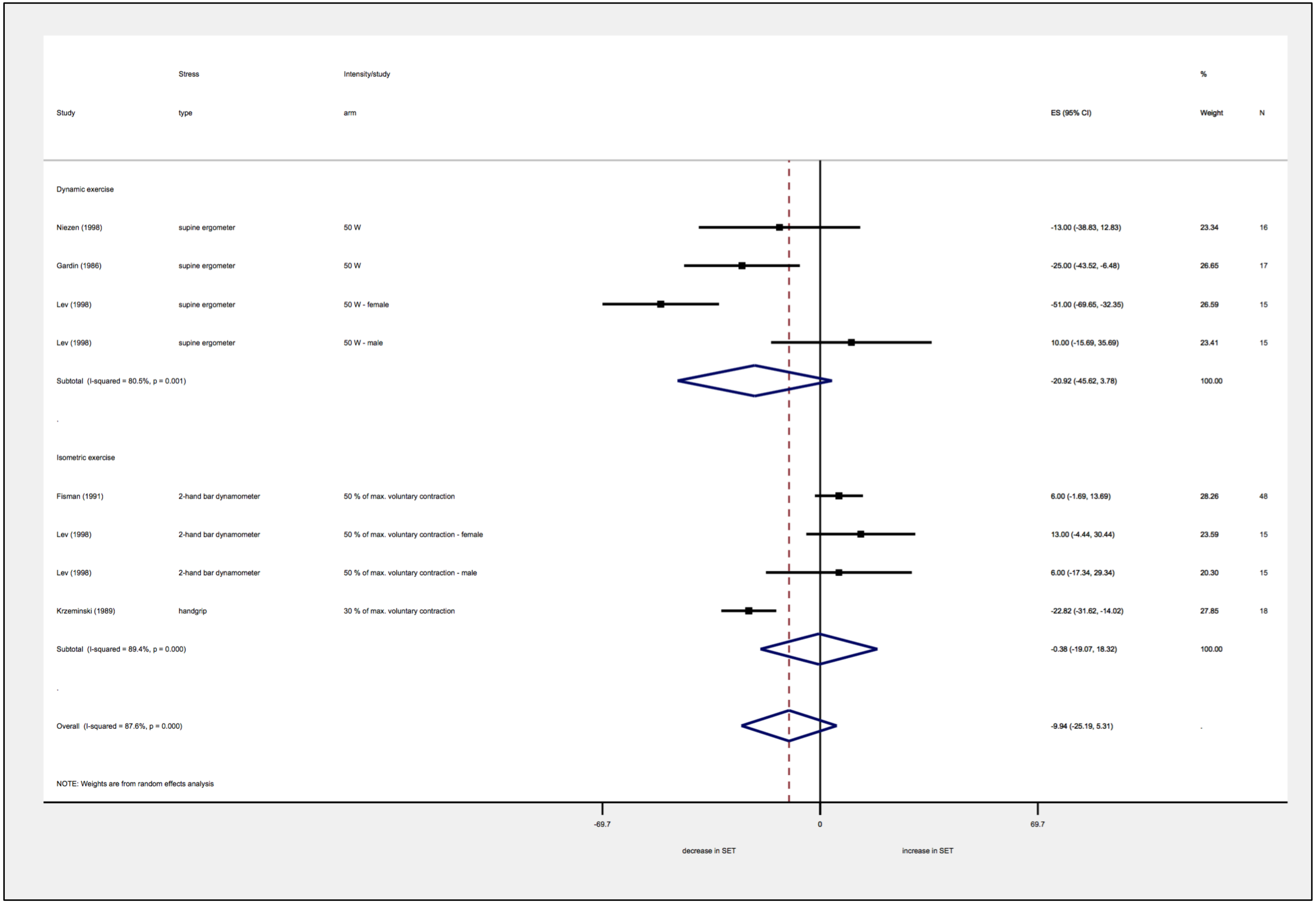

Figure S5. SET Changes [ms] in healthy subjects for light intensity stress.

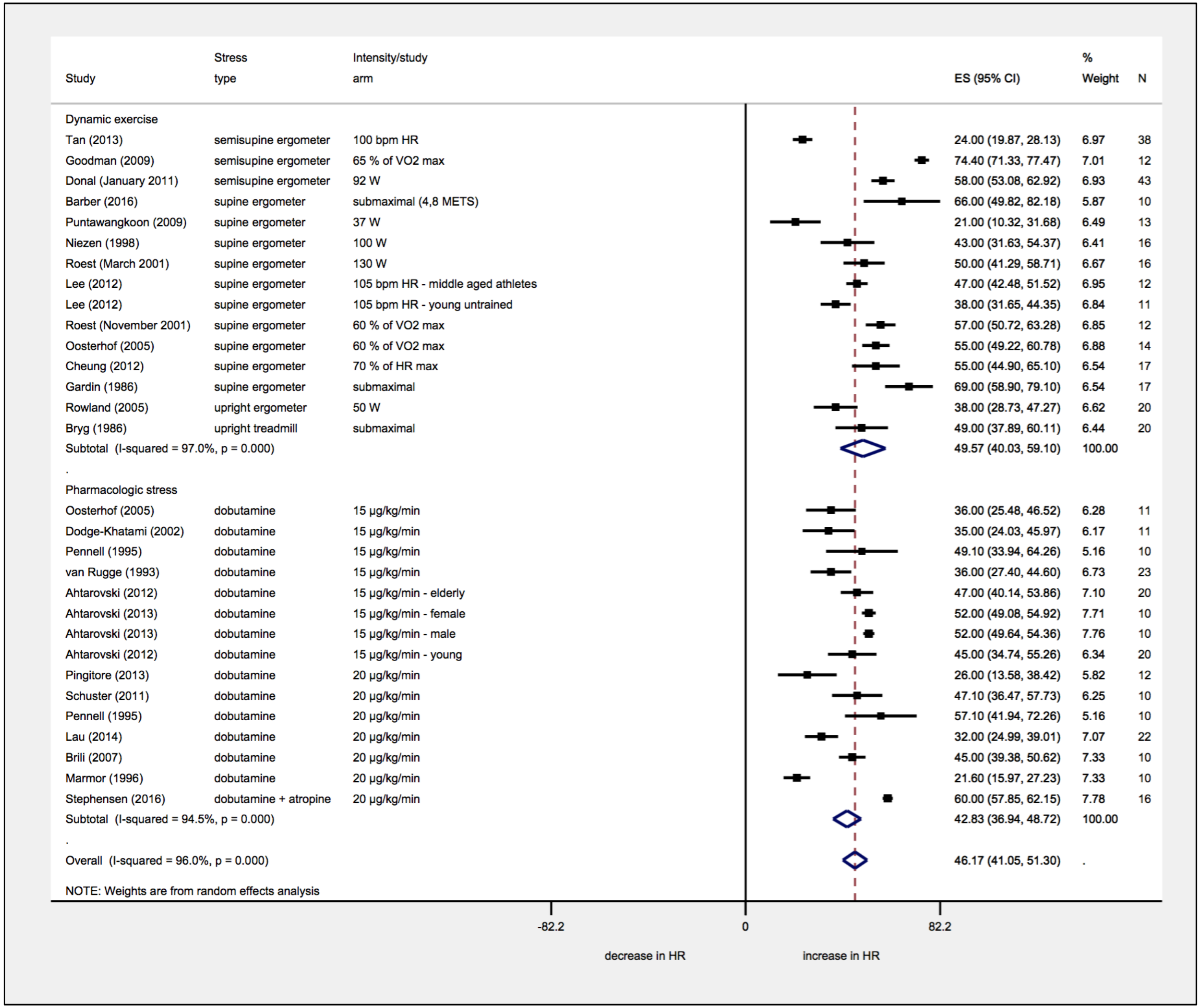

Figure S6. HR Changes [bpm] in healthy subjects for moderate intensity stress.

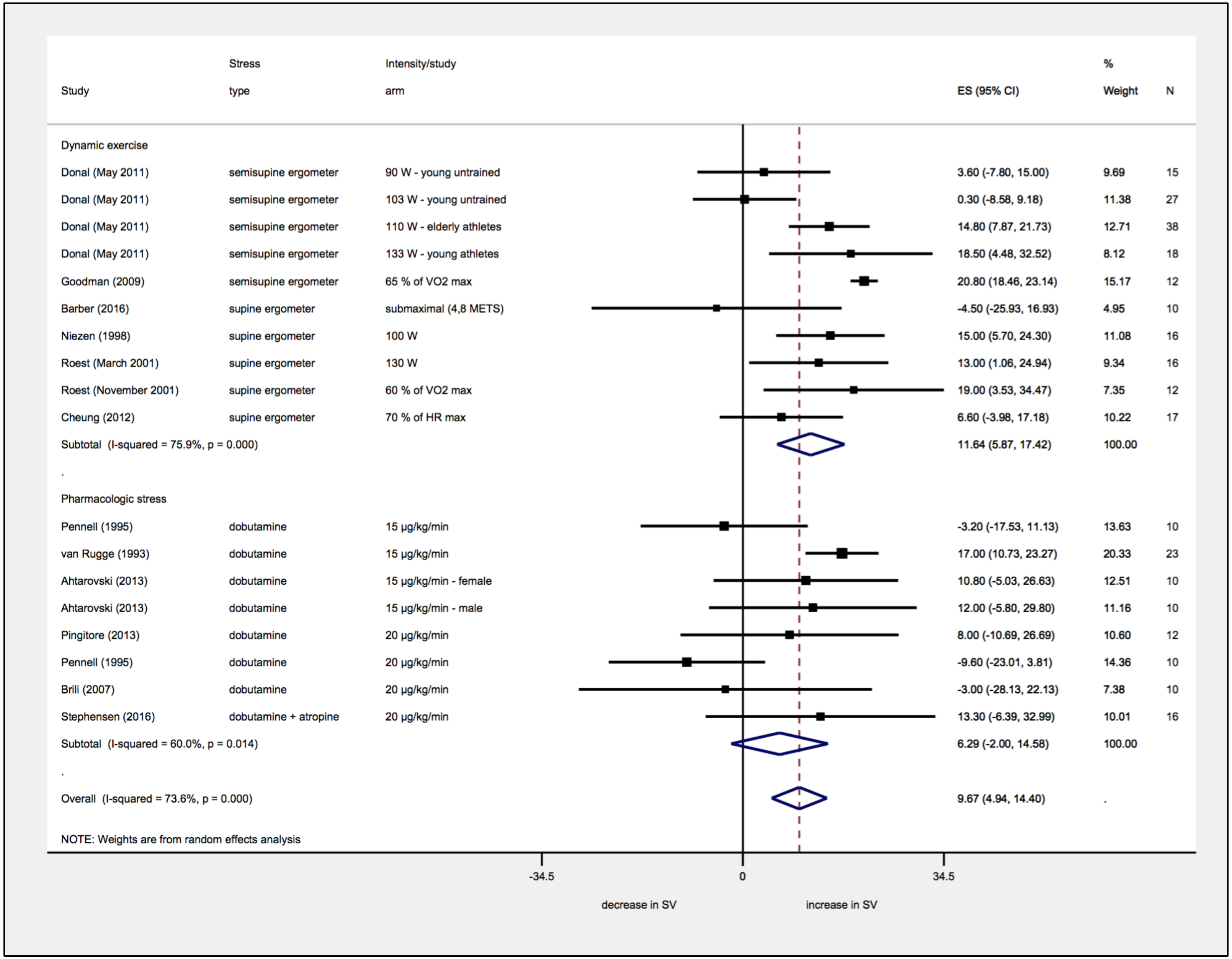

Figure S7. SV Changes [ml] in healthy subjects for moderate intensity stress.

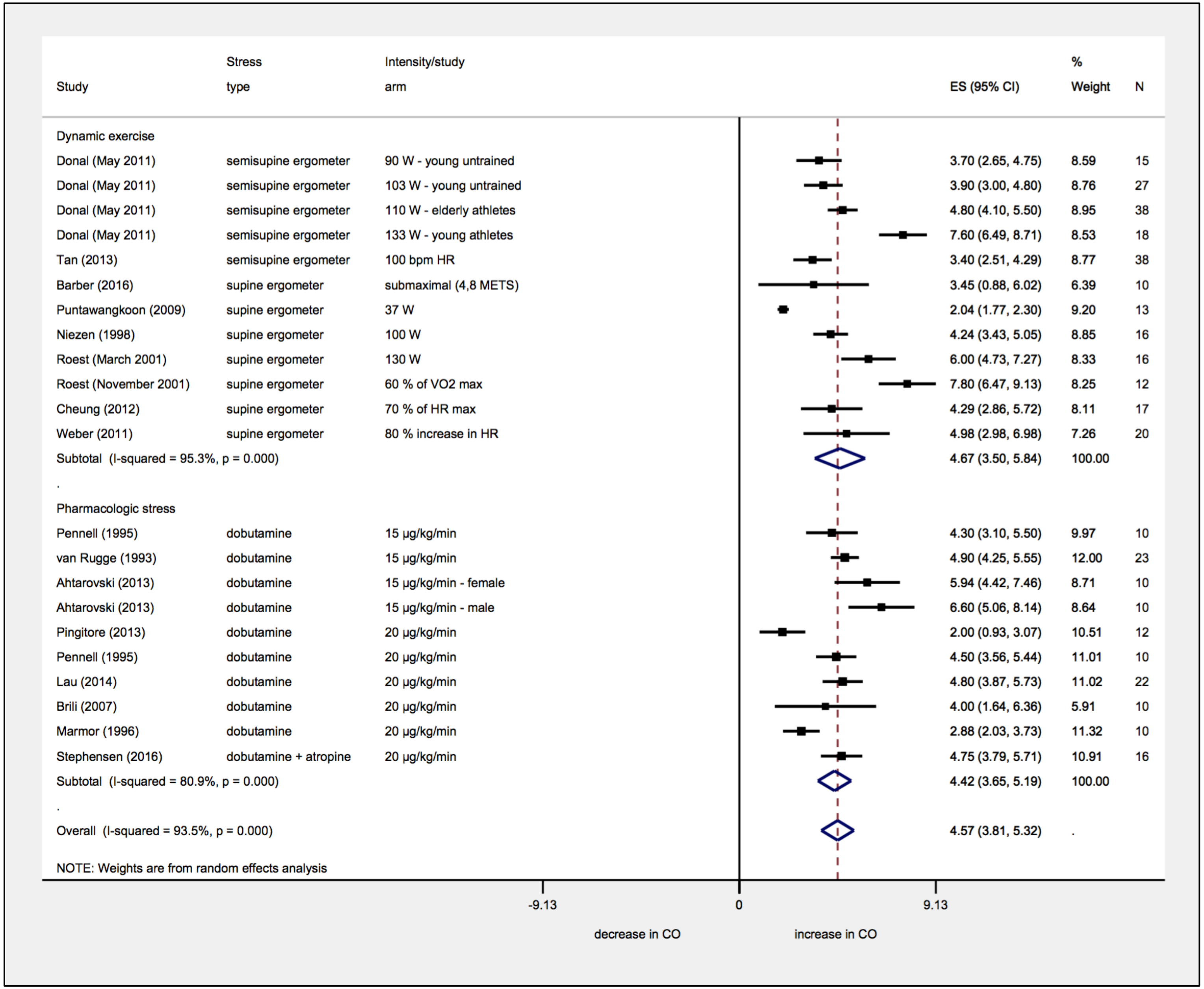

Figure S8. CO Changes [l/min] in healthy subjects for moderate intensity stress.

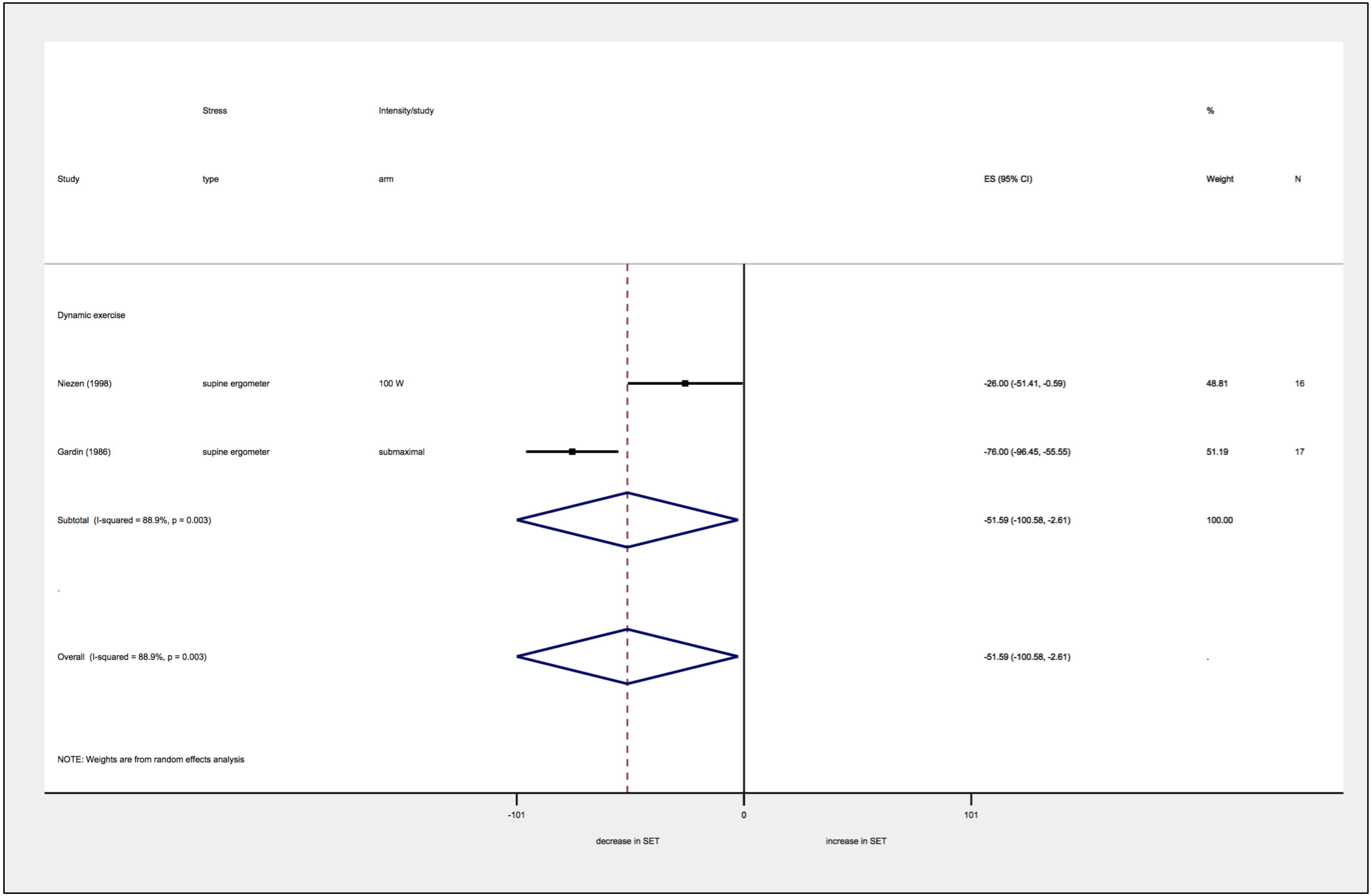

Figure S9. SET Changes [ms] in healthy subjects for moderate intensity stress.

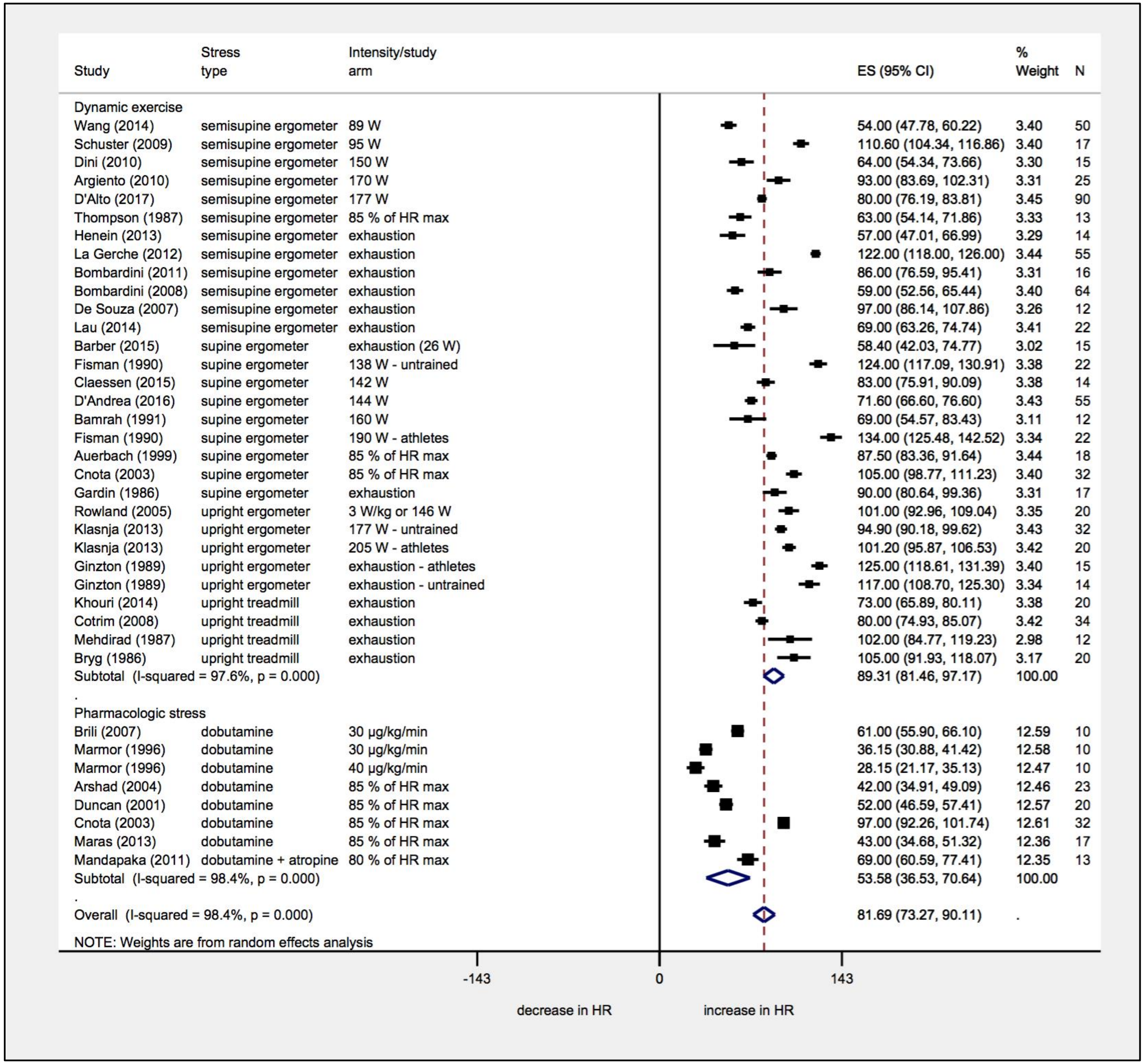

Figure S10. HR Changes [bpm] in healthy subjects for high intensity stress.

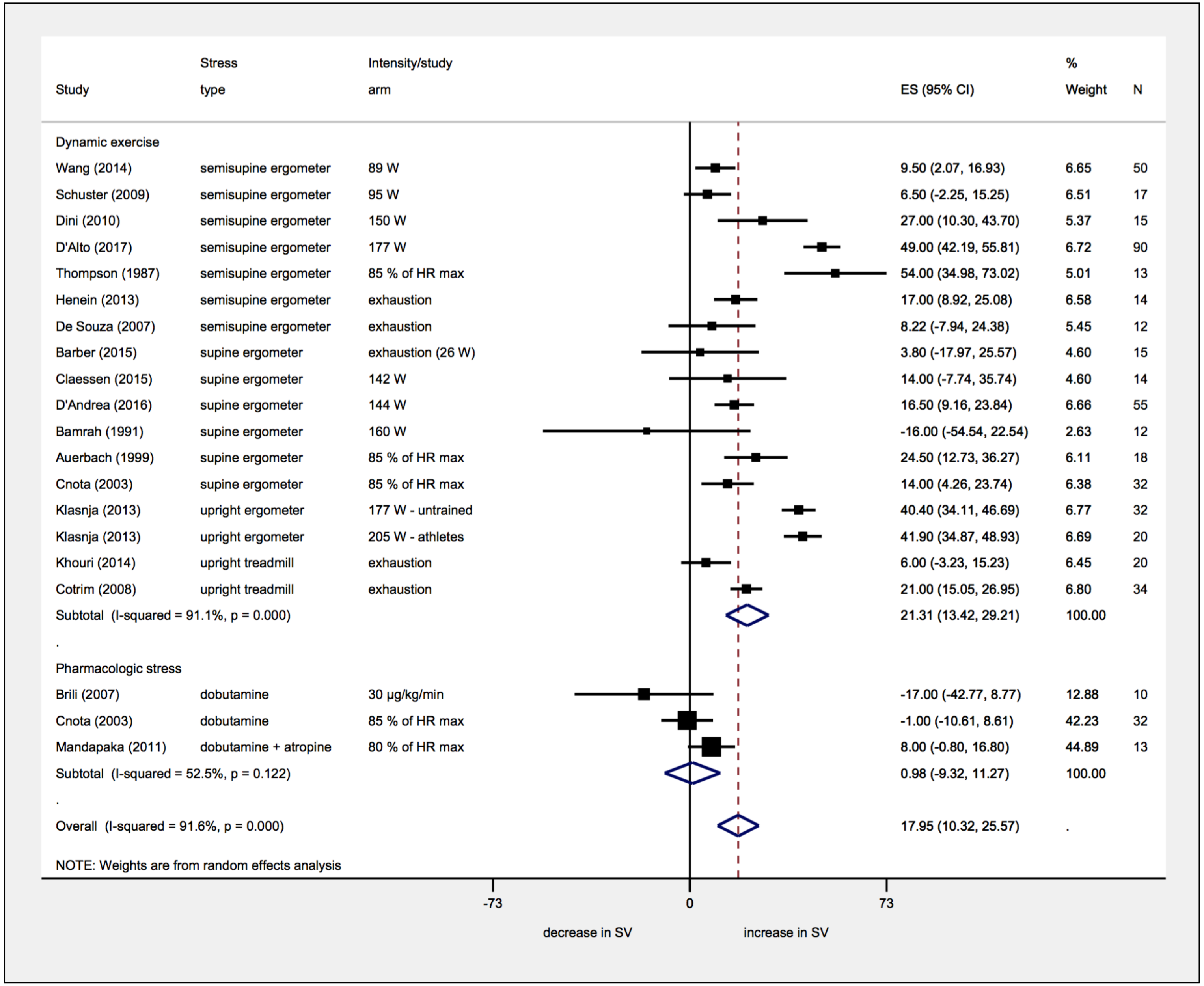

Figure S11. SV Changes [ml] in healthy subjects for high intensity stress.

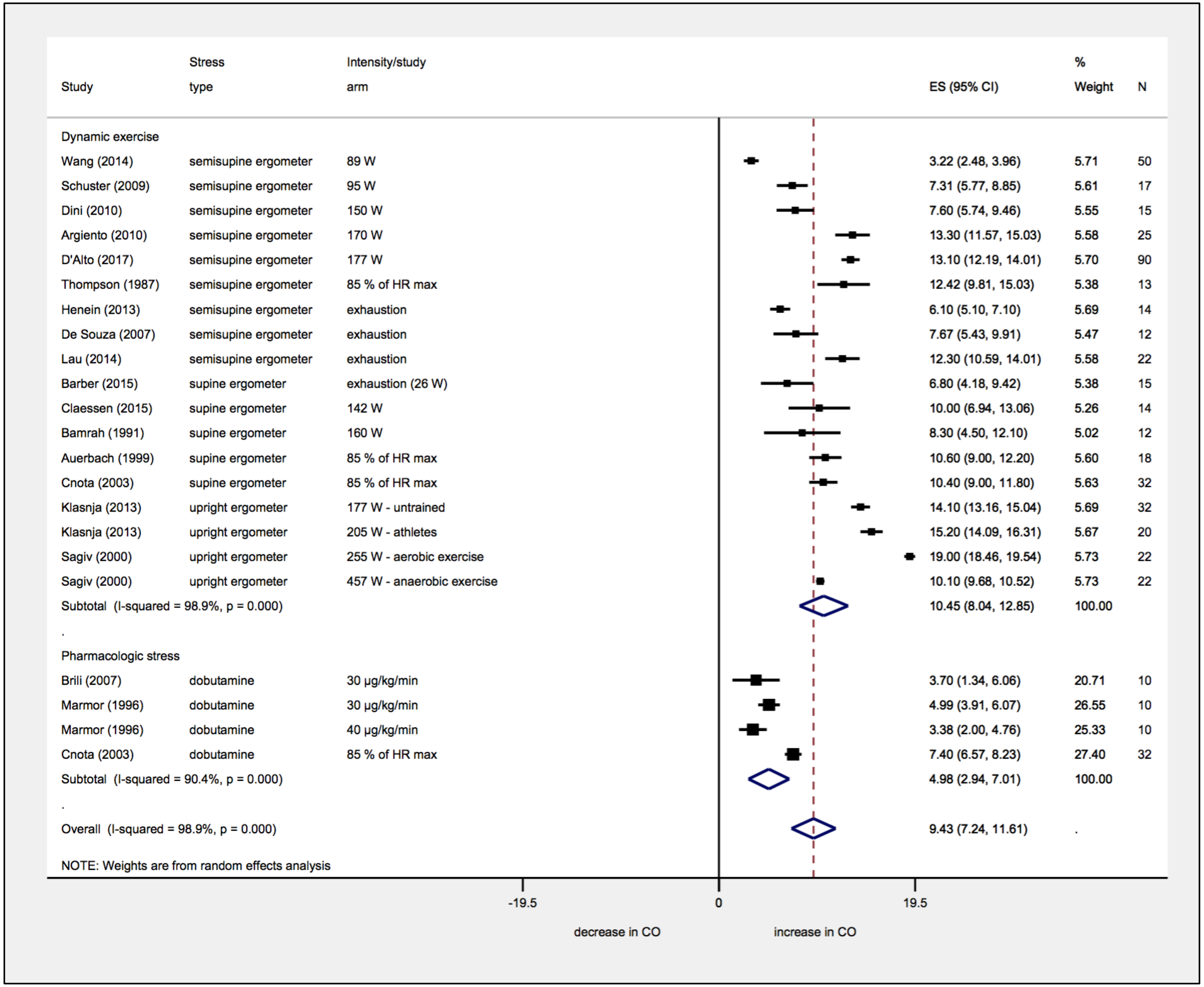

Figure S12. CO Changes [l/min] in healthy subjects for high intensity stress.

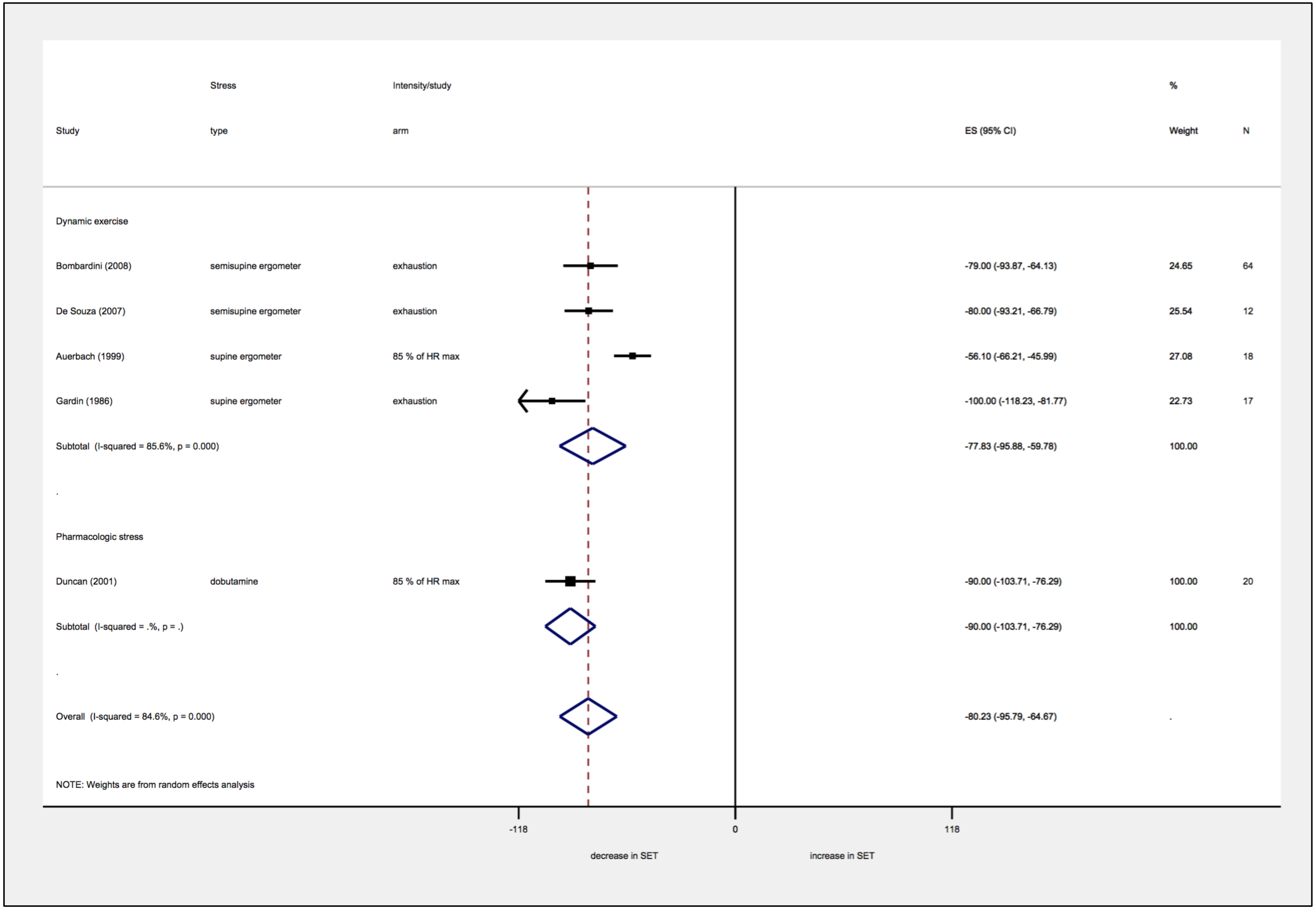

Figure S13. SET Changes [ms] in healthy subjects for high intensity stress.

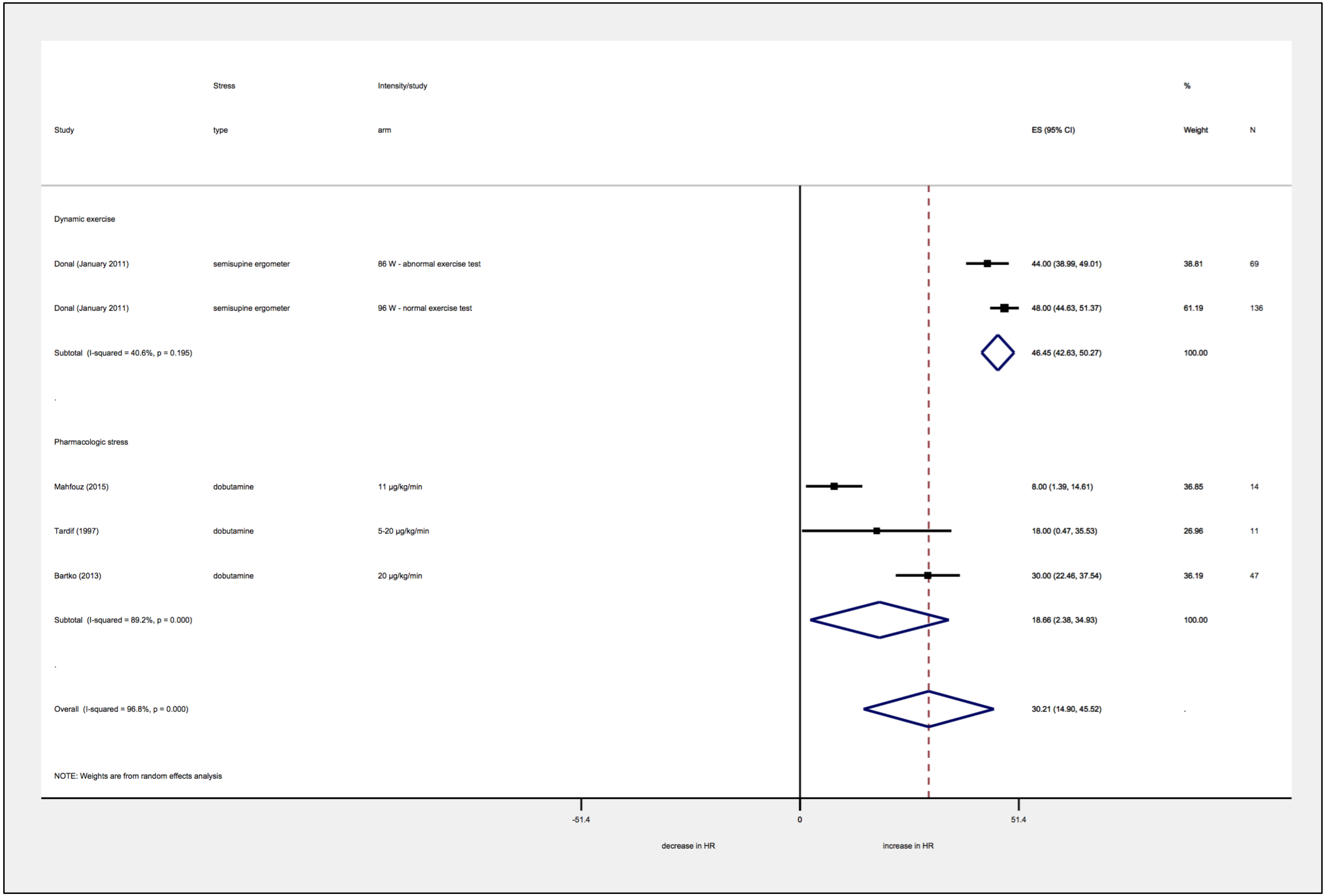

Figure S14. HR Changes [bpm] in patients with aortic stenosis for moderate intensity stress.

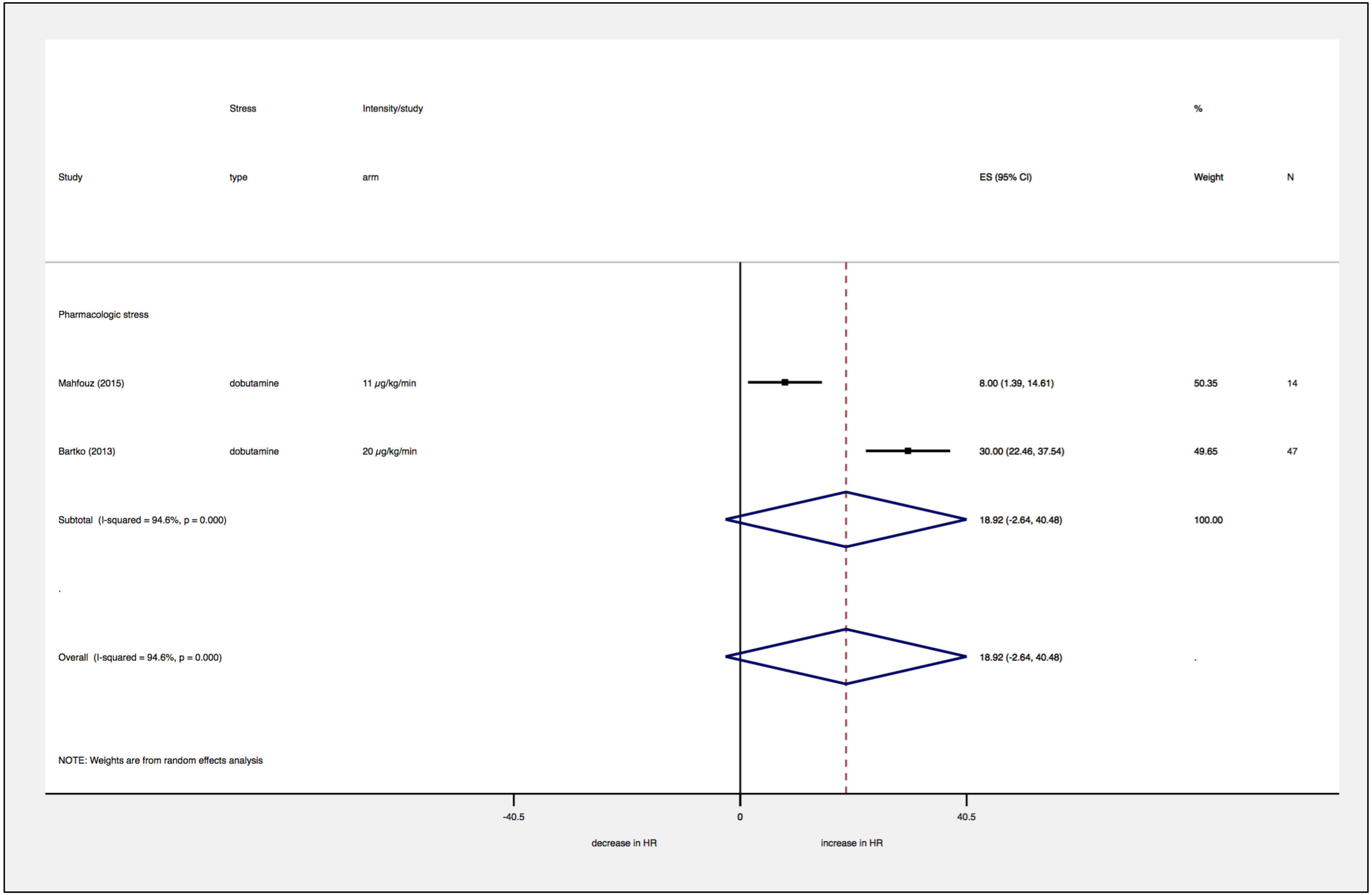

Figure S14 (a). HR Changes [bpm] in patients with low-flow, low-gradient aortic stenosis for moderate intensity stress.

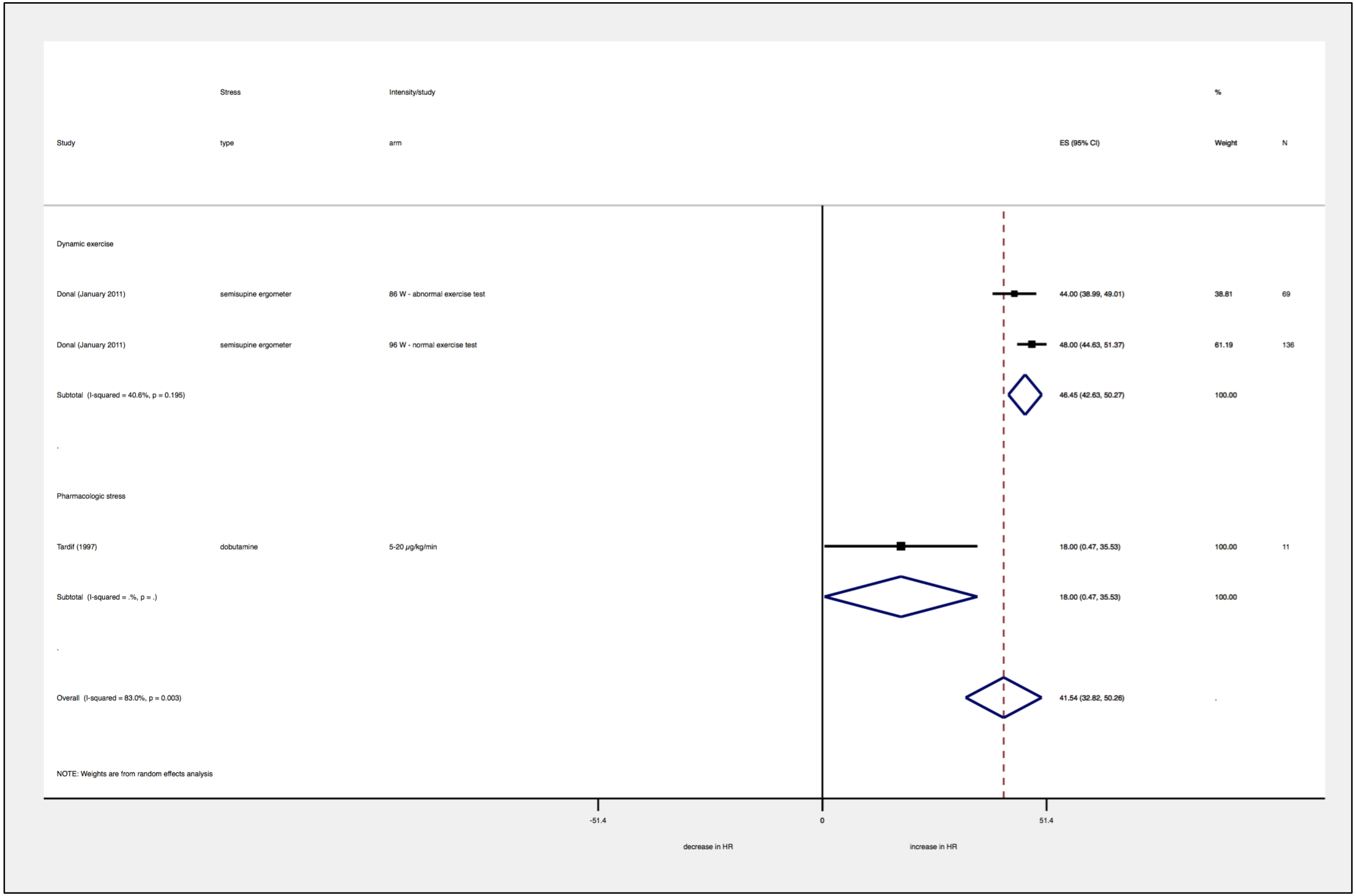

Figure S14 (b). HR Changes [bpm] in patients with aortic stenosis after excluding low-flow, low-gradient patients for moderate intensity stress.

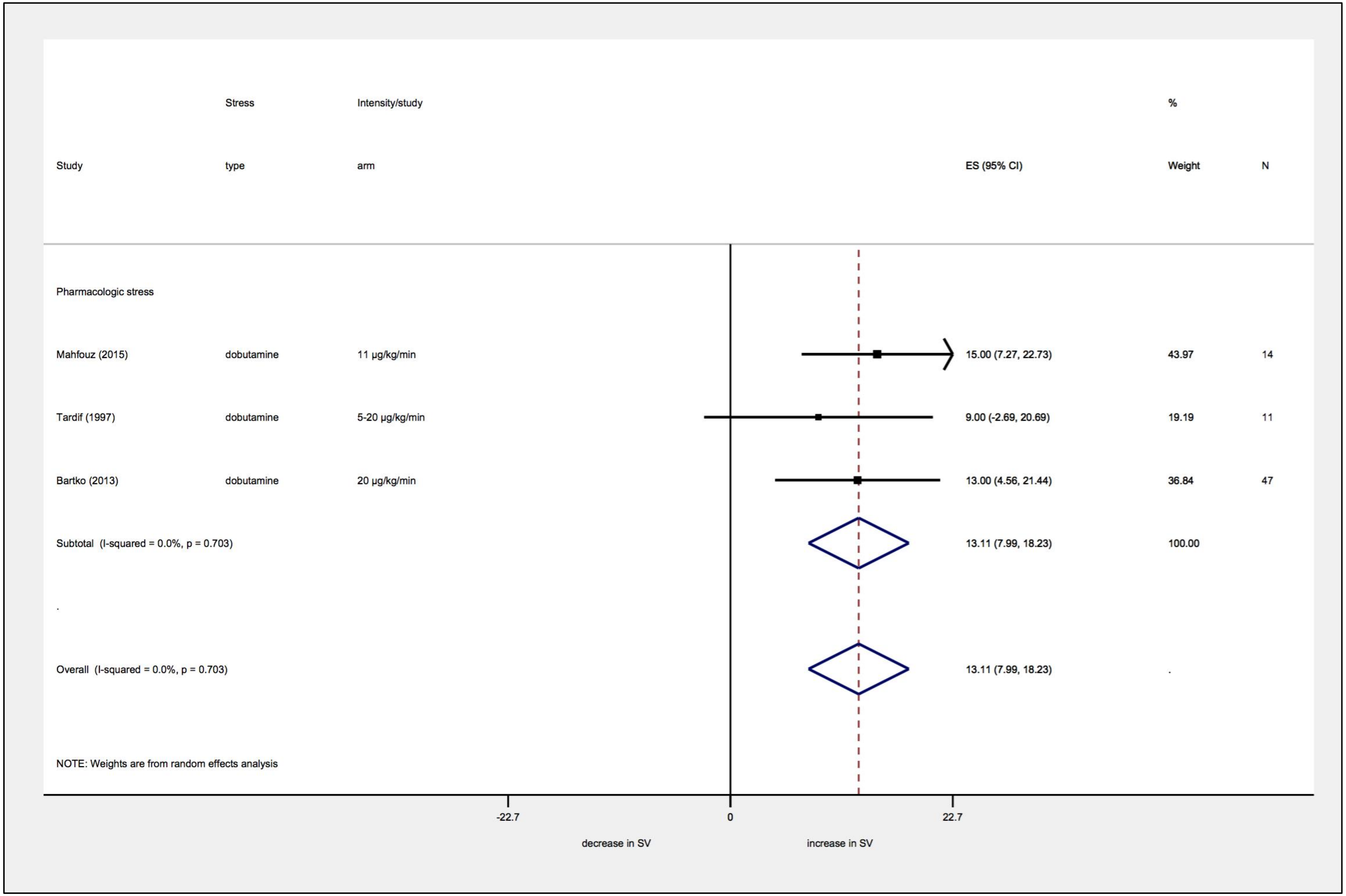

Figure S15. SV Changes [ml] in patients with aortic stenosis for moderate intensity stress.

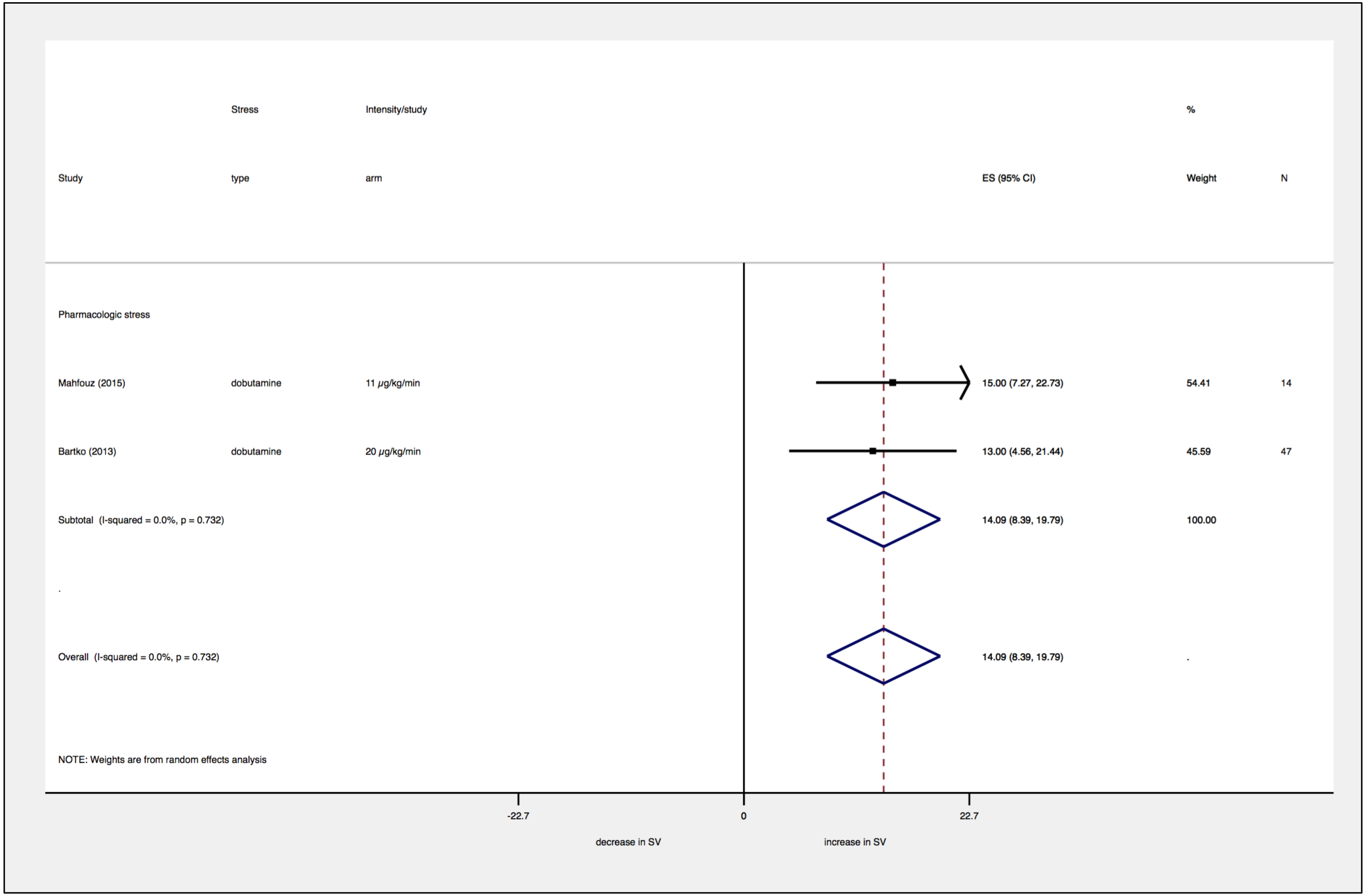

Figure S15 (a). SV Changes [ml] in patients with low-flow, low-gradient aortic stenosis for moderate intensity stress.

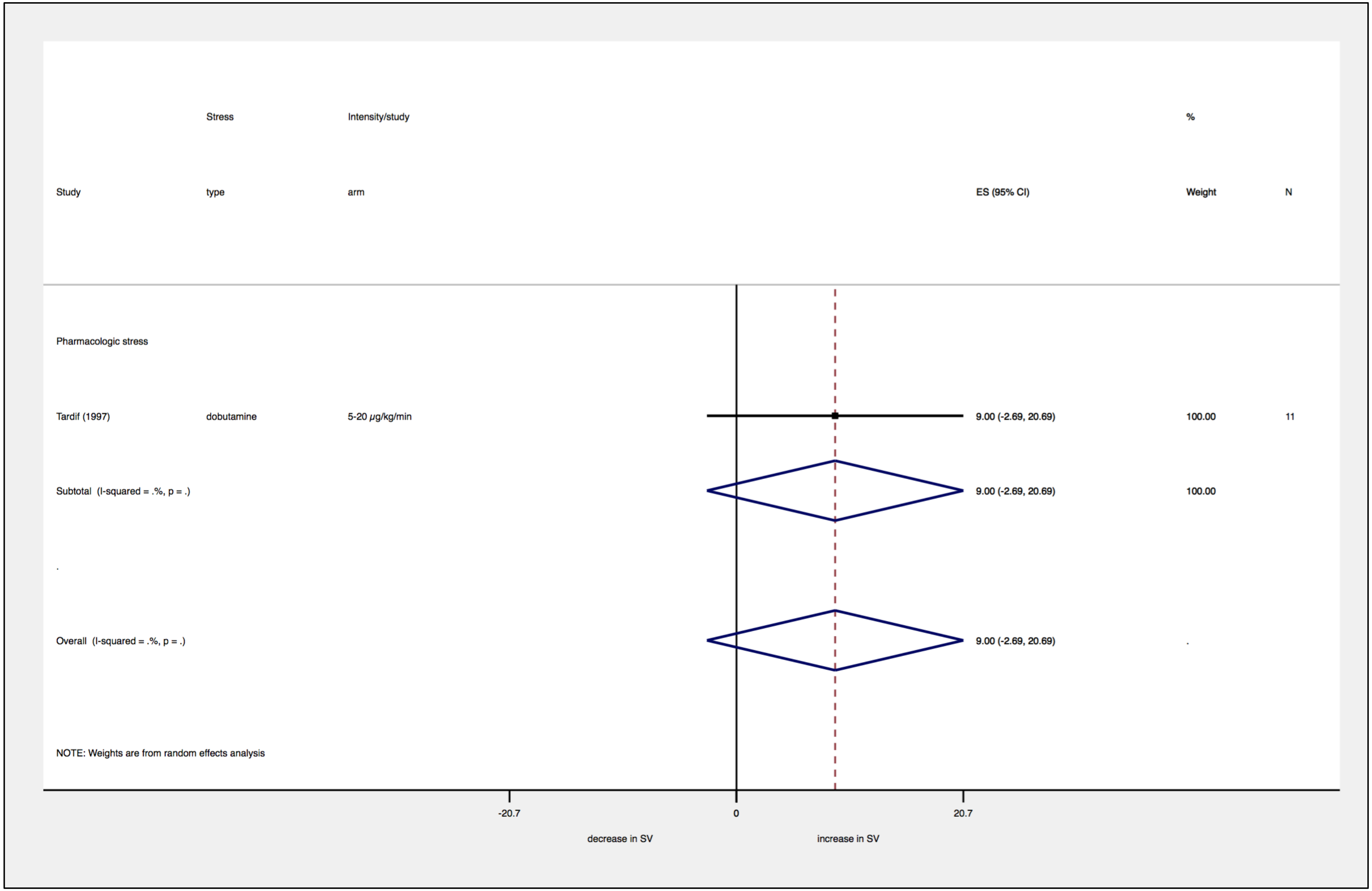

Figure S15 (b). SV Changes [ml] in patients with aortic stenosis after excluding low-flow, low-gradient patients for moderate intensity stress.

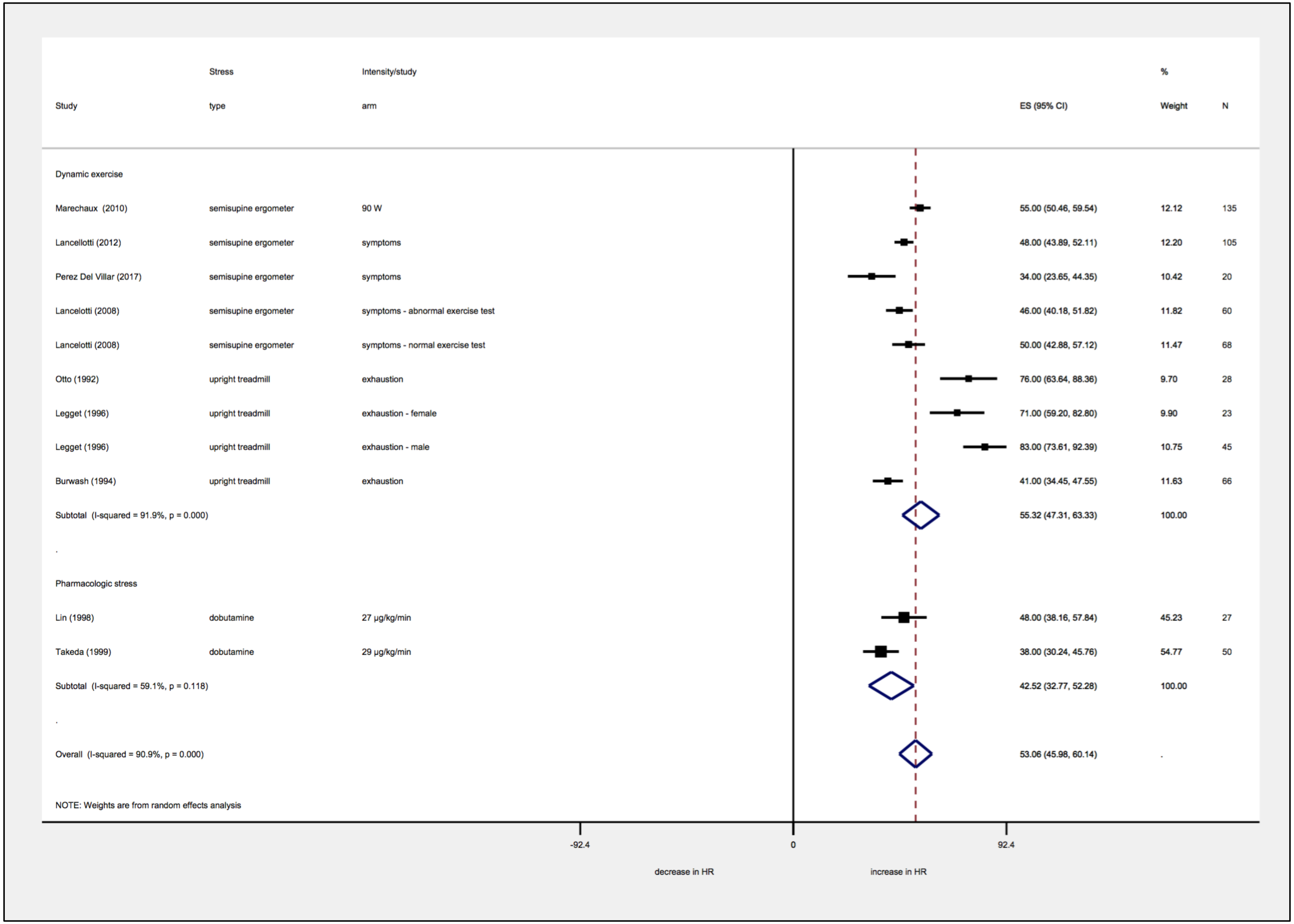

Figure S16. HR Changes [bpm] in patients with aortic stenosis for high intensity stress.

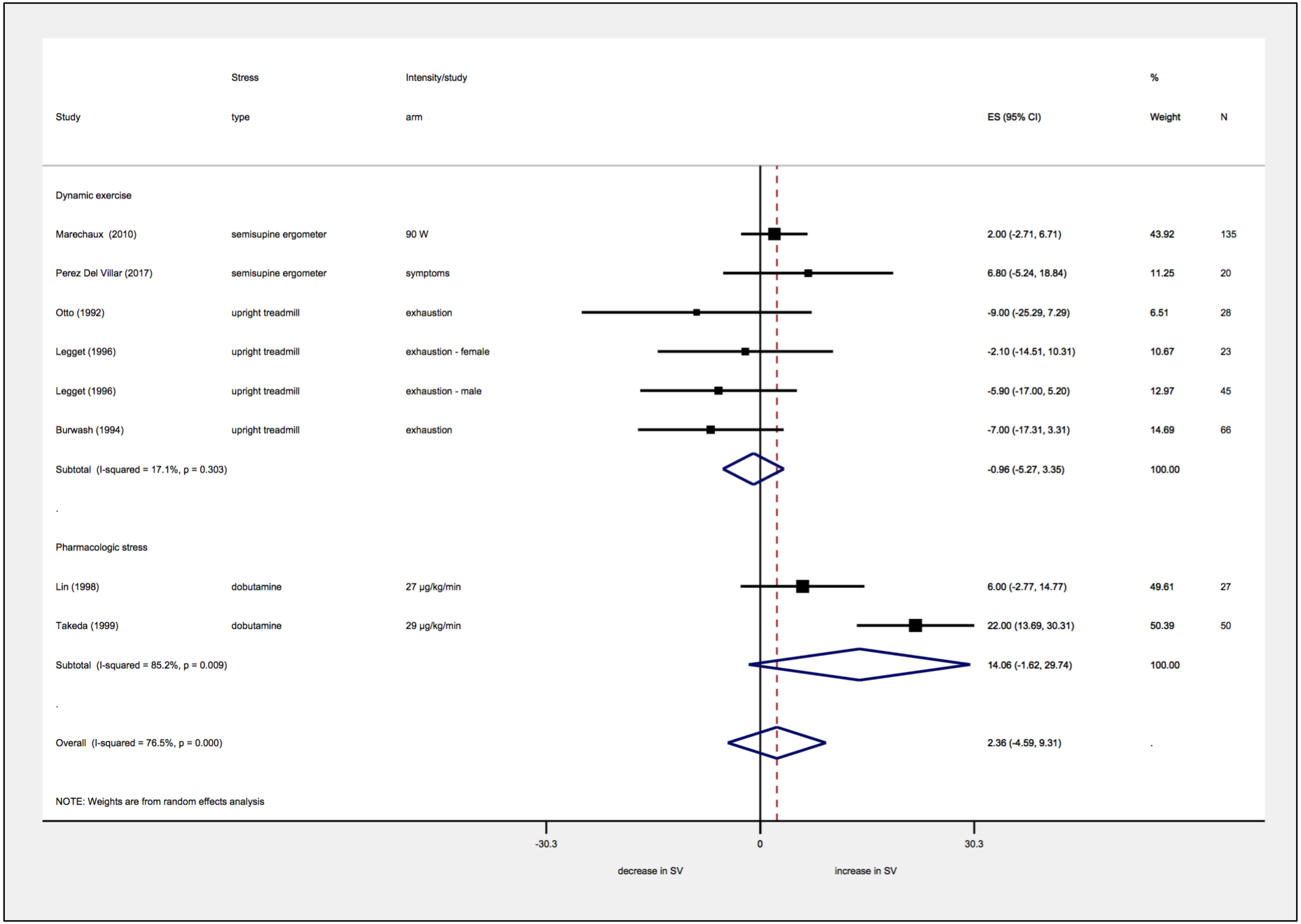

Figure S17. SV Changes [ml] in patients with aortic stenosis for high intensity stress.

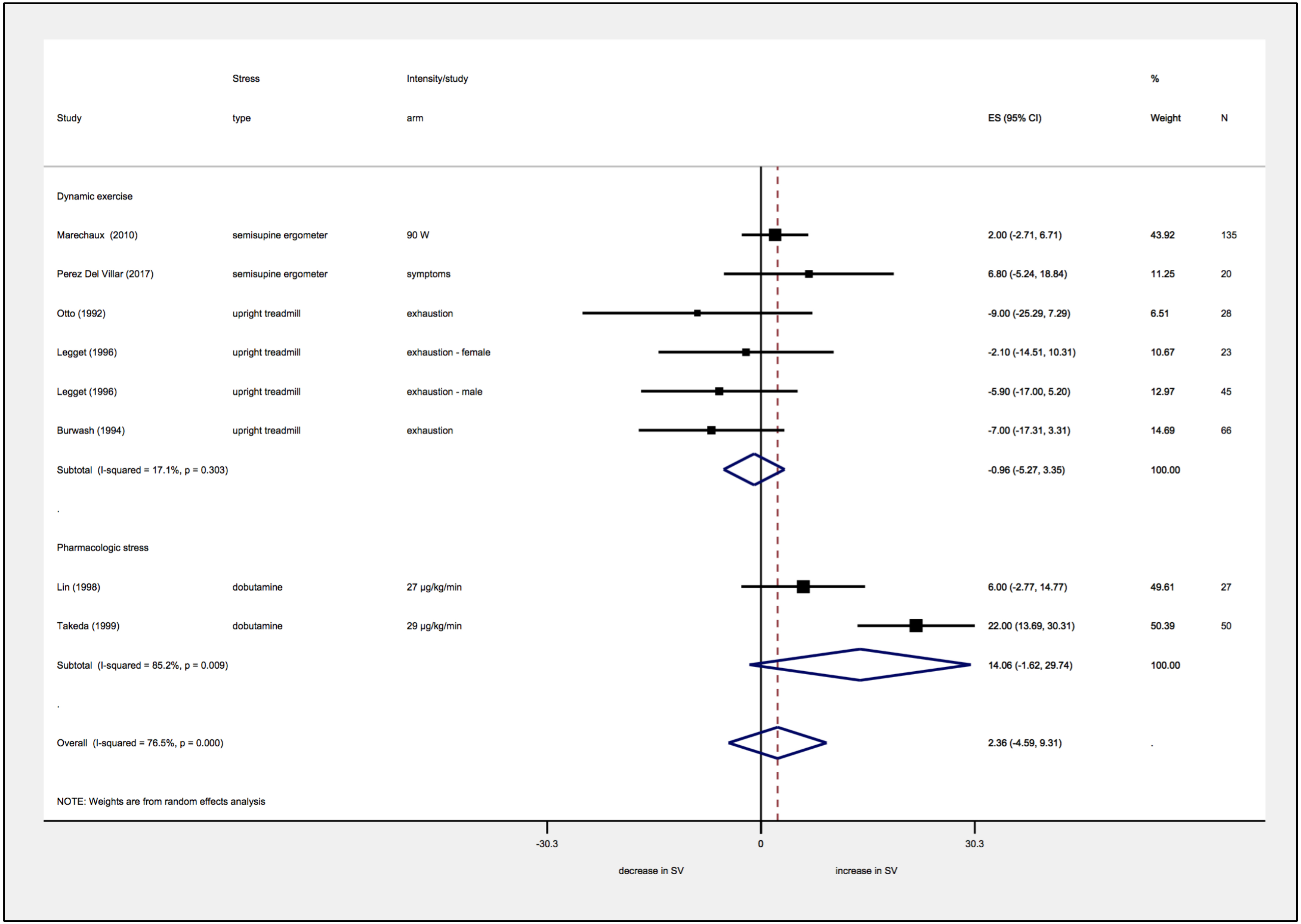

Figure S18. CO Changes [l/min] in patients with aortic stenosis for high intensity stress.

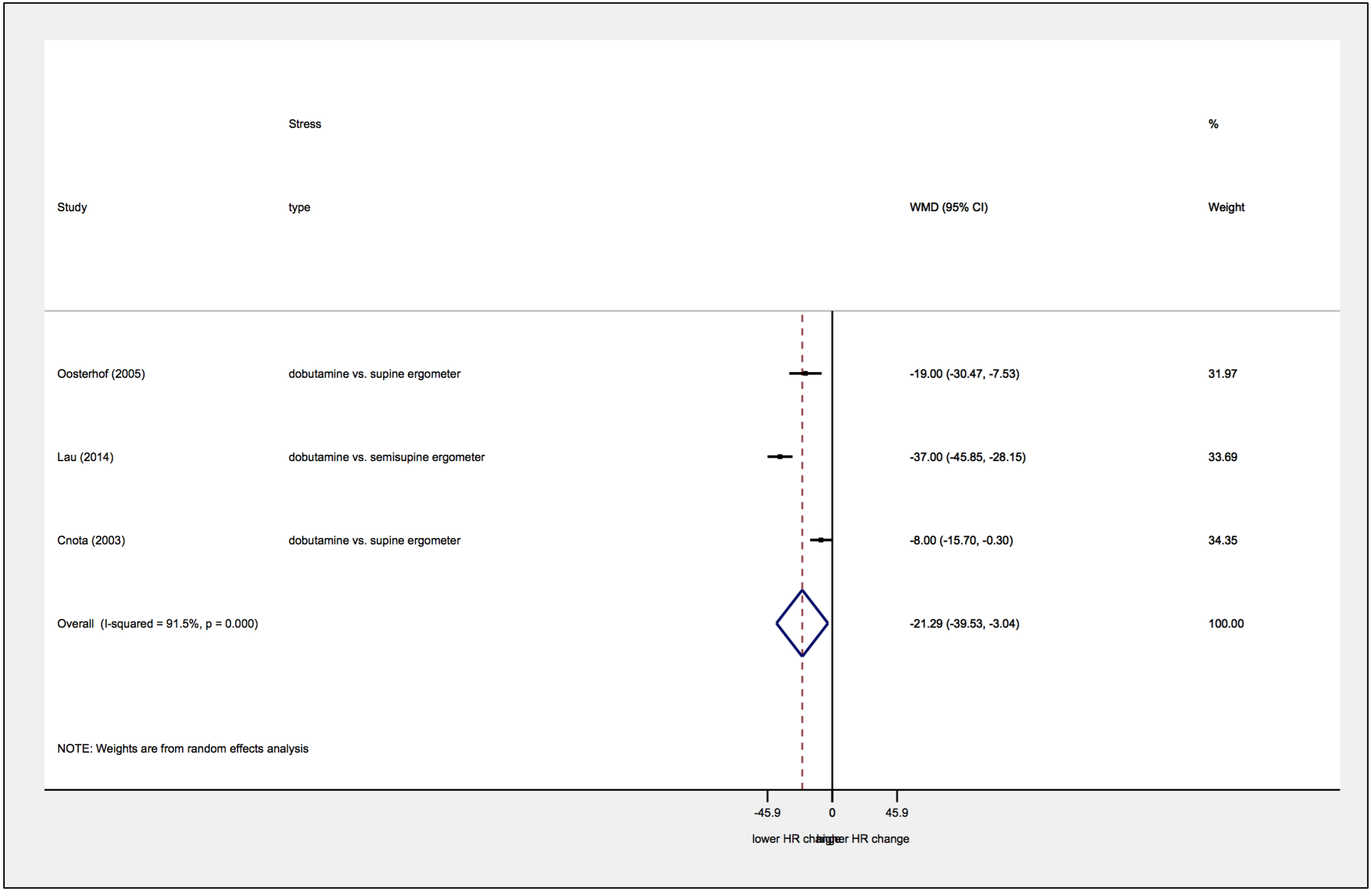

Figure S19. Differences in HR Changes [bpm] in healthy subjects for dobutamine vs. dynamic stress testing in directly comparative studies.

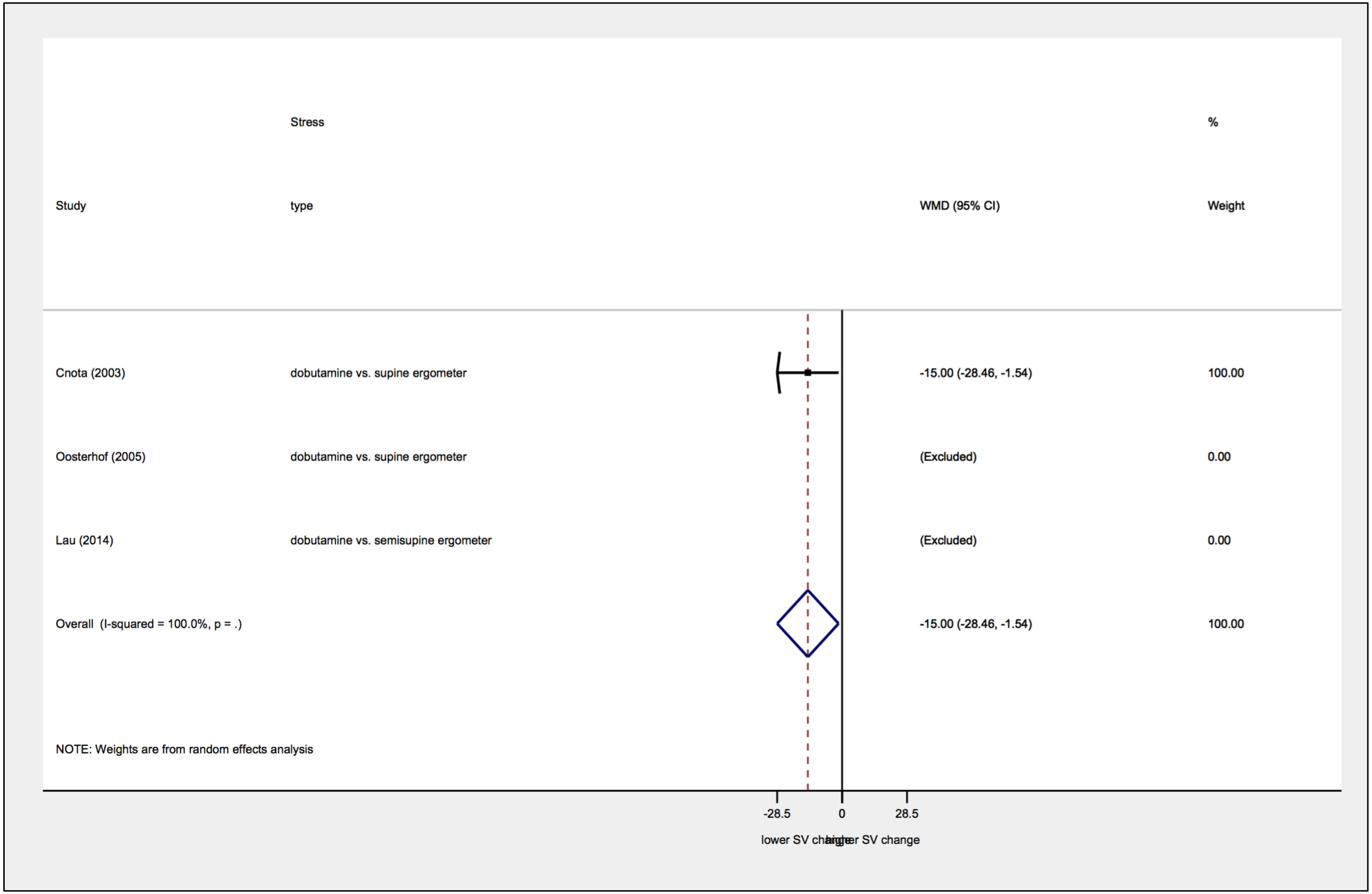

Figure S 20. Differences in SV Changes [ml] in healthy subjects for dobutamine vs. dynamic stress testing in directly comparative studies.

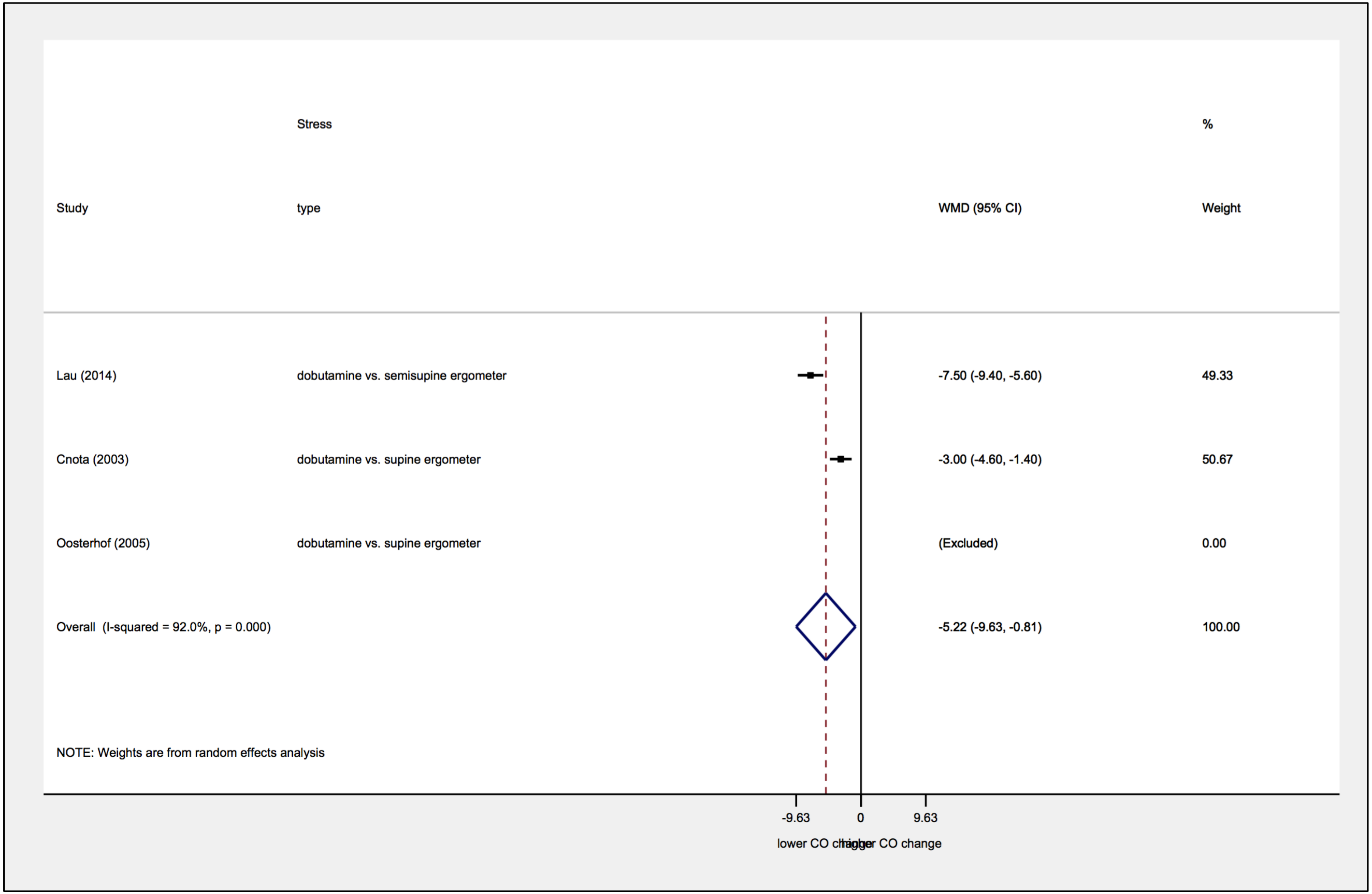

Figure S 21. Differences in CO Changes [l/min] in healthy subjects for dobutamine vs. dynamic stress testing in directly comparative studies.

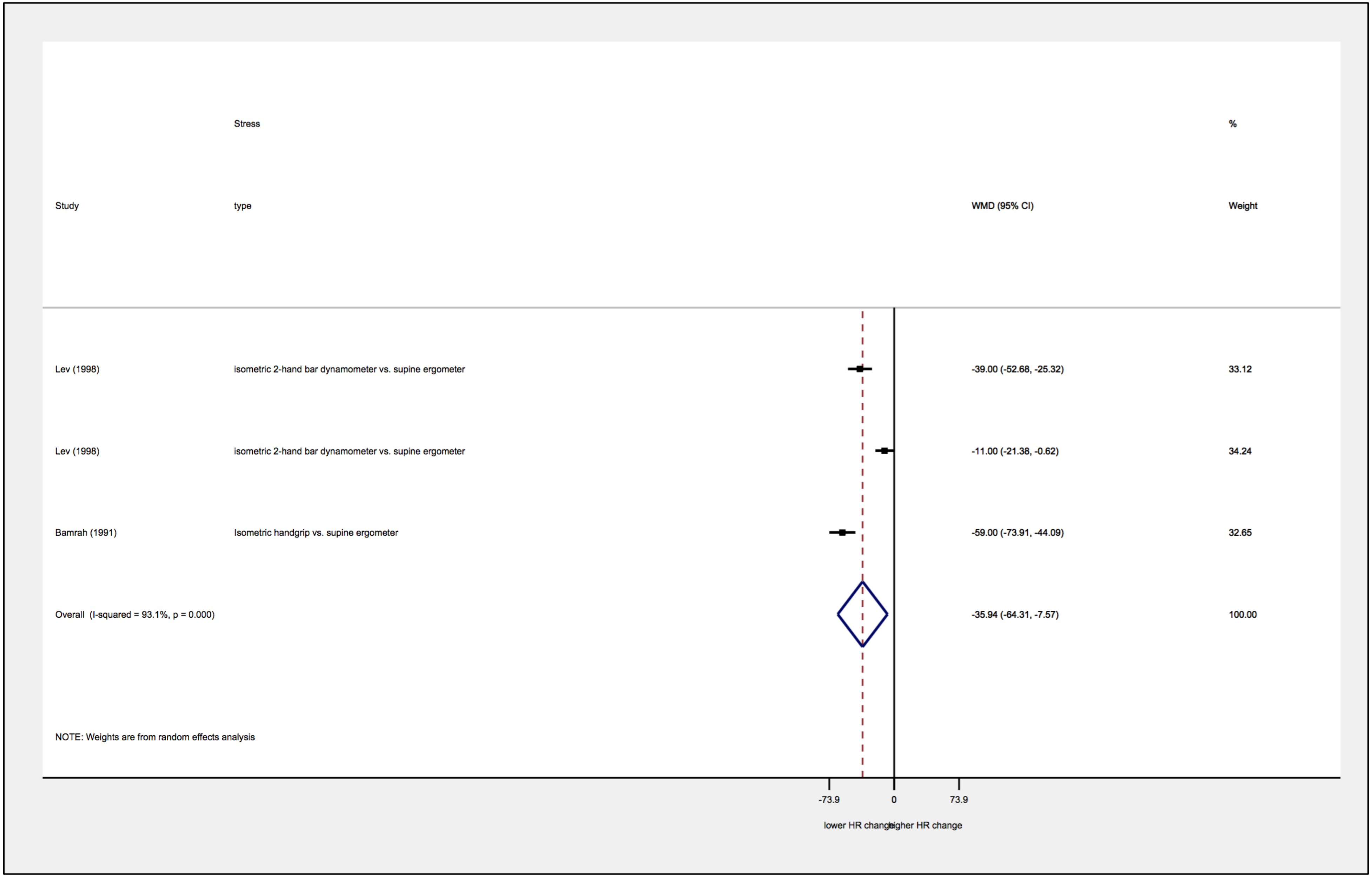

Figure S 22. Differences in HR Changes [bpm] in healthy subjects for isometric vs. dynamic stress testing in directly comparative studies.

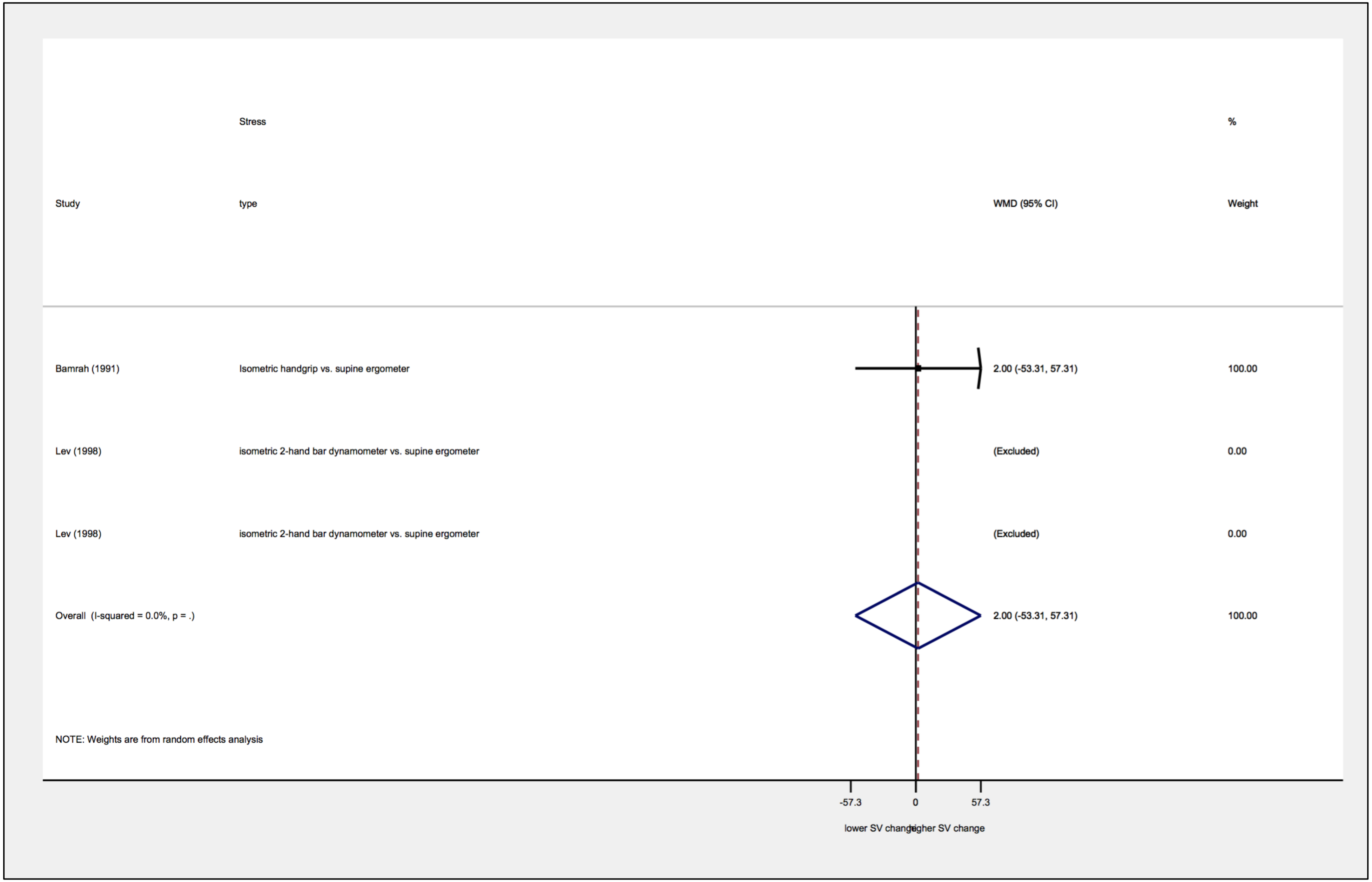

Figure S 23. Differences in SV Changes [ml] in healthy subjects for isometric vs. dynamic stress testing in directly comparative studies.

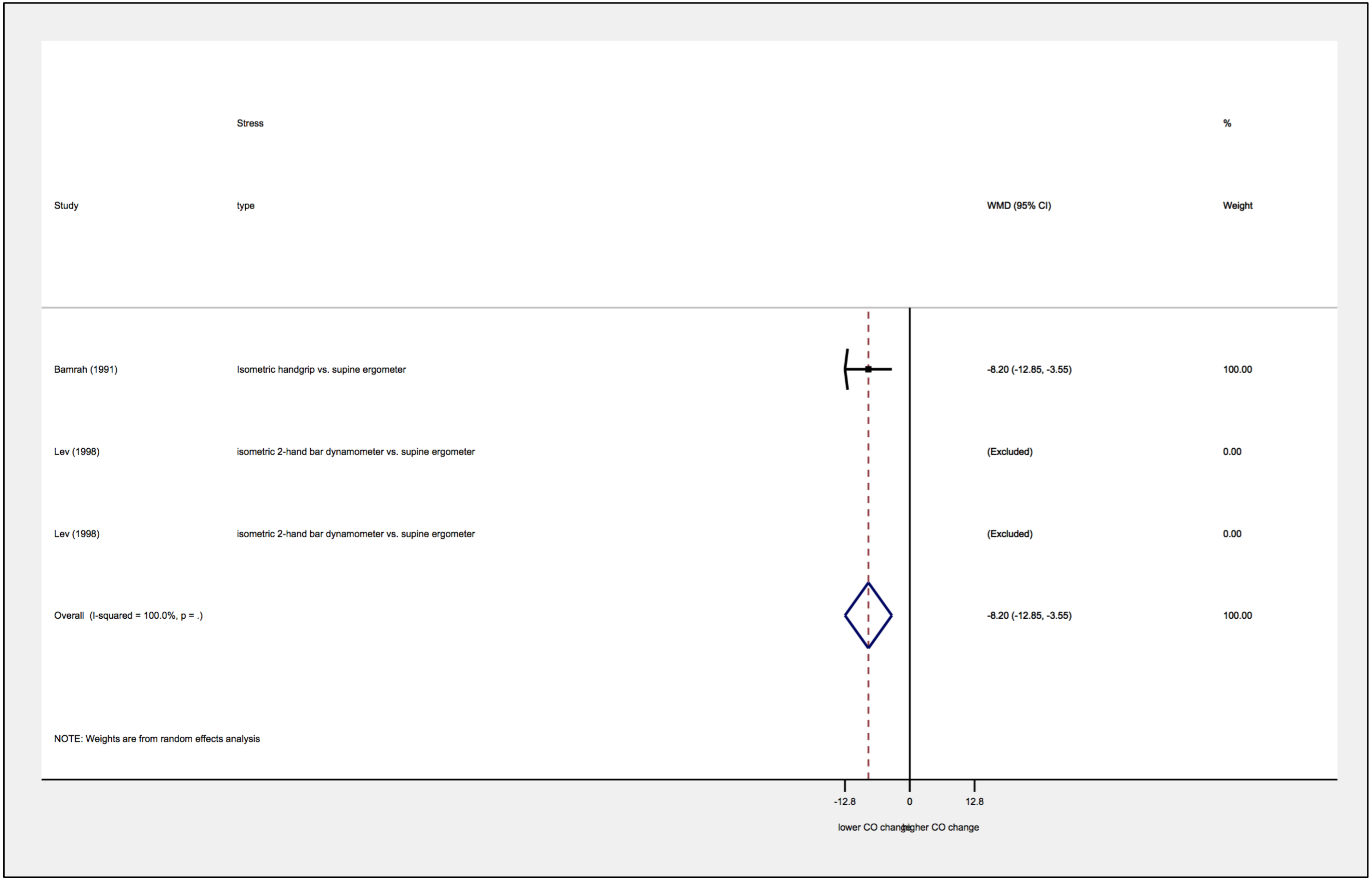

Figure S 24. Differences in CO Changes [l/min] in healthy subjects for isometric vs. dynamic stress testing in directly comparative studies.

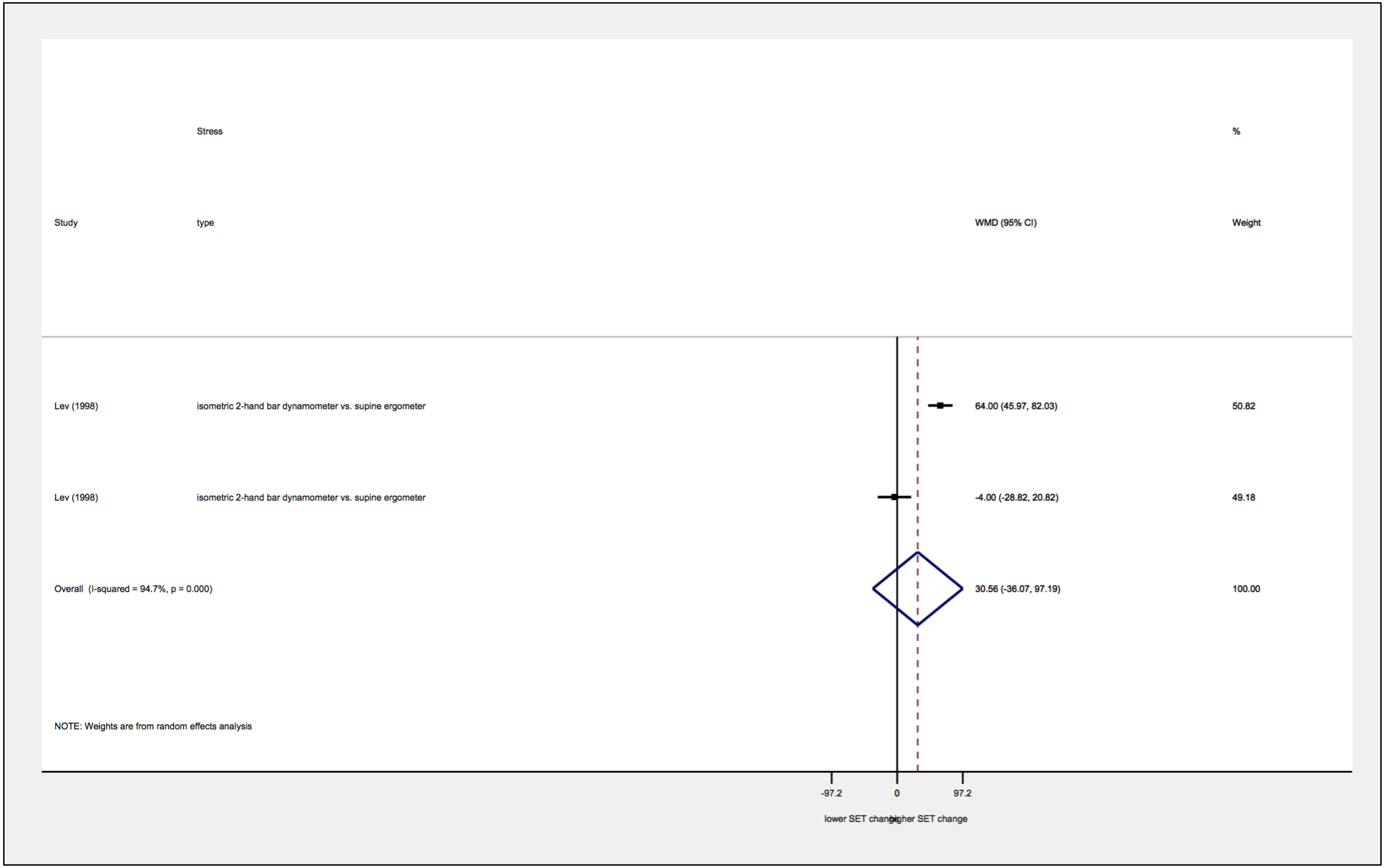

Figure S 25. Differences in SET Changes [ms] in healthy subjects for isometric vs. dynamic stress testing in directly comparative studies.
